# Friendships are more group‐oriented in the United Kingdom than in Japan

**DOI:** 10.1111/bjso.70040

**Published:** 2026-01-16

**Authors:** Philip Howlett, Gülseli Baysu, Tomas Jungert, Anthony P. Atkinson, Shushi Namba, Wataru Sato, Kumpei Mizuno, Magdalena Rychlowska

**Affiliations:** ^1^ Department of Psychology University of Bath Bath UK; ^2^ School of Psychology Queen's University Belfast Belfast UK; ^3^ Department of Psychology Lund University Lund Sweden; ^4^ Department of Psychology Durham University Durham UK; ^5^ Department of Psychology Hiroshima University Hiroshima Japan; ^6^ Psychological Process Research Team RIKEN Soraku‐gun Japan; ^7^ Department of Education Hokkaido University of Education Sapporo Japan

**Keywords:** culture, friendship, groups, relational mobility, socializing

## Abstract

Friendship is a common and complex social bond. Among friendship practices yet to be fully understood are group‐ versus dyadic‐oriented friendship styles, or whether people socialize with one versus multiple friends at a time. We report two studies comparing friendship styles and relational mobility among 1674 young adults (18–35 years old) in Japan and the United Kingdom. Respondents from both countries completed the Friendship Habits Questionnaire, a new measure of dyadic‐ versus group‐oriented friendship styles. Participants also estimated their friendship group size and time spent in friendship groups versus dyads, and completed a scale of relational mobility. Participants' group‐oriented friendship style, assessed with the Friendship Habits Questionnaire, was associated with larger friendship groups and more time spent in groups, rather than dyads of friends. Compared to Japanese, participants from the United Kingdom had more group‐oriented friendship styles and were more relationally mobile. Moreover, group‐oriented friendship styles were associated with higher relational mobility. These findings provide insights into models of friendship and social relationships promoted across diverse cultural settings.

## INTRODUCTION

Western definitions of friendship describe it as a voluntary (Fehr, 1996; Hartup, 1975; Wright, 1984), intimate, non‐kin relationship between two people who expect equal and reciprocal treatment to one another (e.g. Youniss, 1982). However, a closer look reveals that, although friendship networks are mostly made up of dyads (Dunbar, 2018; Peperkoorn et al., 2020), humans still spend time in groups, with peer groups strongly influencing our social and personal development (Harris, 1995).

Social behaviours and mental processes are likely to differ during interactions with one versus multiple friends. For example, a multi‐party conversation, which involves turn‐taking, listening to more than one person and attending to eye gaze and facial expressions of multiple people, creates different perceptual and cognitive demands than interacting with just one individual. Importantly, the number of friends with whom people socialize at the same time differs across individuals and cultures (French et al., 2006; Hinde, 1987). However, studies examining the size of friendship groups are rare. One reason for this scarcity is that people's social lives are irregular and highly variable. For example, 52% to 65% of people in the UK report seeing their friends from once a week to less than once a month (Opinium, 2025; Oxford Economics & National Centre for Social Research, 2018). The frequency of meeting friends is shaped by constraints such as time, energy, finance, distance and competing responsibilities (e.g. work and family). To account for this variation and accurately reflect a person's friendship group, diary studies need to be conducted over long periods of time. Moreover, asking respondents to estimate the number of friends they socialize with might be unreliable because people tend to forget as much as 40% of their social contacts (Fischer & Offer, 2020) and may not even remember their best friends (Brewer & Webster, 2000). As both behavioural and self‐reported assessments of friendship groups present challenges, a reliable measure of socializing with a single friend versus with multiple friends remains to be developed.

The Friendship Habits Questionnaire (FHQ; Howlett et al., 2023), also identified as an indicator of friendship styles, is a new scale designed to assess tendencies to connect in friendship dyads versus groups. The 23‐item questionnaire measures extraversion, self‐disclosure, identification with friendship groups and negative affect towards friendship groups. The FHQ questions target a person's behaviours (e.g. *I am talkative when I am in larger groups of friends; My friends and I tell each other secrets*), beliefs (e.g. *I identify with a friendship group*) and affect (e.g. *I feel uneasy with members of friendship groups*) towards friendship groups. These dimensions are indicative of group‐oriented friendship behaviour (Howlett et al., 2023): extant research suggests that extraversion is associated with larger, more interconnected social networks (Selden & Goodie, 2018). Compared to non‐friends, people involved in reciprocal friendships are more likely to identify with friendship groups and feel more positive towards such groups (Kwon & Lease, 2009). Self‐disclosure is needed to establish close friends in general (Fehr, 2004; Kaufmann et al., 2022) and extraverts disclose more than introverts (White et al., 2004; Wilson et al., 2015). Previous research suggests that Friendship Habits Questionnaire scores meaningfully correlate with other measures of socializing in groups versus dyads, such as participants' reports of the size of their friendship groups and the number of friends present in their social interactions over a period of 3 months (Howlett et al., 2023). To date, the number of friends with whom people usually socialize and the validity of the FHQ have only been investigated in one study, where participants completed the English version of the FHQ (Howlett et al., 2023). Thus, little is known about cultural differences in friendship styles and about the cross‐cultural validity of this questionnaire.

Such differences are to be expected because extant research reveals substantial cultural variation in friendship group sizes and the way people socialize with friends. Western definitions of friendship describe it as voluntary (e.g. Fehr, 1996), but in Eastern cultures friendships can be perceived as pre‐determined rather than chosen (Baumgarte, 2013; Hofstede, 1980). Western definitions also mention friends having equal status, but expressions describing friendships in some cultures (e.g. *big sister*, *little brother* in India) suggest hierarchical family type bonds. While definitions of friendship also refer to relationships between *two* people, friends may spend time together in dyads or in groups, and these tendencies differ across cultures (Chen & French, 2008). For example, Russians are encouraged to be part of groups (Moore, 2020). Indonesians value having good relationships with everyone in their community while Australians are more likely to have a best friend (Noesjirwan, 1978). Koreans tend to be more dyadic than Indonesians (French et al., 2006). Dutch children play in dyads more than Spanish children (Goudena & Sénchez, 1996). When American adolescents were asked to list their school friends, approximately 18% of respondents belonged to friendship groups (structures where two participants had nominated two or more mutual friends; Henrich et al., 2000). A similar study conducted in China showed that as many as 48% of Chinese adolescents are members of groups (structures where three or more participants shared over 50% of their friend nominations; Liu & Chen, 2003). Overall, despite some evidence of cultural variation in friendship group sizes, much less is known about friendship styles, understood as a more enduring orientation towards group versus dyadic friendships. Furthermore, an explanatory framework for such cross‐cultural variation in friendship practices and styles is currently lacking.

The cross‐cultural findings described above suggest a link between socializing in friendship dyads versus groups and the sociological construct of relational mobility (Thomson et al., 2018). Relational mobility is directly relevant to friendships as it describes the degree to which individuals within a society have the freedom to voluntarily form, maintain and dissolve interpersonal relationships based on personal preference. Perhaps explaining some of the cultural differences in friendship group size mentioned earlier (e.g. French et al., 2006), countries like Australia, the United States and the Netherlands, appearing to promote dyadic friendships, are also described as cultures high in relational mobility (Thomson et al., 2018). Relational mobility is associated with intimacy and self‐disclosure, traits important for maintaining friendship bonds (Kito et al., 2017; Thomson et al., 2018; Yamada et al., 2015). Previous research links relational mobility with higher extraversion, higher openness and lower neuroticism (Jokela, 2021), suggesting that personality traits are important to perceived opportunities to make friends. Relational mobility is also linked with flexibly leaving or changing groups. Low relational mobility has been associated with more stable relationships (e.g. Schug et al., 2010) whereas higher relational mobility is associated with greater network integration (Li et al., 2023), where one's close contacts are likely to know each other. Higher relational mobility is also associated with a greater number of people one can seek advice from (Horita et al., 2025).

Importantly, societies low in relational mobility tend to be collectivistic (Thomson et al., 2018). Collectivism is a cultural orientation in which people prioritize in‐group goals, social harmony and interdependence, viewing the self as fundamentally connected to close others and larger in‐groups (e.g. Hofstede, 1980; Triandis et al., 1988). In such societies, dyadic friendships could potentially be perceived as threatening the interests of the collective (Sharabany, 2006). Relational mobility is also positively correlated with residential mobility (Thomson et al., 2018), a dimension describing how frequently people move or intend to move. Thus, members of residentially mobile societies, similar to people high in relational mobility, tend to seek new groups. Conversely, members of residentially stable societies commit more to the groups they are in (Oishi et al., 2015). These facets of relational mobility highlight its relevance to maintaining friendships and make it an interesting dimension to study in the context of group‐ versus dyadic‐oriented friendship styles.

Given that a group‐oriented friendship style involves identifying with a friendship group (Howlett et al., 2023) and requires paying attention to multiple faces and conversation streams, constructs describing cognition and perception might also be useful in explaining cross‐cultural variation in friendship styles. Holistic and analytic thinking describe how people perceive visual scenes and allocate attention (Nisbett et al., 2001). Analytic thinkers perceive a focal point of interest without giving much weight to the surrounding context. Conversely, holistic thinkers perceive objects within the context in which they are presented. Research shows members of cultures promoting holistic thinking styles, such as Japan, utilize contextual information more than members of cultures promoting analytic thinking, such as the United States. For example, in studies where participants see a smiling person surrounded by other people and are asked to judge how this person feels, compared to American observers, Japanese participants are more influenced by the facial expressions displayed by the surrounding people (e.g. Masuda et al., 2008), which may have implications for social relationships. These results dovetail with research suggesting that members of East Asian cultures, like Japan, tend to see people as embedded in a larger whole (Oyserman et al., 2002) and emphasize the need to consider the group context to successfully navigate one's social world (de Oliveira & Nisbett, 2017). Since such findings suggest that holistic thinkers have greater ease in processing multiple social signals, they may also indicate a tendency for group‐oriented friendships among holistic thinkers. Consistent with such reasoning, holistic thinking is also linked with low relational mobility and collectivism (San Martin et al., 2019; Thomson et al., 2018). Greater fluency with processing larger and more complex social environments may also be helpful in maintaining group harmony (Kavanagh & Yuki, 2017). Thus, both thinking styles and relational mobility appear relevant to friendship styles and practices and offer a promising starting point to explore cultural variation in how people socialize with friends.

### Current study

Although existing research suggests that cultures differ in the extent to which they promote dyadic versus group friendships, such differences have not been systematically studied using similar core measures. Investigating this variation and examining the cross‐cultural validity of the Friendship Habits Questionnaire is the goal of the present research. Specifically, we examine group‐ versus dyadic‐oriented friendships in the United Kingdom and Japan. These two cultures were selected to vary on multiple cultural dimensions, including relational mobility (Thomson et al., 2018) and analytic versus holistic thinking (Nisbett et al., 2001). Compared to Japan, the United Kingdom is a culture of higher relational mobility (Thomson et al., 2018), promoting more analytic thinking styles. The two countries are also collectivistic (Japan) and individualistic (UK, Hofstede et al., 2010; Kitayama et al., 2009). To date, no study has compared the United Kingdom and Japan on group‐oriented friendship styles, making it hard to form firm predictions about the size of friendship groups in both countries. Previous findings show that classroom sizes in Japan are bigger than those in the United Kingdom (Betts et al., 2014). Children and adolescents in Japan report having more friends in their class and year group than children in England (Eslea et al., 2004; Kanetsuna & Smith, 2021) and the USA (White, 1993). The average friendship group size reported by Japanese university students is around 4, with some groups reaching as many as 10 people (Mutoh et al., 2016). There are also specific words in Japanese used to describe friendship groups such as *nakama*, describing a group of friends that someone associates with, which could include work colleagues or hobby groups (Cargile, 1998). Together, existing research suggests that Japan and the United Kingdom promote different ways of socializing with friends but there are no tests of whether this translates to friendship practices and styles. Due to the lack of such findings, we did not put forward specific hypotheses regarding the direction of differences between the United Kingdom and Japan.

In two studies, participants from both countries completed the Friendship Habits Questionnaire, a measure of group versus dyadic friendship styles and reported their socializing practices with dyads and groups of friends (i.e. the size of their friendship groups and time spent in friendship groups). They also completed scales of relational mobility (Thomson et al., 2018) and thinking styles (Choi et al., 2007; Study 1). Consistent with previous findings (Howlett et al., 2023), we predicted positive associations between FHQ scores (indicating a more group‐oriented friendship style) and self‐reports of socializing in larger friendship groups and spending more time in friendship groups versus dyads. We also examined cultural differences in group‐ versus dyadic‐oriented friendship styles as well as associations between friendship styles, relational mobility and holistic thinking. Consistent with existing research, we predicted that Japanese participants would report lower levels of relational mobility (Thomson et al., 2018) and more holistic thinking (San Martin et al., 2019) than participants from the United Kingdom.

## STUDY 1

### Method

All materials, data and analyses can be found at: https://osf.io/hfma6/overview.

#### Participants

We aimed to recruit a minimum of 500 participants per country, comparable to other cross‐cultural studies (e.g. Thomson et al., 2018) and exceeding the minimum of 400 recommended for structural equation models (Fabrigar et al., 1999). A post hoc power analysis suggested a minimum of 88 participants per country to achieve 95% power for independent‐samples *t*‐tests with medium effect sizes (.50; G*Power, Faul et al., 2007). Respondents in Japan (*n* = 646) and the United Kingdom (*n* = 715) were invited to participate and opened the study link. A total of 527 Japanese participants (age *M* = 29.71, *SD* = 4.40, range: 18–35; 348 female, 2 other) and 518 participants from the United Kingdom (age *M* = 22.01, *SD* = 3.99, range: 18–35; 418 female, 11 other) were included in the analyses as they completed most of the survey, passed at least one of the two attention checks included and confirmed they lived in the target country (119 responses in Japan and 197 responses in the United Kingdom were thus excluded). Most of the Japanese respondents (*n* = 508) were recruited on the research platform CrowdWorks (2021) and paid 330 Yen for their help. The remaining participants (*n* = 19) were volunteers recruited via email at a Japanese university. Most of the UK sample (*n* = 309) was recruited at two university campuses and compensated with course credits. Other participants (*n* = 209) accessed the survey via online platforms, including Facebook, WhatsApp and SurveyCircle (2021), and completed the questionnaire voluntarily.

Japanese participants predominantly described themselves as East Asian (*n* = 516) whereas the UK participants were predominantly White (*n* = 411). This is in line with approximately 98% of Japanese population identifying as Japanese (Central Intelligence Agency, 2021) and approximately 74% of the UK population identifying as White (UK Office of National Statistics, 2021; see also Supporting Information S1).

#### Procedure and materials

This was an online survey implemented in Qualtrics (2021, Provo, UT). After reading an information page and providing consent, respondents provided demographic information and completed the study questionnaire. The order of items within each scale was randomized. Two attention checks (one embedded in the FHQ) asked participants to pick a specific response option, for example: *Please select the option labelled slightly agree for this statement*.

##### Group‐versus dyadic‐oriented friendship styles

The Friendship Habits Questionnaire (FHQ; Howlett et al., 2023, Supporting Information Table S1a) is a 23‐item questionnaire measuring extraversion in groups (e.g. *I am full of energy when I am in larger groups of friends*), self‐disclosure with one's friendship network (e.g. *My friends and I tell each other secrets*), identification with friendship groups (henceforth called: positive group identification, e.g. *I feel strong ties to a friendship group*) and the negative affect towards friendship groups (reverse‐scored, henceforth called: negative group identification, e.g. *I feel uneasy with members of friendship groups*). FHQ items are answered on 7‐point Likert scales. Higher scores indicate more group‐oriented friendship styles. FHQ had excellent reliability (overall: *α* = .94, *ω* = .94; Japan: *α* = .93, *ω* = .93; UK: *α* = .92, *ω* = .92). Given the cross‐cultural differences in perceptions of who is a friend (e.g. Baumgarte, 2013; Fehr, 1996; Hofstede, 1980), instructions of the questionnaire did not provide participants with a definition of friendship. Instead, we used measurement invariance testing to ensure that the FHQ measures the same concept in both countries (Putnick & Bornstein, 2016).

##### Self‐reported friendship practices

We measured the size of friendship groups by asking participants to estimate the number of friends they usually socialize with at the same time. This was a numeric open question. We also measured the proportion of time respondents spent in friendship groups. Participants reported how they spent time with friends on a sliding scale ranging from 0% (always with one friend) to 100% (always with 2 or more friends).

As an exploratory measure, we included quantity of friends. Participants were shown a diagram of five concentric circles labelled (from the central to outer circle): *You, Extremely Close Friends, Close Friends, Friends That Are Not Close and Acquaintances*, and asked to write down the initials of their friends for each of the four friends and acquaintances categories. The total number of friends (the sum of initials across the four friend categories) was strongly and positively correlated (*r* = .96, *p* < .001) with the number of generally close friends (the sum of initials of extremely close and close friends). We therefore retained the total number of friends as an index of friendship quantity.

##### Relational mobility

The 12‐item Relational Mobility Scale (RMS; Thomson et al., 2018) measures the perception that the respondent's society allows its members to meet new people and choose their own friends. Questions capture two dimensions: “meeting”, reflecting the opportunities to forge new relationships, and “choosing”, reflecting the freedom to choose social relationships based on one's preferences. Items are answered on a 6‐point Likert scale. RMS scores range from 1 to 6, with higher scores indicating greater relational mobility. The RMS had good reliability (overall: *α* = .86, *ω* = .86; Japan: *α* = .85, *ω* = .85; UK: *α* = .85, *ω* = .85).

##### Thinking styles

The Analysis‐Holism Scale (AHS; Choi et al., 2007) contains 24 items and measures thinking styles. Items are answered on a 7‐point Likert scale. Scores range from 1 to 7 and higher values indicate more holistic thinking, defined as a tendency to pay more attention to context, rather than focusing on specific phenomena. The AHS had acceptable reliability (overall: *α* = .71, *ω* = .74; Japan: *α* = .74, *ω* = .77; UK: *α* = .68, *ω* = .70).

### Results

#### Analytic strategy

We analysed the data using the “jmv” and “lavaan” packages in RStudio (RStudio Team, 2021, version 1.4.1717), focusing on the relationships between friendship styles measured by FHQ scores, self‐reported friendship practices, relational mobility and thinking styles. We also examined cultural differences between Japan and the United Kingdom.

Predictions were tested using a double analytic approach. First, we used Spearman rho correlations and *t*‐tests, or equivalent non‐parametric tests, to examine associations between variables and to compare friendship styles, relational mobility and thinking styles across countries. As the FHQ, RMS and AHS scales include multiple factors, we also tested the study predictions with a structural equation model (SEM) approach. An advantage of this method is the possibility of assessing the measurement invariance of each scale before hypothesis testing. Achieving at least partial scalar measurement invariance indicates that questionnaires are interpreted similarly across cultures (Putnick & Bornstein, 2016). The final SEM model included the partial scalar invariance models of FHQ and RMS (see the ‘Measurement Invariance’ section below), as well as the proportion of time in groups, size of friendship groups and age as single‐item measures defined at the indicator level. All variables were allowed to correlate. The overall model fit was acceptable (Supporting Information S6).

Existing findings indicate age is associated with a decrease in friendship contact and number of friends (e.g. Gillespie et al., 2015). Moreover, friendship group size has been shown to vary depending on gender (Benenson, 2019). We therefore conducted a series of ANCOVAs including age, gender and socio‐economic status as covariates to control for demographic differences across samples (see Supporting Information S7).

#### Measurement invariance

All scales underwent measurement invariance testing before being added to the SEM. The Friendship Habits Questionnaire and the Relational Mobility Scale achieved partial scalar invariance (Supporting Information S2 and S4). Both measurement invariance models included common bias factors to control for differences in participants' tendency to choose extreme values or always agree on Likert scales (e.g. Fischer et al., 2009).

For FHQ measurement invariance, the four first‐order factors (extraversion, self‐disclosure, positive group identification and negative affect towards friendship groups) loaded onto the second‐order factor of group‐oriented friendship style (FHQ). Thus, a group‐oriented friendship style is indicated by higher scores on extraversion, self‐disclosure and positive group identification, and lower scores on negative affect towards friendship groups. Although one might intuitively expect associations between self‐disclosure and dyadic friendship styles, the findings of previous research (Howlett et al., 2023) as well as the results of the current studies indicate the opposite. In addition, separating self‐disclosure from the second‐order factor of FHQ significantly worsens the model (Supporting Information Table S1b).

The RMS measurement invariance model comprised two first‐order factors (meeting and choosing) loading onto the second‐order factor of relational mobility (RMS). Thus, relational mobility is indicated by higher scores on both meeting and choosing.

The Analysis‐Holism Scale did not achieve the configural level of measurement invariance (Supporting Information S5) and was thus not included in the model.

#### Friendship styles and self‐reported friendship practices

We predicted that group‐oriented friendship styles, indicated by higher FHQ scores, would be associated with more time spent in friendship groups and larger sizes of friendship groups. Correlation analyses indeed revealed significant positive associations in the entire sample and within each culture (Tables 1 and 2). This finding was replicated using the Structural Equation Model (Table 3).

**TABLE 1:** Study 1: Descriptive statistics and spearman correlations between measures of friendship styles, friendship practices (more time spent in friendship groups and larger sizes of friendship groups), relational mobility, thinking styles and demographics (entire sample).

Variable	*n*	*M*	*SD*	1	2	3	4	5	6	7	8
1. FHQ	1045	4.36	1.04	–							
2. Proportion of time in groups	1027	41.32	27.63	.47***	–						
3. Friendship group size	1026	2.17	1.57	.47***	.60***	–					
4. Friendship quantity	1045	3.47	2.66	.20***	.14***	.16***	–				
5. Relational mobility	1031	3.84	0.68	.31***	.18***	.23***	.14***	–			
6. Thinking styles	1034	4.81	0.45	.16***	.06	.05	.10**	.11***	–		
7. Age	1045	25.90	5.70	−.33***	−.29***	−.30***	−.22***	−.20***	−.14***	–	
8. Socio‐economic status	1045	5.14	1.63	.28***	.25***	.21***	.05	.15***	.07*	−.26***	–

* *p* < .05.

** *p* < .01.

*** *p* < .001.

**TABLE 2:** Study 1: descriptive statistics and spearman correlations between measures of friendship styles, friendship practices (more time spent in friendship groups and larger sizes of friendship groups), relational mobility, thinking styles and demographics (Japanese and UK samples).

Variable	1	2	3	4	5	6	7	8
1. FHQ	–	.44***	.43***	.16***	.31***	.05	−.15***	.16***
2. Proportion of time in groups	.32***	–	.57***	.12**	.13**	.03	−.20***	.17***
3. Friendship group size	.33***	.52***	–	.12**	.14**	−.01	−.18***	.10*
4. Friendship quantity	.01	−.01	−.01	–	.03	.04	−.11*	.01
5. Relational mobility	.19***	.09*	.19***	.14**	–	.12**	−.05	.08
6. Thinking styles	.09*	−.05	−.05	.03	.01	–	.06	.05
7. Age	−.02	−.03	−.02	−.01	−.07	−.07	–	−.18***
8. Socio‐economic Status	.24***	.21***	.18***	−.07	.10*	−.03	−.07	–
Japan								
*M*	3.94	32.93	1.68	2.77	3.69	4.71	29.71	4.71
*SD*	0.95	26.29	1.21	0.98	0.64	0.41	4.40	1.60
UK								
*M*	4.78***	49.97***	2.67***	4.19***	4.00***	4.90***	22.01***	5.58***
*SD*	0.96	26.30	1.74	3.50	0.69	0.47	3.99	1.54

*Note*: Numbers below the diagonal represent correlations in Japan, numbers above the diagonal represent correlations in the United Kingdom. Asterisks denote significant pairwise comparisons.

* *p* < .05.

** *p* < .01.

*** *p* < .001.

**TABLE 3:** Study 1: Correlations (SEM) between measures of friendship styles, friendship practices (more time spent in friendship groups and larger sizes of friendship groups), relational mobility, and age (Japanese and UK samples).

Variable	1	2	3	4	5
1. FHQ	–	.50***	.40***	.40***	−.11*
2. Proportion of time in groups	.35***	–	.48***	.14**	−.17**
3. Friendship group size	.25***	.42***	–	.14**	−.13**
4. Relational mobility	.34***	.15*	.24***	–	−.01
5. Age	−.01	−.04	−.03	−.11*	–

*Note*: Numbers below the diagonal represent correlations in Japan, numbers above the diagonal represent correlations in the United Kingdom.

* *p* < .05.

** *p* < .01.

*** *p* < .001.

#### Friendship styles, relational mobility and thinking styles

We examined the associations between group‐ versus dyadic‐oriented friendship styles measured via FHQ, relational mobility and holistic (versus analytic) thinking styles. We observed a moderate positive correlation between relational mobility and FHQ scores in the entire sample and within each culture, indicating that respondents endorsing group‐oriented friendship styles reported higher relational mobility (Tables 1 and 2). Correlations derived from the SEM analysis supported these findings (Table 3).

Higher FHQ scores were positively correlated with holistic thinking in the entire sample. This association remained significant among Japanese, but not among participants from the United Kingdom. The correlation was not examined with SEM because the Analysis–Holism Scale failed to achieve configural invariance (Supporting Information S5).

#### Cultural differences in friendship styles and practices

We further compared the endorsement of group‐ versus dyadic‐oriented friendship styles among participants from the United Kingdom and Japan using the FHQ scores, measures of proportion of time in groups, and sizes of friendship groups. FHQ scores were significantly lower in Japan (*M* = 3.94, *SD* = .95) than in the United Kingdom (*M* = 4.78, *SD* = .96), *t*(1043) = −14.14, *p* < .001, *d* = −.87, suggesting that Japanese participants endorsed more dyadic‐oriented friendship styles. The SEM results supported the findings, with a significant intercept (mean) suggesting lower FHQ scores in Japan than in the United Kingdom (−.62, *p* < .001).

Proportion of time in friendship groups was also lower for Japanese participants (*M* = 32.93%, *SD* = 26.29) than for participants from the United Kingdom (*M* = 49.97%, *SD* = 26.30), *t*(1025) = −10.39, *p* < .001, *d* = −.65. The SEM results supported the finding, with a significant intercept reflecting lower proportion of time in groups among Japanese participants (−17.01, *p* < .001). Moreover, subsequent Welch's *t*‐tests showed that Japanese participants socialized in smaller groups (*M* = 1.68, *SD* = 1.21) than participants from the United Kingdom (*M* = 2.67, *SD* = 1.74), *t*(894.25) = −10.54, *p* < .001, *d* = −.66, replicated by the SEM model −.98, *p* < .001, and reported having fewer friends (*M* = 2.77, *SD* = 0.98) than participants from the United Kingdom (*M* = 4.19, *SD* = 3.50), *t*(595.58) = ‐8.92 *p* < .001, *d* = −.55 (Welch's *t*‐tests were used since the assumption of homogeneity was violated for the size of friendship groups, *F*(1, 1024) = 50.21, *p* < .001 and for friendship quantity, *F*(1, 1043) = 185.51, *p* < .001). Table 4 shows the numbers of United Kingdom and Japanese respondents reporting different friendship group sizes (see Supporting Information S3, for FHQ scores for these groups).

**TABLE 4:** Study 1: Frequencies of friendship group sizes in the United Kingdom and Japan.

Typical friendship group	Japan	UK
1. Dyad	302	152
2. Group of three (2 friends)	107	117
3. Group of four (3 friends)	55	109
4. Group of five or more (4 + friends)	41	126

#### Cultural differences controlling for gender, age and socio‐economic status

To test the robustness of the cross‐country differences in friendship styles and practices, we conducted ANCOVAs controlling for three demographics: age, gender and socio‐economic status (SES). For friendship quantity, we additionally included friendship styles (FHQ scores) as a covariate. Detailed information about the models can be found in Supporting Information S7.

For friendship styles (FHQ scores), in addition to the expected effect of country with participants from the UK scoring higher than Japanese participants (*F*(1, 1026) = 68.41, *p* < .001, $\eta_{p}^{2}$ = .063), SES also had a significant effect with respondents higher in SES having more group‐oriented friendship styles, *F*(1, 1026) = 31.72, *p* < .001, $\eta_{p}^{2}$ = .034. Age and gender did not have significant effects (*F*(1, 1026) = 2.93, *p* = .087, $\eta_{p}^{2}$ = .003; *F*(1, 1026) = 1.72, *p* = .190, $\eta_{p}^{2}$ = .002, respectively). Since the homogeneity of variance assumption was violated for age, we further investigated the significant interaction between country and age, (*F*(1, 1026) = 4.29, *p* = .039, $\eta_{p}^{2}$ = .004). This showed that FHQ scores were consistent across different ages for Japan, but in the United Kingdom, younger participants had higher FHQ scores (Supporting Information Figure S3a).

When analysing proportion of time in groups, we observed significant effects of country (*F*(1, 1008) = 24.06, *p* < .001, $\eta_{p}^{2}$ = .023), age (*F*(1, 1008) = 10.11 *p* = .002, $\eta_{p}^{2}$ = .010), gender (*F*(1, 1008) = 9.43, *p* = .002, $\eta_{p}^{2}$ = .009) and SES (*F*(1, 1008) = 35.45, *p* < .001, $\eta_{p}^{2}$ = .034). Younger participants, men and those with higher SES spent more time in groups. Since the homogeneity of variance assumption was violated for age, we further investigated the significant interaction between country and age, (*F*(1, 1008) = 4.20, *p* = .041, $\eta_{p}^{2}$ = .004). This showed that the proportion of time spent in groups was consistent across different ages for Japan, but in the United Kingdom, younger participants spent more time in groups (Supporting Information Figure S3c).

For friendship group size, Japanese participants reported smaller groups than respondents from the United Kingdom (*F*(1, 1007) = 31.92, *p* < .001, $\eta_{p}^{2}$ = .031) and we observed significant effects of age (*F*(1, 1007) = 8.38, *p* = .004, $\eta_{p}^{2}$ = .008), gender (*F*(1, 1007) = 5.98, *p* = .015, $\eta_{p}^{2}$ = .006) and SES (*F*(1, 1007) = 9.24, *p* = .002, $\eta_{p}^{2}$ = .009). For age, similar to its effect on friendship styles, the ANCOVA revealed a significant interaction between country and age (*F*(1, 1007) = 4.73, *p* = .030, $\eta_{p}^{2}$ = .005), such that age was not related to friendship size in Japan but, in the United Kingdom, younger participants reported larger group sizes (Supporting Information Figure S3f). Across both countries, men and people with higher SES socialized in larger friendship groups.

In an exploratory ANCOVA examining the effects of country on the quantity of friends, the country difference remained the same *F*(1, 1025) = 26.34, *p* < .001, $\eta_{p}^{2}$ = .025 while gender, SES and age did not have significant effects (*F*(1, 1025) = 0.20, *p* = .658, $\eta_{p}^{2}$ = .000; *F*(1, 1025) = 0.42, *p* = .518, $\eta_{p}^{2}$ = .000; *F*(1, 1025) = 0.14, *p* = .711, $\eta_{p}^{2}$ = .000, respectively). In contrast, there was a significant effect of FHQ scores, *F*(1, 1025) = 15.76, *p* < .001, $\eta_{p}^{2}$ = .015, suggesting that higher FHQ scores are related to a larger quantity of friends. However, we observed a significant interaction between country and FHQ scores, *F*(1, 1025) = 12.47, *p* < .001, $\eta_{p}^{2}$ = .012. On further exploration, FHQ scores and quantity of friends were not related in Japan, but in the United Kingdom, people with higher FHQ scores reported having more friends (Supporting Information Figure S3k).

Overall, Japanese participants endorsed dyadic‐oriented friendship styles, spent less time in groups, had smaller group sizes and had fewer friends compared to UK participants.

#### Cultural differences in relational mobility and thinking styles

We tested whether Japanese participants would report less relational mobility and more holistic thinking than participants from the United Kingdom. The assumption of homogeneity of variance was violated for relational mobility, *F*(1, 1029) = 5.81, *p* = .016. Welch's *t*‐test showed that relational mobility was lower in the Japanese sample (*M* = 3.69, *SD* = .64) than in the UK sample (*M* = 4.00, *SD* = .69), *t*(1015.18) = −7.40, *p* < .001, *d* = −.46. The SEM supported this finding, −.23, *p* < .001.

For thinking styles, we used a Welch's *t*‐test as the assumption of homogeneity of variance was violated, *F*(1, 1032) = 7.56, *p* = .006. Japanese participants showed less holistic thinking (*M* = 4.71, *SD* = .41) than participants from the United Kingdom (*M* = 4.90, *SD* = .47), *t*(1008.96) = −6.81, *p* < .001, *d* = −.42. However, it is worth noting that the Analysis‐Holism Scale did not achieve configural measurement invariance and thus findings based on this scale might not be reliable.

### Discussion

Study 1 shows that more group‐oriented friendship styles, indicated by higher FHQ scores, are linked to more time spent in groups and larger sizes of friendship groups across the United Kingdom and Japan. Thus, the FHQ scale can measure real‐life interactions with friends, replicating earlier findings (Howlett et al., 2023). Here we extend this previous work by showing that FHQ scores predict socializing behaviours in two vastly different cultures, the United Kingdom and Japan.

Our findings demonstrate that, compared to Japanese, UK residents endorse more group‐oriented friendships. This result was consistent for both friendship styles and practices (FHQ scores, proportion of time in groups, size of friendship groups) and across two analytic techniques. It also remained significant when controlling for covariates. Moreover, group‐oriented friendship styles and practices were associated with higher relational mobility. As expected, participants from the United Kingdom reported higher relational mobility than participants from Japan. It is worth noting that existing studies on relational mobility link it with desire for self‐disclosure (Thomson et al., 2018), and the present study, as well as previous research (Howlett et al., 2023), suggest that high self‐disclosure with friends, as measured with the FHQ, is indicative of group‐oriented friendship styles. Self‐disclosure could intuitively be associated with dyadic friendships. However, our additional analyses (Supporting Information Tables S1c and S1d) highlight that self‐disclosure positively correlates with other indicators of group friendships and removing it from the model worsens the fit with FHQ.

Interestingly, FHQ scores were positively associated with friendship quantity in the UK, but not in Japan, and participants in the United Kingdom reported having more friends. Moreover, we found that group‐oriented friendship styles were associated with holistic thinking. Respondents from the United Kingdom reported more holistic thinking than Japanese respondents. This result, however, should be treated with caution, as the Analysis‐Holism Scale did not achieve measurement invariance, consistent with previous findings (Lacko et al., 2023).

Overall, Study 1 shows that the United Kingdom may promote group‐oriented friendship styles to a greater extent than Japan. Study 2 aimed to replicate this finding in a different sample with a smaller age gap between the two cultures.

## STUDY 2

Study 2 used a dataset collected for a different project (https://osf.io/gr42s/overview), including respondents from university students in Japan and the United Kingdom, making both samples more similar. We hypothesized that group‐oriented friendship styles, as measured by FHQ, would be related to a greater proportion of time in groups and larger friendship group sizes. We also expected higher levels of relational mobility in the United Kingdom than in Japan. Finally, we examined whether cultural differences in friendship styles and associations between relational mobility and friendship styles would replicate the findings of Study 1.

As an exploratory measure, Study 2 included a scale of anxious and avoidant attachment styles (Fraley et al., 2000), a construct potentially relevant to friendship behaviours. Adult attachment theory explains individual differences in how one thinks, feels and behaves in close relationships (Fraley et al., 2000). People high in avoidant traits are less relationship‐focused and evade closeness. Conversely, people high in anxious traits are more relationship‐focused and tend to worry about the stability and reciprocity of their bonds. Attachment styles have been linked with personality traits. For example, people reporting high anxious and avoidant attachment tend to report low extraversion and high neuroticism (Noftle & Shaver, 2006), with the latter potentially relevant to negative feelings towards friendship groups. Interestingly, cross‐cultural research on attachment shows that, while secure attachment is most common worldwide, respondents from East Asian countries, like Japan, score higher on anxious attachment whereas respondents from Western European and English‐speaking countries, like the United Kingdom, score higher on avoidant attachment (Schmitt et al., 2004). Since attachment influences how we interact with others, it can help explain cultural differences in friendship styles. We therefore explored the associations between FHQ scores and anxious and avoidant attachment, as well as the potential cultural differences in attachment.

### Methods

#### Participants

Participants in Japan (*N* = 312; age *M* = 23.43, *SD* = 4.60, range = 18–35; female = 159, other = 2) and in the United Kingdom (*N* = 317; age *M* = 19.61, *SD* = 1.71, range = 18–35; female = 240, other = 4) completed the study questionnaire and passed one attention check for a total sample of 629. The sample size rationale was the same as Study 1. Japanese respondents were recruited on the platform CrowdWorks (2021) with an advertisement targeting university students between 18 and 35 years old. They were paid 330 Yen for their help. UK participants were recruited at two university campuses and compensated with course credits.

Nearly all Japanese participants (*n* = 310, 99%) reported Japanese as their first language and most of the participants from the United Kingdom (*n* = 235, 74%) reported English. In the 2021 UK census, approximately 91% of the UK residents had English as a first or preferred language, so our sample slightly overrepresents people using other languages.

#### Procedure and materials

Methods were similar to the ones used in Study 1. Participants completed the Friendship Habits Questionnaire, the Relational Mobility Scale, and reported proportion of time in groups and the size of their friendship groups. The study included the short version of the Experiences in Close Relationships Scale (ECR‐S; Fraley et al., 2000) measuring anxious and avoidant attachment. The anxious attachment scale had excellent reliability (overall: *α* = .89, *ω* = .89; Japan: *α* = .88, *ω* = .89; UK: *α* = .90, *ω* = .90), as did the avoidant scale (overall: *α* = .85, *ω* = .86; Japan: *α* = .81, *ω* = .82; UK: *α* = .89, *ω* = .89). We did not measure thinking styles or quantity of friends. Respondents were included in the data analysis if they passed one attention check embedded in the FHQ.

### Results

#### Analytic strategy

The analytic approach was similar to the one used in Study 1. We controlled for the same covariates (see Supporting Information S8 and S10). The SEM model was identical to the one used in Study 1 and showed a borderline acceptable fit (Supporting Information S12). Additionally, the Experiences in Close Relationships Scale achieved partial scalar measurement invariance (Supporting Information S13).

#### Friendship styles and self‐reported friendship practices

We examined whether stronger group‐oriented friendship styles would be associated with a higher proportion of time spent in friendship groups and larger sizes of friendship groups. Correlation analyses (Tables 5 and 6) indeed revealed significant medium‐sized positive associations between these variables in the entire sample and within each of the cultures. The SEM replicated these findings (Table 7).

**TABLE 5:** Study 2: Descriptive statistics and spearman correlations between measures of friendship styles, friendship practices (more time spent in friendship groups and larger sizes of friendship groups), relational mobility, attachment styles and demographics (entire sample).

Variable	*n*	*M*	*SD*	1	2	3	4	5	6	7	8
1. FHQ	629	4.52	0.98	–							
2. Proportion of time in groups	624	50.49	25.73	.45***	–						
3. Friendship group size	624	2.42	1.55	.40***	.58***	–					
4. Relational mobility	623	4.06	0.67	.27***	.18***	.22***	–				
5. Anxious attachment	622	3.21	0.85	−.07	−.02	−.01	−.08	–			
6. Avoidant attachment	622	2.64	0.79	−.31***	−.14***	−.05	−.20***	.25***	–		
7. Age	629	21.50	3.95	−.28***	−.17***	−.30***	−.25***	−.05	.02	–	
8. Socio‐economic status	629	5.47	1.64	.29***	.12**	.09*	.12**	−.17***	−.14***	−.14***	–

* *p* < .05.

** *p* < .01.

*** *p* < .001.

**TABLE 6:** Study 2: Descriptive statistics and spearman correlations between measures of friendship styles, friendship practices (more time spent in friendship groups and larger sizes of friendship groups), relational mobility, attachment styles and demographics (Japanese and UK samples).

Variable	1	2	3	4	5	6	7	8
1. FHQ	–	.38***	.34***	.16**	−.07	−.21***	−0.02	.25***
2. Proportion of time in groups	.49***	–	.52***	.16**	−.01	−.05	0.00	.09
3. Friendship group size	.34***	.57***	–	0.08	.04	.02	−.15**	.06
4. Relational mobility	.19***	.13*	.23***	–	−.10	−.24 ***	−0.03	.09
5. Anxious attachment	−.09	−.06	−.09	−.09	–	.26***	−.03	−.25***
6. Avoidant attachment	−.42***	−.24***	−.13*	−.18**	.24***	–	−.02	−.11*
7. Age	−.16**	−0.1	−.16***	−.19***	−.06	.01	–	.05
8. Socio‐economic status	.21***	.08	.00	.04	−.09	−.16**	−.06	–
Japan								
*M*	4.17	45.77	1.98	3.89	3.19	2.66	23.43	5.06
*SD*	0.97	27.40	1.23	0.65	0.83	0.73	4.60	1.58
UK								
*M*	4.87***	55.12***	2.85***	4.24***	3.23	2.62	19.61***	5.87***
*SD*	0.85	23.10	1.71	0.65	0.87	0.84	1.71	1.61

*Note*: Numbers below the diagonal represent correlations in Japan, Numbers above the diagonal represent correlations in the United Kingdom. Asterisks denote significant pairwise comparisons.

* *p* < .05.

** *p* < .01.

*** *p* < .001.

**TABLE 7:** Study 2: Correlations (from SEM) between measures of friendship styles, friendship practices (more time spent in friendship groups and larger sizes of friendship groups), relational mobility and age (Japanese and UK samples).

Variable	1	2	3	4	5
1. FHQ	–	.42***	.34***	.24***	−.03
2. Proportion of time in groups	.54***	–	.46***	.17**	.01
3. Friendship group size	.34***	.53***	–	.08	−.11*
4. Relational mobility	.37***	.12	.17	–	−.04
5. Age	−.15**	−.04	−.11*	−.17*	–

*Note*: Numbers below the diagonal represent correlations in Japan; Numbers above the diagonal represent correlations in the United Kingdom.

* *p* < .05.

** *p* < .01.

*** *p* < .001.

#### Friendship styles, relational mobility and attachment styles

Consistent with Study 1, we found positive associations between FHQ scores, indicating group‐oriented friendship styles and relational mobility in the entire sample and within each culture (Tables 5 and 6). The SEM analysis replicated this result (Table 7). High FHQ scores were also negatively correlated with avoidant attachment. No significant associations were found between FHQ and anxious attachment (Tables 5 and 6).

#### Cultural differences in friendship styles and practices

FHQ scores were significantly lower for Japanese participants (*M* = 4.17, *SD* = .97) than participants from the United Kingdom (*M* = 4.87, *SD* = .85), Welch's *t*(613.00) = −9.65, *p* < .001, *d* = −.77, *F*(1, 627) = 4.67, *p* = .031. The SEM analysis replicated this result, with a significant intercept comparing Japan and the United Kingdom, −.55, *p* < .001. Therefore, consistent with Study 1, Japanese participants had more dyadic‐oriented friendship styles than those in the United Kingdom.

Cross‐cultural differences in friendship practices were consistent with those in friendship styles. Accordingly, Japanese participants spend less time in groups (*M* = 45.77%, *SD* = 27.40) than participants from the United Kingdom (*M* = 55.12%, *SD* = 23.10), Welch's *t*(600.74) = −4.61, *p* < .001, *d* = −.37, *F*(1, 622) = 4.67, *p* < .001, replicated by the SEM model −8.99, *p* < .001 and socialized in smaller groups (*M* = 1.98, *SD* = 1.23) than participants in the United Kingdom (*M* = 2.85, *SD* = 1.71), Welch's *t*(570.09) = −7.33, *p* < .001, *d* = −.59, *F*(1, 622) = 19.27, *p* < .001, replicated by the SEM model −.85, *p* < .001. Table 8 shows the breakdown of typical friendship groups for both countries (see Supporting Information S9, for FHQ scores for these groups).

**TABLE 8:** Study 2: Frequencies of friendship groups sizes in the United Kingdom and Japan.

Typical friendship group	Japan	UK
1. Dyad	143	68
2. Group of three (2 friends)	79	90
3. Group of four (3 friends)	48	69
4. Group of five or more (4 + friends)	36	88

#### Cultural differences controlling for gender, age and socio‐economic status

To test the robustness of the cross‐country differences in friendship styles and practices, we conducted ANCOVAs controlling for age, gender and SES using the same procedure as in Study 1 (Supporting Information S7 and S10).

The effect of country on FHQ scores remained significant, *F*(1, 618) = 42.32, *p* < .001, $\eta_{p}^{2}$ = .064, when controlling for covariates. Younger participants and people higher in socio‐economic status had more group‐oriented friendship styles, *F*(1, 618) = 5.17, *p* = .023, $\eta_{p}^{2}$ = .008, and *F*(1, 618) = 33.00, *p* < .001, $\eta_{p}^{2}$ = .051, respectively. Gender was not a significant covariate, *F*(1, 618) = 2.95, *p* = .086, $\eta_{p}^{2}$ = .005.

Similarly, the proportion of time spent in friendship groups remained significantly higher in the United Kingdom than in Japan when controlling for covariates, *F*(1, 613) = 13.12, *p* < .001, $\eta_{p}^{2}$ = .021. Men and respondents with higher socio‐economic status spent more time in groups, *F*(1, 613) = 9.24, *p* = .002, $\eta_{p}^{2}$ = .015, and *F*(1, 613) = 6.62, *p* = .010, $\eta_{p}^{2}$ = .011, respectively. Age had no significant effect, *F*(1, 613) = 0.61, *p* = .434, $\eta_{p}^{2}$ = .001.

The cross‐country difference in friendship group sizes remained significant after controlling for covariates, *F*(1, 613) = 27.84, *p* < .001, $\eta_{p}^{2}$ = .043. The effects of age, *F*(1, 613) = 5.98, *p* = .015, $\eta_{p}^{2}$ = .010, and gender, *F*(1, 613) = 4.63, *p* = .032, $\eta_{p}^{2}$ = .007, were also significant with younger respondents and men having larger friendship group sizes. There was no effect of socio‐economic status, *F*(1, 613) = 0.93, *p* = .336, $\eta_{p}^{2}$ = .002.

To summarize, consistent with Study 1, participants in the UK endorsed group‐oriented friendship styles more than Japanese participants. They also spent more time in groups rather than dyads and had larger friendship groups.

#### Cultural differences in relational mobility and attachment

In line with the findings of Study 1, Japanese participants were less relationally mobile (*M* = 3.89, *SD* = .65) than participants from the United Kingdom (*M* = 4.24, *SD* = .65), *t*(621) = −6.69, *p* < .001, *d* = −.54. This was replicated in the SEM analysis, −.28, *p* < .001.

An inspection of attachment styles in the United Kingdom and Japan revealed no significant cultural differences in avoidance (UK: *M* = 2.62, *SD* = 0.84, Japan: *M* = 2.66, *SD* = 0.73, Welch's *t*(608.87) = 0.54, *p* = .586, *d* = −.04) or anxiety (UK: *M* = 3.23, *SD* = .87, Japan: *M* = 3.19, *SD* = .83, *t*(620) = −0.56, *p* = .586, *d* = .04). This was replicated in the measurement invariance analysis, −.08, *p* = .321 and − .02, *p* = .804, respectively (Additional analyses in Supporting Information S11).

### Discussion

Study 2 replicated the findings of Study 1 and found that FHQ scores were positively associated with the proportion of time spent in groups and with the size of friendship groups. This effect was observed in both cultures, suggesting that the traits of extraversion, self‐disclosure, negative affect towards friendship groups and positive group identification reflect self‐reported real‐life behaviour. Participants in the United Kingdom had stronger group‐oriented friendship styles, spent a greater proportion of time in groups and had larger friendship groups than participants in Japan. Importantly, these differences occurred in a more homogenous sample than Study 1 and remained significant after controlling for demographic differences between samples. It also appears that male gender, being younger and having higher economic status are related to group‐oriented friendship styles and practices.

Participants from the United Kingdom reported more relational mobility than Japanese respondents, and higher relational mobility was associated with more group‐oriented friendship styles. We did not observe cross‐cultural differences in attachment. There was no relationship between anxiety and FHQ scores, but FHQ scores were negatively associated with avoidance. Therefore, considering avoidant attachment may help explain cultural differences in friendship styles.

Overall, these results provide further evidence that FHQ scores and relational mobility are positively associated and that the United Kingdom might promote more group‐oriented friendship styles than Japan. Moreover, avoidant attachment might be negatively related to FHQ scores.

## GENERAL DISCUSSION

### Summary of findings

People differ in whether they socialize with one or multiple friends. However, little is known about how our friendship styles relate to other social constructs such as relational mobility, describing the degree to which people in a society can freely form new relationships and leave old ones (Thomson et al., 2018), thinking styles, potentially influencing group perception (e.g. Masuda et al., 2008) or attachment styles (Schmitt et al., 2004). Friendship styles and practices also differ culturally, but the mechanisms underlying these differences are yet to be understood. This research explored the relationships between group‐oriented friendship styles, self‐reported friendship practices, relational mobility and holistic thinking in the United Kingdom and in Japan. We predicted that FHQ, a new measure of group‐ versus dyadic‐oriented friendship styles, would be reliably associated with friendship practices. We also compared friendship styles and levels of relational mobility, as well as holistic thinking and attachment between respondents from Japan and the United Kingdom.

We found that group‐oriented friendship styles, as indicated by higher FHQ scores, were positively linked with the proportion of time spent in friendship groups and with the size of such groups. Moreover, respondents from the United Kingdom had higher FHQ scores, spent more time in groups and had larger friendship groups. Relational mobility was positively related to group‐oriented friendship styles; participants from the United Kingdom were more relationally mobile than participants from Japan.

The positive associations between friendship styles (FHQ scores) and friendship practices (proportion of time in groups and size of friendship groups) were observed in both countries, strengthening and extending previous findings on the FHQ (Howlett et al., 2023). Across two vastly different cultures, respondents high in extraversion, need for self‐disclosure, and positive affect towards friendship groups also tend to spend more time in friendship groups and have larger friendship groups. Beyond showing that the FHQ is a robust indicator of friendship practices, suitable for cross‐cultural research, the present study points to the discriminant validity of this scale.

In Study 1, our exploratory analysis of quantity of friends highlights how group‐oriented friendship style, measured via FHQ, is distinct from friendship quantity. Although group‐oriented friendship style was moderately correlated with the proportion of time spent in friendship groups and with friendship group size, it was only weakly correlated with the number of friends, and this last correlation was not cross‐culturally stable. In other words, two people may each have six friends, but one might socialize in a big group, while the other may meet each of the six friends individually. Study 2 included a measure of attachment styles, a construct relevant to social relationships and friendships. We found that FHQ scores were negatively associated with avoidant, but not anxious attachment. Moreover, attachment patterns (unlike friendship styles and practices) were similar across the United Kingdom and Japan. Together, these results suggest meaningful differences among friendship styles, attachment and overall friendship competence.

### Friendship styles and relational mobility

Friendship styles also appear to be distinct from relational mobility. In the present studies, the two constructs were positively correlated. Supporting previous research (Thomson et al., 2018), respondents from the United Kingdom scored higher on relational mobility than respondents from Japan. As relational mobility is associated with increased opportunities to make friends and change social groups, it might be that relationally mobile environments promote the formation of larger friendship groups. Our results support such an interpretation by showing that participants from the United Kingdom spend more time in group, rather than dyadic, interactions and overall socialize in larger groups than Japanese participants. To further investigate friendship styles and relational mobility, we conducted two exploratory ANCOVA analyses (for Study 1 and Study 2), examining cross‐country differences in relational mobility and controlling for FHQ scores, age, gender and socio‐economic status.^1^ Interestingly, in Study 1, the cross‐cultural difference in relational mobility was no longer significant after controlling for FHQ scores and socio‐economic status. FHQ scores remained a significant predictor of relational mobility across both studies, indicating that variations in relational mobility are at least partly driven by friendship styles.

### Culture and group friendships

Importantly, respondents from the United Kingdom had higher FHQ scores, spent more time in groups and had larger friendship groups. This was found in two studies, across two analytic techniques, and when controlling for covariates of age, gender and socio‐economic status. This finding might at first seem counterintuitive, as Japan is often described as a collectivist society (e.g. Hofstede, 1980; Hofstede et al., 2010), potentially promoting group‐oriented friendship styles. However, recent evidence highlights demographic changes in these societies, which might potentially explain the observed cross‐cultural differences in friendship styles. For example, in 2015, the percentage of single‐person households was 34.53% in Japan and 28.70% in the United Kingdom (Eurostat, 2015; Ortiz‐Ospina, 2019). However, in 2005, the percentage of single households was lower in Japan than in the United Kingdom (Eurostat, 2015; Ortiz‐Ospina, 2019), which might be why studies published before this date suggested that Japan promotes group friendships (e.g. Cargile, 1998; White, 1993). Furthermore, living in single‐person households is associated with social isolation, a link particularly strong in Japan (Hill et al., 2009). Thus, the rise in living alone might have led to a reduction in friendship groups in Japan. Alternatively, the Ease of Settling in Index, which measures social life satisfaction and ease of making friends among immigrants in their host country, ranks the United Kingdom and Japan 42nd and 55th, respectively (InterNations, 2021). Although this finding refers to non‐native citizens, it hints that expanding the size of friendship groups may be more challenging in Japan than in the United Kingdom. Respondents from the United Kingdom might also enjoy socializing in groups. For example, a social network study by Pearson et al. (2006) in the United Kingdom found that, based on friendship nominations in their school classes, 25% of children were part of large groups of over 6 people, 30% in small groups of between 3 and 5 people, and 32% were involved in various forms of dyadic friendships (including children connected to one member of an overall group). These findings hint to considerable variation in friendship styles within the United Kingdom, but, to our knowledge, no similar study was conducted in Japan or other Eastern cultures.

A review of research examining the dimensions measured in the FHQ might provide further insights to why Japanese respondents endorsed more dyadic‐oriented friendship styles in the present research. Specifically, people from East Asian countries tend to be less extraverted and higher in neuroticism than Europeans (e.g. Allik et al., 2017; Schmitt et al., 2007). In addition, Japanese disclose less personal information in their relationships than Westerners (e.g. Kito, 2005). Finally, compared to Australians, Japanese people identify less strongly with friendship groups (Kashima & Hitokoto, 2009). Such and similar results suggest that Japanese people are higher in personality traits relevant to dyadic‐oriented friendships.

### Implications for collectivism–individualism

Our findings about friendship styles and practices in Japan and in the United Kingdom appear to challenge the “common view” of collectivism–individualism. According to this perspective, Eastern societies are more collectivistic and more group‐oriented, whereas the opposite is true for Western societies (Matsumoto, 2018, see also Vignoles et al., 2016). The present research adds further nuance to the predictive validity of collectivism–individualism and relational mobility in cultural research on friendship. Low relationally mobile societies offer fewer opportunities to choose friends (e.g. Thomson et al., 2018), suggesting that friendships in collectivist countries like Japan are more likely to be viewed as pre‐determined (Hofstede, 1980). In line with previous research (Thomson et al., 2018), we show that relational mobility is higher in the United Kingdom than Japan, yet it is the United Kingdom participants who endorse more group‐oriented friendship styles. This finding dovetails with the results of an earlier diary study (Kafetsios, 2006), where participants from the United Kingdom reported much more frequent interactions with friends and best friends than participants from Greece, a relatively collectivistic culture (e.g. Hofstede et al., 2010). It is possible that, in more collectivistic and less relationally mobile cultures, less emphasis is put on maintaining relationships, especially with non‐kin (Kafetsios & Nezlek, 2012; Schug et al., 2010). Moreover, collectivistic cultures might vary in the extent to which they promote group versus dyadic friendships. For example, French et al. (2006) collected data from university students in two cultures viewed as collectivist, South Korea and Indonesia, and the USA, viewed as individualistic. The researchers observed the largest friendship groups among Indonesian participants and the smallest friendship groups in South Korea, with the USA falling in‐between. The USA is similar to the United Kingdom in relational mobility (Thomson et al., 2018) and, unlike Indonesia, both South Korea and Japan are cultures influenced by Confucianism. In Confucian philosophy, friendships involving trust, acceptance, and intimacy are important for society (Wei‐Ming, 1996). The emphasis on close friendships in Confucian cultures could help explain the small friendship groups observed in South Korea by French et al. (2006) and the findings of the present study. In addition, and contrary to classic accounts of individualism–collectivism (Hofstede, 1980), a recent revised model of these dimensions (Minkov & Kaasa, 2022) suggests that, in the 2010s, Japan was an individualistic country. It is therefore possible that our research compares two individualistic countries, documenting the limitations of individualism–collectivism in explaining cultural differences in friendship styles. Importantly, our study only included two cultures. Examining friendship styles in a greater number of countries could provide a nuanced understanding of the explanatory power of relational mobility, collectivism–individualism and other cultural dimensions in the study of friendship styles.

### Implications for thinking styles

As predicted, we observed positive associations between group‐oriented friendship styles and holistic thinking. However, the UK participants scored significantly higher on holistic thinking than the Japanese participants. Our results may seem surprising in the light of past research showing more analytic thinking in the United Kingdom and more holistic thinking in Japan (Kitayama et al., 2009), but it is important to take this finding with caution. The AHS scale, measuring thinking styles (Choi et al., 2007), did not achieve measurement invariance and its structure has also not been replicated in other studies (Lacko et al., 2023), suggesting people from different countries may interpret analytic and holistic thinking differently. Furthermore, AHS is a self‐report measure and analysis‐holism might be better assessed with behavioural measures, such as gaze patterns or evaluations of visual scenes (e.g. Masuda et al., 2008), rather than self‐reports (Lacko et al., 2023, 2025). Participants could, for example, rate the intensity of emotion experienced by an individual in a group (e.g. Masuda et al., 2008). In such tasks, analytic thinkers should mainly focus on the individual of interest whereas holistic thinkers would consider the whole group when making their judgements (e.g. Hess et al., 2016; Nisbett et al., 2001). Therefore, future studies of friendship styles could utilize visual attention tasks, as a person's attention to facial expressions in groups of people might be more relevant to friendship styles than their self‐reports.

### Limitations and next steps

It is worth noting that samples from Japan and the United Kingdom differed in age, gender and socio‐economic status. Although controlling for all covariates did not affect the key findings, it would be valuable for future research to examine more homogenous samples, minimizing potential differences in friendships due to age, gender and social position. It is also interesting to explore how friendship group size changes throughout the lifespan. Importantly, all measures in the present research are self‐reported. Although measuring a person's typical friendship group is a difficult task and observational studies are often impractical, implementing daily diary studies (e.g. French et al., 2006) would allow more ecologically valid insights into social interactions. Given that such studies are not always possible, quick measures like the FHQ allow for time‐ and cost‐effective measurement of friendship styles, complementing and informing more intensive assessments of interactions with friends. While initial research suggests the FHQ is reliable, valid and suitable for use in different cultures, the very construct of group‐ versus dyadic‐oriented friendship styles needs further investigation, both with regard to its exact meaning and nomological network. Future studies should examine relationships between FHQ scores and quality of friends (Pezirkianidis et al., 2023), as well as other individual differences, including generalized trust, relationism (Igarashi et al., 2008) and self‐esteem. Ideally, future studies would also compare FHQ scores with socializing with friends captured using diaries or other experience sampling methods.

We believe future research on friendship can benefit from considering the role of friendship styles or individual tendencies to socialize in friendship groups versus dyads. Researchers should avoid assuming that everyone socializes or wants to socialize in groups. Conversely, conceptualizing friendship as a purely dyadic bond should also be avoided as it overlooks the complexity of friendships in different cultures and does not reflect everyone's reality. The results of the present study and past research (e.g. French et al., 2006; Peperkoorn et al., 2020) highlight that friendship groups differ across individuals and cultures. Humans clearly socialize in dyads as well as groups (Dunbar, 2018) and it is important for future research to investigate the factors that lead people to become more group‐ or dyadic‐oriented and to explore potential impacts of such differences on our everyday life.

## AUTHOR CONTRIBUTIONS

**Philip Howlett:** Conceptualization; methodology; investigation; validation; writing – original draft; writing – review and editing; formal analysis; project administration; data curation. **Gülseli Baysu:** Supervision; writing – review and editing; formal analysis. **Tomas Jungert:** Conceptualization; writing – review and editing; supervision. **Anthony P. Atkinson:** Writing – review and editing; supervision. **Shushi Namba:** Investigation; data curation; methodology; writing – review and editing. **Wataru Sato:** Data curation; methodology; investigation; writing – review and editing. **Kumpei Mizuno:** Methodology; investigation; data curation; writing – review and editing. **Magdalena Rychlowska:** Writing – review and editing; writing – original draft; supervision.

## FUNDING INFORMATION

Author PH was awarded a funded Studentship from the Department for the Economy, Northern Ireland (https://www.economy‐ni.gov.uk/). The funders had no role in study design, data collection and analysis, decision to publish, or preparation of the manuscript.

## CONFLICT OF INTEREST STATEMENT

The authors declare no conflict of interest.

## ETHICS STATEMENT

This research has obtained approvals from the ethics committees at the Faculty of Engineering and Physical Sciences (EPS), Queen's University Belfast, UK, and Hokkaido University of Education, Japan. These approvals indicate that the research protocol met ethical guidelines in the United Kingdom and Japan.

## ANALYSIS, DATA & MATERIAL ACCESSIBILITY STATEMENT

Study 1 was pre‐registered (preregistration available at: https://osf.io/cwz3x/?view_only=128ffac56bb34bcbb1ac78bfa138c046). Study materials, relevant data, and analysis codes are available on Open Science Framework at: https://osf.io/hfma6/?view_only=e8dca8493f5042458c1e2025f36d1b33.

## Supporting information


**Data S1:** Supplementary Information.

## Data Availability

The data that support the findings of this study are openly available in Open Science Framework at https://doi.org/10.17605/OSF.IO/HFMA6.

## Footnotes

^1^
Study 1: The between‐country difference in relational mobility was no longer significant, *F*(1, 1012) = 3.47, *p* = .063, $\eta_{p}^{2}$ = .003, when controlling for FHQ scores, age, gender, and SES. FHQ scores, *F*(1, 1012) = 61.62, *p* < .001, $\eta_{p}^{2}$ = .057, and socioeconomic status, *F*(1, 1012) = 4.25, *p* = .039, $\eta_{p}^{2}$ = .004, were significant covariates whereas age and gender were not, *F*(1, 1012) = 0.54, *p* = .462, $\eta_{p}^{2}$ = .001 and *F*(1, 1012) = 1.81, *p* = .179, $\eta_{p}^{2}$ = .002, respectively. Higher FHQ scores and SES predicted higher relational mobility. Study 2: The cross‐cultural difference in relational mobility remained significant, *F*(1, 611) = 4.79, *p* = .029, $\eta_{p}^{2}$ = .008, when controlling for covariates. FHQ scores, *F*(1, 611) = 22.30, *p* < .001, $\eta_{p}^{2}$ = .035, age, *F*(1, 611) = 4.16, *p* = .042, $\eta_{p}^{2}$ = .007, and gender, *F*(1, 611) = 9.40, *p* = .002, $\eta_{p}^{2}$ = .015, were all significant covariates. Higher FHQ scores, younger age, and male gender predicted higher relational mobility. Socioeconomic status was not a significant covariate, *F*(1, 611) = 0.50, *p* = .480, $\eta_{p}^{2}$ = .001.
