# Supplementary material for: Friendships are more group‐oriented in the United Kingdom than in Japan
Source: Br J Soc Psychol. 2026 Jan 16;65(1):e70040. doi: 10.1111/bjso.70040 (PMC12811512; doi:10.1111/bjso.70040)
Supplement: Supplementary file 1 — Data S1: Supplementary Information. [file BJSO-65-0-s001.docx]

**Friendships Are More Group-oriented in the UK Than in Japan**

**Supporting Information**

**Materials, Methods and Results**

**S1. Study 1: Demographic Testing**

**S2. Study 1: Measurement Invariance for the Friendship Habits Questionnaire**

**S3. Study 1: Self-reported Friendship Group Sizes and FHQ Scores**

**S4. Study 1: Measurement Invariance for the Relational Mobility Scale**

**S5. Study 1: Measurement Invariance for the Analysis-Holism Scale**

**S6. Study 1: Alternative Hypothesis Testing with the Combined Friendship Habits and Relational Mobility Models**

**S7. Study 1: ANCOVA Interaction Graphs and Assumption Testing**

**S8. Study 2: Demographic Testing**

**S9. Study 2: Self-reported Friendship Group Sizes and FHQ Scores**

**S10. Study 2: ANCOVA Interaction Graphs and Assumption Testing**

**S11. Study 2: Attachment Styles: Supplementary Analyses**

**S12. Study 2: Alternative Hypothesis Testing with the Combined Friendship Habits and Relational Mobility Models**

**S13. Study 2: Measurement Invariance for Experiences in Close Relationships Scale**

**S1. Study 1: Demographic Testing**

The number of female vs. male respondents was significantly different across samples, χ² (1, *N* = 1037) = 34.37, *p* <.001 and friendship styles have been shown to vary depending on gender (Benenson, 2019). However, in the present study women and men did not significantly differ in their FHQ scores, *t*(1030) = 1.87, *p* = .062, or the two other measures of socializing behaviors: proportion of time in groups, *t*(1012) = -.21, *p* = .834, or friendship group size, *t*(1011) = -.13, *p* = .894.

Japanese participants rated themselves as lower on socioeconomic status (*M* = 4.71, *SD* = 1.60) than participants from the UK (*M* = 5.58, *SD* = 1.54), *t*(1043) = -9.01, *p* < .001, *d* = -.56.

There was a statistically significant 7-year age gap between the UK and Japanese sample, Welch’s t-test *t*(1036.66) = 29.66, *p* < .001, *d* = 1.83.

**S2. Study 1: Measurement Invariance for the Friendship Habits Questionnaire**

The 23 items of the Friendship Habits Questionnaire (FHQ; Howlett et al., 2023) are presented in Table S1a under the relevant dimension names (e.g., Extraversion). Item codes (e.g., E1, E2) will be consistently used in the Supplementary Materials. All 23 items underwent measurement invariance testing (Howlett et al., 2023) and were retained for further analyses.

**Table S1a**

*Items and Dimensions of the Friendship Habits Questionnaire (Howlett et al., 2023)*

| Extraversion  E1 I am outgoing and sociable when I am in larger groups of friends |
| --- |
| E2 I am talkative when I am in larger groups of friends |
| E3 I am reserved when I am in larger groups of friends* |
| E4 I am full of energy when I am in larger groups of friends |
| E5 I tend to be quiet when I am in larger groups of friends* |
| E6 I have an assertive personality |
| E7 I am sometimes shy and inhibited in larger groups of friends* |
| E8 I generate a lot of enthusiasm when I am in larger groups of friends |
| Intimacy |
| I1 My friends and I always tell each other our problems |
| I2 My friends and I talk about the things that make us sad |
| I3 I tell my friends when I am mad about something that happened to me |
| I4 My friends and I tell each other secrets |
| I5 My friends and I tell each other private things |
| I6 My friends and I talk about how to make ourselves feel better if we are mad at each other |
| Positive Group Identification |
| GP1 I am glad when I belong to a friendship group |
| GP2 I identify with a friendship group |
| GP3 I feel strong ties to a friendship group |
| GP4 I think friendship groups work well together |
| GP5 I see myself as an important part of a friendship group |
| Negative Group Identification |
| GN1 I feel held back in friendship groups* |
| GN2 I do not consider a friendship group to be important* |
| GN3 I do not fit in well with other members of friendship groups* |
| GN4 I feel uneasy with members of friendship groups* |

**Reverse-scored items*

We tested cross-cultural measurement invariance of the Friendship Habits Questionnaire (FHQ) to examine if the questionnaire was interpreted similarly in both the UK and Japan. We aimed to achieve at least partial scalar invariance, a level of measurement invariance allowing to compare mean differences in FHQ scores between the two cultures. To reach this, a questionnaire must first achieve configural invariance. This means that the scale has the same factor structure in different samples (Putnick & Bornstein, 2016). The second, intermediate level of measurement invariance, is metric invariance. Achieving this level means that a questionnaire has equal factor loadings across different samples (Putnick & Bornstein. 2016). Finally, scalar invariance means that specific questionnaire items have equal intercepts (or: indicators of means) across different samples. When achieving complete scalar invariance is not possible, partial scalar invariance allows some intercepts to vary (Putnick & Bornstein, 2016) and allows researchers to compare participants’ responses across cultures.

Although chi-square difference is often used to examine measurement invariance, this measure is also sensitive to large sample sizes such as ours (e.g., French & Finch, 2008). Therefore, we chose to use the goodness-of-fit indices aiming for an acceptable fit (CFI > .90, RMSEA < .08, Tarling, 2008, and SRMR < .08, Hu & Bentler, 1999) at each stage of invariance. Once these standards were met at each level of invariance, we used Chen’s (2007) criteria for CFI, RMSEA and SRMR differences for comparing models at each level (e.g., configural vs. metric). Between each stage of measurement invariance, the model cannot have a CFI difference decrease of .010 or more, a RMSEA increase of more than .015 and an SRMR increase of more than .030. If at any point these differences were violated, we used a data-driven approach to free up parameters of the model until invariance was achieved at the specified stage.

Firstly, we estimated a four-factor model of group- versus dyadic-oriented friendship styles including extraversion, intimacy, positive group identification and negative group identification (Howlett et al., 2023). These four factors were estimated as first-order dimensions, with the corresponding FHQ items used as indicators. All four first-order factors loaded onto a second-order factor of friendship styles.

Due to the cultural comparisons, we also examined an alternative model including response styles. People from different countries tend to have different response styles (e.g., Fischer et al., 2009), for example consistently choosing the extreme ends of the scale (extreme response style) or the center of the scale, such as the option *agree* (acquiescent response style, e.g., Billiet and McClendon, 2000; Fischer et al., 2009; Johnson et al., 2011). It is therefore recommended for measurement invariance testing to include a common bias factor to control for participants’ response styles (Cheung & Rensvold, 2000). Here we followed the approach of Thomson and colleagues (2018). We added and modelled a common bias factor. All 23 FHQ items were allowed to load onto a factor of response style. Each scale item was multiplied by +1 and each reverse-scored item was multiplied by -1. The response style factor was then set not to correlate with the other factors, in this case friendship styles.

We first tested the original 4-factor model and then the same model with the addition of a common bias factor reflecting response style. The initial analyses were conducted using the entire dataset including both cultures. The data had high kurtosis and skewness as estimated with a Mardia’s test. We therefore used the maximum likelihood estimator with robust standard errors [MLR] and provide robust indices for χ², CFI, TLI and RMSEA. The 4-factor model without a common bias factor did not have an acceptable fit, as reflected in the CFI and TLI values (see Table S1b). When looking at the chi-square separately for the two countries, it appears that the model fit was less acceptable in Japan, χ² = 1128.00, than in the UK, χ² = 772.64. The 4-factor model with a common bias factor controlling for response style, improved the model fit across all indices, such that each index reached an acceptable level. However, adding the common bias factor caused negative variance for negative group identification in Japan. As the model was identified, outliers were removed (see main text), which resulted in an improved model fit. We allowed the variance of group identification to be set at 0 following the recommendations by Chen and colleagues (2001). Table S1b presents model fit indices for the model with variance set to 0 for negative group identification. When looking at the chi-square separately for the two countries, it appears that the model fit for Japan was less good , χ² = 862.38, than the fit of the UK model, χ² = 656.77.

**Table S1b**

*Robust Model Fit Indices for the Four-Factor Model of Friendship Styles (Extraversion, Intimacy, Positive Group Identification, Negative Group Identification) and Three-Factor Model of Friendship Styles (Extraversion, Positive Group Identification, Negative Group Identification) Both Excluding and Including a Common Bias Factor*

| Model | χ² | df | CFI | TLI | SRMR | RMSEA | RMSEA 90% CI | |
| --- | --- | --- | --- | --- | --- | --- | --- | --- |
| 4-factor model | 1900.64^***^ | 452 | .883 | .869 | .076 | .086 | (.082, .090) | |
| 3-factor model | 2113.56^***^ | 454 | .865 | .850 | .170 | .092 | (.088, .096) | |
| 4-factor model (with common bias) | 1519.14^***^ | 452 | .914 | .904 | .067 | .067 | (.064, .071) | |
| 3-factor model (with common bias) | 1755.28^***^ | 454 | .896 | .884 | .165 | .081 | (.077, .085) | |
| *^***^p < .001.* | | | | | | | |  |

**Self-Disclosure**

The inclusion of self-disclosure in the model as a group-oriented trait may seem counterintuitive as self-disclosure has been conceptualized in past literature as a major component of close, dyadic friendship (e.g., Bukowski et al., 1994; Mendelson & Aboud, 1999). Although Howlett et al. (2023) predicted self-disclosure should be dyadic-oriented, they found self-disclosure positively correlates with extraversion, positive group identification, and positive affect in groups (when reverse scoring negative group identification) suggesting that self-disclosure is more relevant to group- rather than dyadic-oriented friendships. In the present study, we also found that self-disclosure correlated positively with group-oriented traits in both Study 1 (Table S1c) and Study 2 (Table S1d).

Unlike Howlett et al. (2023), we therefore explored whether removing the factor of self-disclosure from the Friendship Habits Questionnaire could improve the model fit of the scale. To this end, we compared two 4-factor models (including Extraversion, Intimacy, Positive Group Identification, Negative Group Identification, with and without common bias) with two 3-factor models excluding self-disclosure (one with common bias, one without it). An inspection of the fit indicators of both 3-factor models showed a decrease of fit in a range of difference criteria (Chen, 2007; Table S1b), suggesting that self-disclosure is a meaningful component of group-oriented friendship styles and should remain included in the FHQ.

Higher levels of self-disclosure were associated with more group-oriented (rather than dyadic-oriented) friendship styles. This can be because self-disclosure reflects closeness which is important for friendships in general (e.g., Kaufman et al., 2022), not just dyadic friendships. Importantly, little is known about self-disclosure in friendship groups as most existing studies on this construct involved groups of strangers (Cooney et al., 2023). This previous research suggests that self-disclosure among strangers decreases in bigger groups (Solano & Dunnam, 1985), but such effects have not been examined in groups of friends. Such studies are an important step for future research as people indicate they are willing to share secrets in groups based on communal sharing, where relationships are based on shared identity (Fiske 1992; Travaglino et al., 2023). Importantly, the construct of close friends is closely related to communal sharing (Zakharin & Bates, 2023) and it is reasonable to assume that a group of friends who feel comfortable with each other and as a group are more likely to self-disclose than a group of strangers. Given its positive associations with other traits indicating group-oriented friendship styles, self-disclosure might facilitate maintaining the structure of friendship groups – a question that deserves to be explored in future research.

**Table S1c**

*Study 1: Spearman Correlations Between Factors of Friendship Styles (Extraversion, Self-Disclosure, Negative Group Identification, Positive Group Identification) in Japanese and UK Samples. Numbers Below the Diagonal Represent Correlations in Japan, Numbers Above the Diagonal Represent Correlations in the UK.*

|  | 1 | 2 | 3 | 4 |
| --- | --- | --- | --- | --- |
| 1. Extraversion | - | .38^***^ | -.44^***^ | .47^***^ |
| 2. Self-Disclosure | .40^***^ | - | -.40^***^ | .50^***^ |
| 3. Negative Group Identification | -.64^***^ | -.35^***^ | - | -.68^***^ |
| 4. Positive Group Identification | .54^***^ | .51^***^ | -.66^***^ | - |

**Table S1d**

*Study 2: Spearman Correlations Between Factors of Friendship Styles (Extraversion, Self-Disclosure, Negative Group Identification, Positive Group Identification) in Japanese and UK Samples. Numbers Below the Diagonal Represent Correlations in Japan, Numbers Above the Diagonal Represent Correlations in the UK.*

|  | 1 | 2 | 3 | 4 |
| --- | --- | --- | --- | --- |
| 1. Extraversion | - | .22^***^ | -.38^***^ | .43^***^ |
| 2. Self-Disclosure | .40^***^ | - | -.37^***^ | .49^***^ |
| 3. Negative Group Identification | -.55^***^ | -.31^***^ | - | -.62^***^ |
| 4. Positive Group Identification | .56^***^ | .57^***^ | -.58^***^ | - |

**Cross-Cultural Models**

We also estimated the fit of two 4-factor models (Extraversion, Intimacy, Positive Group Identification, Negative Group Identification, with and without common bias) separately for each country. We therefore ran two second-order CFAs and estimated the model fit for the UK and Japan. As seen in Table S1e, the UK model had an acceptable fit on all indices and the Japanese model achieved an acceptable fit on all indices but TLI. Overall, although the model reached a better fit in the UK than in Japan, the present analysis suggests that the 4-factor model of friendship styles (Howlett et al., 2023) including a common bias factor had a good fit in both countries, thus reaching configural measurement invariance (see Table S1f for factor loadings and Table S1g for a summary of the measurement invariance analysis).

**Table S1e**

*Robust Model Fit Indices for the Four-Factor Model of Friendship Styles (Extraversion, Intimacy, Positive Group Identification, Negative Group Identification), with a Common Bias Factor, for Japan and the UK*

| Model | χ² | df | CFI | TLI | SRMR | RMSEA | RMSEA 90% CI | |
| --- | --- | --- | --- | --- | --- | --- | --- | --- |
| Japan | 810.93^***^ | 226 | .902 | .890 | .077 | .079 | (.073, .085) | |
| UK | 701.26^***^ | 226 | .927 | .918 | .062 | .067 | (.062, .073) | |
| *^***^p < .001.* | | | | | | | |  |

**Table S1f**

*Study 1: Factor Loadings for the Four-Factor Model of Friendship Styles (Extraversion, Enjoyment of Intimacy, Positive Group Identification, Negative Group Identification) with a Common Bias Factor at the Configural Level of Measurement Invariance for the UK and Japan*

| Factor | Item | UK | | Japan | |
| --- | --- | --- | --- | --- | --- |
|  |  | B (SE) | β | B (SE) | β |
| Extraversion | E1 | 1.00 | .88 | 1.00 | .80 |
|  | E2 | 1.00^***^ (.03) | .90 | 1.05^***^ (.04) | .84 |
|  | E3 | .93^***^ (.04) | .85 | 1.05^***^ (.04) | .85 |
|  | E4 | .88^***^ (.03) | .84 | .92^***^ (.04) | .77 |
|  | E5 | .94^***^ (.04) | .85 | 1.11^***^ (.04) | .87 |
|  | E6 | .49^***^ (.04) | .46 | .46^***^ (.06) | .37 |
|  | E7 | .81^***^ (.04) | .79 | 1.13^***^ (.04) | .88 |
|  | E8 | .84^***^ (.03) | .83 | .96^***^ (.04) | .78 |
| Intimacy | I1 | 1.00 | .75 | 1.00 | .68 |
|  | I2 | .84^***^ (.05) | .66 | .94^***^ (.07) | .64 |
|  | I3 | .75^***^ (.05) | .60 | .92^***^ (.08) | .59 |
|  | I4 | .89^***^ (.06) | .77 | 1.13^***^ (.09) | .76 |
|  | I5 | .90^***^ (.06) | .81 | .93^**^ (.08) | .70 |
|  | I6 | .78^***^ (.06) | .53 | .62^**^ (.07) | .43 |
| Positive Group Identification | GP1 | 1.00 | .54 | 1.00 | .59 |
|  | GP2 | 2.18^***^ (.23) | .83 | 1.20^***^ (.09) | .74 |
|  | GP3 | 2.39^***^ (.25) | .89 | 1.44^***^ (.10) | .82 |
|  | GP4 | 1.21^***^ (.13) | .61 | 1.24^***^ (.10) | .75 |
|  | GP5 | 1.86^***^ (.22) | .72 | 1.26^***^ (.11) | .71 |
| Negative Group Identification | GN1 | 1.00 | .61 | 1.00 | .81 |
|  | GN2 | .90^***^ (.11) | .54 | .64^***^ (.10) | .48 |
|  | GN3 | 1.12^***^ (.10) | .67 | .76^***^ (.09) | .70 |
|  | GN4 | 1.06^***^ (.09) | .66 | .71^***^ (.08) | .61 |
| Friendship Styles | Extra | 1.00 | .57 | 1.00 | .84 |
|  | Intimacy | .70^***^ (.08) | .58 | .59^***^ (.08) | .61 |
|  | Positive Group I | .61^***^  (.08) | .87 | .69^***^ (.09) | .86 |
|  | Negative Group I | 1.01^***^ (.10) | 1.00 | 1.26^***^ (.05) | 1.00 |

**Table S1g**

*The Measurement Invariance Testing Process for the Four-Factor Model of Friendship Styles* *with a Common Bias Factor. Table Shows Robust Model Fit Indices at Different Levels of Measurement Invariance*

| Model | | χ²(df) | Δχ²(df) | CFI | TLI | SRMR | RMSEA | | RMSEA 90% CI | Decision |  |
| --- | --- | --- | --- | --- | --- | --- | --- | --- | --- | --- | --- |
| Configural | | 1519.14(452)^***^ |  | .914 | .904 | .067 | .073 | (.069, .077) | | Accept |  |
| Metric | | 1663.41(474)^***^ | 146.50(22)^***^ | .905 | .898 | .078 | .076 | (.072, .080) | | Reject |  |
| Partial Metric (GP4 factor loading varied) | | 1627.88(473) ^***^ | 110.28(21)^***^ | .907 | .901 | .076 | .075 | (.071, .079) | | Accept |  |
| Scalar (GP4 factor loading varied) | | 2129.40(490)^***^ | 551.43(17) ^***^ | .869 | .865 | .092 | .087 | (.083, .091) | | Reject |  |
| Partial Scalar (GP4 factor loading varied; E7, I5, GP1, GN1 & GN2 intercept varied) | | 1722.20(483)^***^ | 97.71(12) ^***^ | .901 | .896 | .080 | .076 | (.072 .080) | | Accept |  |
|  | *^***^p < .001.* | | | | | | | | | | |

While the 4-factor model with a common bias factor achieved configural invariance (see Table S1f and S1g), it failed to achieve metric invariance due to a borderline failure of CFI difference (= .01; see Table S1h for factor loadings, Chen, 2007). This model was rejected, and we inspected the modification indices to examine if there was a factor loading that should differ between groups.

**Table S1h**

*Factor Loadings for the Four-Factor Model of Friendship Styles (Extraversion, Enjoyment of Intimacy, Positive Group Identification, Negative Group Identification) with a Common Bias Factor at Metric Level of Measurement Invariance for the UK and Japan*

| Factor | Item |  |  | |  |
| --- | --- | --- | --- | --- | --- |
|  |  | B (SE) | β UK | β Japan | |
| Extraversion | E1 | 1.00 | .87 | .81 | |
|  | E2 | 1.02^***^ (.02) | .90 | .85 | |
|  | E3 | .97^***^ (.03) | .86 | .84 | |
|  | E4 | .90^***^ (.02) | .84 | .79 | |
|  | E5 | 1.00^***^ (.03) | .86 | .86 | |
|  | E6 | .48^***^ (.03) | .45 | .40 | |
|  | E7 | .95^***^ (.03) | .83 | .85 | |
|  | E8 | .89^***^ (.02) | .84 | .77 | |
| Intimacy | I1 | 1.00 | .73 | .70 | |
|  | I2 | .87^***^ (.04) | .67 | .63 | |
|  | I3 | .82^***^ (.04) | .62 | .56 | |
|  | I4 | .97^***^ (.05) | .80 | .71 | |
|  | I5 | .92^***^ (.05) | .81 | .71 | |
|  | I6 | .70^***^ (.04) | .48 | .50 | |
| Positive Group Identification | GP1 | 1.00 | .59 | .52 | |
|  | GP2 | 1.65^***^ (.12) | .77 | .78 | |
|  | GP3 | 1.88^***^ (.13) | .85 | .84 | |
|  | GP4 | 1.28^***^ (.09) | .68 | .69 | |
|  | GP5 | 1.57^***^ (.12) | .70 | .72 | |
| Negative Group Identification | GN1 | 1.00 | .68 | .73 | |
|  | GN2 | .77^***^ (.07) | .54 | .50 | |
|  | GN3 | .91^***^ (.06) | .64 | .72 | |
|  | GN4 | .86^***^ (.06) | .63 | .63 | |
| Friendship Styles | Extra | 1.00 | .63 | .79 | |
|  | Intimacy | .65^***^ (.05) | .59 | .63 | |
|  | Positive Group I | .62^***^  (.06) | .84 | .91 | |
|  | Negative Group I | 1.10^***^ (.04) | 1.00 | 1.00 | |

We decided to adjust one of the factor loadings between groups based on the highest modification index for Japan, as it had a slightly worse fit based on chi-square. Modification indices suggested that GP4 should be allowed to vary between groups. GP4 states how well an individual sees groups of friends working together whereas the other items assessing positive group identification examine how one feels and identifies with a specific friendship group. Therefore, an item which asks about how well groups work together may be interpreted differently across cultures. Allowing GP4 to vary in the model kept the model fit at an acceptable level while meeting Chen’s (2007) difference criteria. This model with GP4 variance was accepted (see Table S1i for factor loadings).

**Table S1i**

*Factor Loadings for the Four-Factor Model of Friendship Styles (Extraversion, Enjoyment of Intimacy, Positive Group Identification, Negative Group Identification) with a Common Bias Factor at Partial-Metric Level of Measurement Invariance for the UK and Japan*

| Factor | Item |  |  |  | |
| --- | --- | --- | --- | --- | --- |
|  |  | B (SE) | β UK | | β Japan |
| Extraversion | E1 | 1.00 | .87 | | .81 |
|  | E2 | 1.02^***^ (.02) | .90 | | .85 |
|  | E3 | .97^***^ (.03) | .86 | | .84 |
|  | E4 | .90^***^ (.02) | .84 | | .78 |
|  | E5 | 1.00^***^ (.03) | .86 | | .86 |
|  | E6 | .48^***^ (.03) | .45 | | .40 |
|  | E7 | .95^***^ (.03) | .83 | | .85 |
|  | E8 | .89^***^ (.02) | .84 | | .77 |
| Intimacy | I1 | 1.00 | .73 | | .70 |
|  | I2 | .87^***^ (.04) | .67 | | .63 |
|  | I3 | .82^***^ (.04) | .62 | | .56 |
|  | I4 | .97^***^ (.05) | .80 | | .71 |
|  | I5 | .92^***^ (.05) | .81 | | .71 |
|  | I6 | .70^***^ (.04) | .48 | | .49 |
| Positive Group Identification | GP1 | 1.00 | .59 | | .49 |
|  | GP2 | 1.70^***^ (.13) | .79 | | .77 |
|  | GP3 | 1.94^***^ (.14) | .87 | | .83 |
|  | GP4 | UK: 1.01^***^ (.09)  Japan: 1.63^***^ (.14) | .59 | | .77 |
|  | GP5 | 1.60^***^ (.13) | .71 | | .71 |
| Negative Group Identification | GN1 | 1.00 | .68 | | .73 |
|  | GN2 | .77^***^ (.07) | .53 | | .50 |
|  | GN3 | .92^***^ (.06) | .65 | | .72 |
|  | GN4 | .87^***^ (.06) | .64 | | .63 |
| Friendship Styles | Extra | 1.00 | .63 | | .78 |
|  | Intimacy | .66^***^ (.05) | .59 | | .62 |
|  | Positive Group I | .60^***^  (.06) | .82 | | .92 |
|  | Negative Group I | 1.09^***^ (.04) | 1.00 | | 1.00 |

After accepting the partial-metric invariance model, we assessed scalar invariance. However, there were quite large violations of Chen’s (2007) criteria (see Table S1g). The added restrictions of making the intercepts equal across countries produced a warning in about the model’s covariance-variance matrix not being positive definite because of eigenvalues close to zero, despite a model being identified with all R^2^ values produced. We therefore rejected this model (see Table S1j for intercepts) and examined if any intercepts could be released to achieve an acceptable partial-scalar model based on Chen’s (2007) goodness-of-fit criteria.

**Table S1j**

*Intercepts for the Four-Factor Model of Friendship Styles (Extraversion, Enjoyment of Intimacy, Positive Group Identification, Negative Group Identification) with a Common Bias Factor at Scalar Level of Measurement Invariance for the UK and Japan*

| Factor | Item | Intercept | Standardized Intercept | |
| --- | --- | --- | --- | --- |
|  |  |  | UK | Japan |
| Extraversion | E1 | 4.21^***^ (.08) | 2.34 | 2.65 |
|  | E2 | 4.10^***^ (.08) | 2.29 | 2.63 |
|  | E3 | 3.45^***^ (.08) | 1.94 | 2.31 |
|  | E4 | 4.40^***^ (.07) | 2.63 | 2.98 |
|  | E5 | 3.56^***^ (.08) | 1.95 | 2.37 |
|  | E6 | 4.00^***^ (.06) | 2.41 | 2.60 |
|  | E7 | 3.54^***^ (.07) | 1.97 | 2.47 |
|  | E8 | 4.28^***^ (.07) | 2.52 | 2.81 |
| Intimacy | I1 | 5.18^***^ (.06) | 3.58 | 3.48 |
|  | I2 | 5.20^***^ (.06) | 3.59 | 3.46 |
|  | I3 | 5.45^***^ (.06) | 3.76 | 3.49 |
|  | I4 | 5.47^***^ (.06) | 3.96 | 3.64 |
|  | I5 | 5.84^***^ (.05) | 4.94 | 4.35 |
|  | I6 | 4.65^***^ (.06) | 2.97 | 3.16 |
| Positive Group Identification | GP1 | 5.64^***^ (.06) | 4.17 | 3.97 |
|  | GP2 | 5.12^***^ (.07) | 3.32 | 3.56 |
|  | GP3 | 5.03^***^ (.07) | 3.11 | 3.28 |
|  | GP4 | 5.35^***^ (.05) | 4.28 | 3.91 |
|  | GP5 | 4.70^***^ (.07) | 2.90 | 3.18 |
| Negative Group Identification | GN1 | 4.89^***^ (.07) | 3.06 | 3.21 |
|  | GN2 | 5.28^***^ (.09) | 3.07 | 2.78 |
|  | GN3 | 5.08^***^ (.05) | 3.41 | 3.80 |
|  | GN4 | 5.17^***^ (.05) | 3.64 | 3.57 |
| Friendship Styles | Extra | .37^***^ (.06) |  | .29 |
|  | Intimacy | -.43^***^ (.07) |  | -.43 |
|  | Positive Group I | -.04 (.04) |  | -.05 |
|  | Negative Group I | -.56^***^ (.07) |  | -.49 |
| Friendship Styles | Overall | -.69^***^ (.06) |  | -.68 |
| Common Bias Factor | Style | -.37^***^ (.03) |  | -1.13 |

Positive Group *p* = .253

Modification indices suggested that we needed to sequentially release 5 item intercepts, E7, I5, GP1, GN1 and GN2, (see Table S1k) in order to meet Chen’s (2007) criteria. However, we still encountered the lavaan warning that our model’s covariance-variance matrix might not be positive definite. We retained the partial scalar models despite this warning (common at this level of measurement invariance) for the following reasons: (1) FHQ scores correlated with other variables measuring friendship styles, (2) the partial-scalar model made theoretical sense, was an identifiable model with acceptable model fits, (3) analyses examining FHQ scores in the UK and Japan using the partial-scalar SEM yielded results similar to other measures of friendship styles and to analyses using t-tests, and (4) the significant difference in response styles on the partial-scalar model might indicate this is contributing to variation in the data.

It appears that for a given level of extraversion and intimacy, Japanese participants scored slightly higher on E7 and I5. Whereas, for a given level of identification, British participants scored higher on GP1, GN1 and GN2 (see Table S1k). Once sequentially released, this partial-scalar model achieved an acceptable level of invariance. The intercepts suggested that Japan scored higher than the UK on extraversion (.14, *p* = .022) but lower on intimacy (-.73, *p* < .001) and positive group identification (-.14, *p* < .001). There was no difference in negative group identification between the UK and the Japan (.08, *p* = .184). Overall, FHQ scores were lower in Japan than the UK (-.57, *p* < .001) suggesting that participants from the UK have more group-oriented friendship styles than participants from Japan. In addition, analyses of the response style suggested that respondents from the UK were more likely to agree with the FHQ statements than the Japanese respondents (-.26, *p* < .001). A diagram of the overall model is provided in Figure S1.

**Table S1k**

*Intercepts for* *the Four-Factor Model of Friendship Styles (Extraversion, Enjoyment of Intimacy, Positive Group Identification, Negative Group Identification) with a Common Bias Factor at Partial-Scalar Level of Measurement Invariance for the UK and Japan*

| Factor | Item | Intercept | Standardized Intercept | |
| --- | --- | --- | --- | --- |
|  |  |  | UK | Japan |
| Extraversion | E1 | 4.21^***^ (.08) | 2.35 | 2.66 |
|  | E2 | 4.11^***^ (.08) | 2.30 | 2.64 |
|  | E3 | 3.58^***^ (.07) | 2.02 | 2.41 |
|  | E4 | 4.39^***^ (.07) | 2.64 | 3.00 |
|  | E5 | 3.70^***^ (.07) | 2.03 | 2.46 |
|  | E6 | 3.96^***^ (.06) | 2.39 | 2.58 |
|  | E7 | UK: 3.28^***^ (.07)  Japan: 3.88^***^ (.08) | 1.82 | 2.69 |
|  | E8 | 4.27^***^ (.07) | 2.52 | 2.81 |
| Intimacy | I1 | 5.22^***^ (.06) | 3.66 | 3.56 |
|  | I2 | 5.25^***^ (.06) | 3.67 | 3.54 |
|  | I3 | 5.49^***^ (.05) | 3.82 | 3.55 |
|  | I4 | 5.53^***^ (.06) | 4.07 | 3.75 |
|  | I5 | UK: 5.73^***^ (.05)  Japan: 6.25^***^ (.07) | 4.68 | 4.58 |
|  | I6 | 4.67^***^ (.06) | 3.00 | 3.19 |
| Positive Group Identification | GP1 | UK: 5.94^***^ (.05)  Japan: 5.12^***^ (.07) | 4.79 | 3.88 |
|  | GP2 | 5.09^***^ (.07) | 3.25 | 3.56 |
|  | GP3 | 5.00^***^ (.07) | 3.05 | 3.28 |
|  | GP4 | 5.32^***^ (.05) | 4.26 | 3.88 |
|  | GP5 | 4.67^***^ (.06) | 2.85 | 3.17 |
| Negative Group Identification | GN1 | UK: 4.94^***^ (.07)  Japan: 4.18^***^ (.09) | 3.10 | 2.76 |
|  | GN2 | UK: 5.63^***^ (.07)  Japan: 4.07^***^ (.09) | 3.61 | 2.41 |
|  | GN3 | 4.84^***^ (.06) | 3.15 | 3.44 |
|  | GN4 | 5.00^***^ (.06) | 3.41 | 3.32 |
| Friendship Styles | Extra | .14^*^ (.06) |  | .11 |
|  | Intimacy | -.73^***^ (.06) |  | -.76 |
|  | Positive Group I | -.14^***^ (.04) |  | -.21 |
|  | Negative Group I | .08 (.06) |  | .07 |
| Friendship Styles | Overall | -.56^***^ (.05) |  | -.56 |
| Common Factor Bias | Style | -.26^***^ (.03) |  | -.79 |

Extra p = .022, Negative Group p = .124

**Figure S1**

*Path Analysis for the Four-Factor Model of Friendship Styles (Extraversion, Enjoyment of Intimacy, Positive Group Identification, Negative Group Identification)*


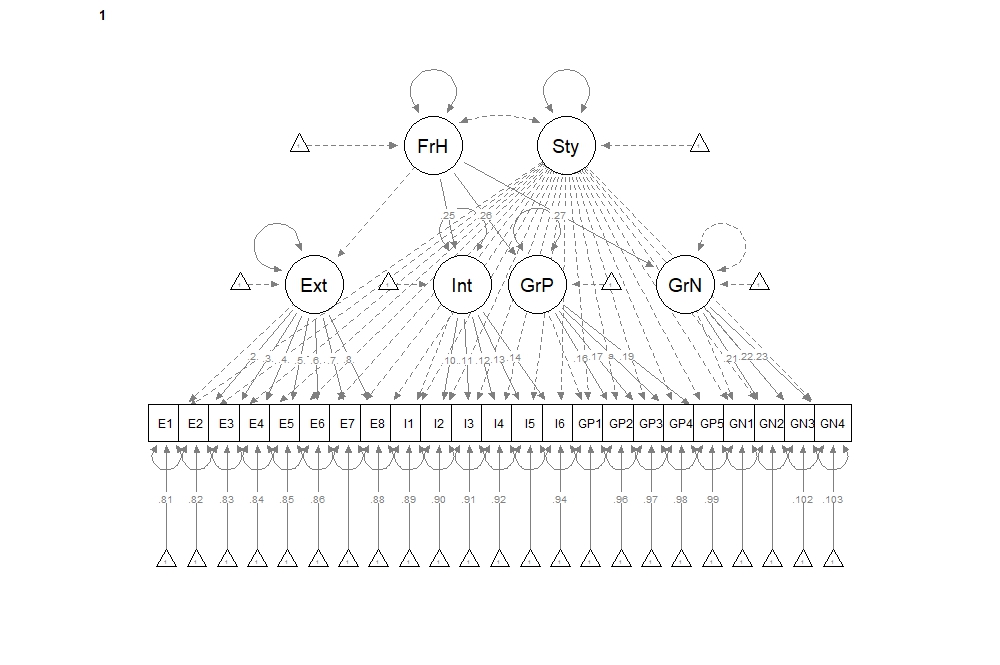


**S3. Study 1: Self-reported Friendship Group Sizes and FHQ Scores**

To further explore the profiles of respondents socializing in larger friendship groups, we transformed participants’ numeric responses of friendship group size into a 4-level categorical variable (1 friend or dyadic interaction, 2 friends, 3 friends, 4 or more friends) as an independent variable and investigated how FHQ scores differed by group size. The ANOVA revealed a significant interaction between country and number of friends in a typical social interaction, *F*(3, 1001) = 2.86, *p* = .036, η_p_² = .009. This interaction is presented in the graph below. To explore the differences in number of friends in a social interaction, we conducted the analysis separately for each country (see Figure S2 for results).

**Figure S2**

*Typical Number of Friends in Interaction as a Function of FHQ Scores*


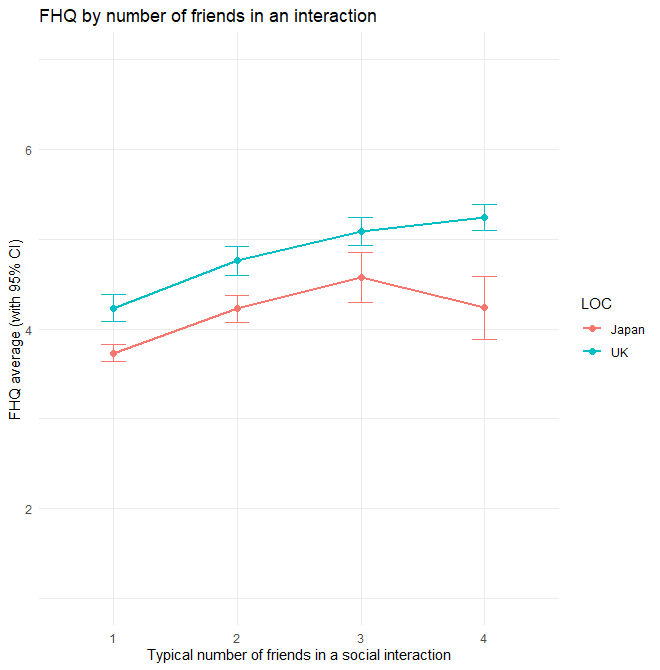


The ANOVA in the UK found that number of friends in social interactions was a significant predictor of FHQ score, *F*(3, 500) = 36.64, *p* < .001, η_p_² = .180. Post-hoc analyses with Bonferroni correction found that people who socialized in dyads were significantly lower on FHQ scores than all other group sizes (all *p* < .001). People who socialized with two friends at a time also differed from those who socialized with 3 friends (*p* = .029) and 4 friends at a time (*p* < .001). People who socialized with three friends were not significantly different from those who socialized with four friends (*p* = 1.00). Overall, this suggests that FHQ differ between people who socialize in dyads (one friend at a time) and groups in the UK. Increases with friendship group sized may also be associated with an increase in FHQ scores, but it is possible that the scores plateau as the group size gets bigger.

The ANOVA in Japan found that number of friends in social interactions was a significant predictor of FHQ score, *F*(3, 501) = 20.15, *p* < .001, η_p_² = .108. Post-hoc analyses with Bonferroni correction found that people who socialized in dyads had significantly lower FHQ scores than people who socialized with two or three (both *p* < .001) or four friends (*p* = .004). People who socialized with two friends at a time did not significantly differ in FHQ scores from 3 friends (*p* = .103) and 4 friends at a time (*p* = 1.00). People who socialized with three friends were also not significantly different from people who socialized with four friends (*p* = .374). Overall, this suggests that in Japan, FHQ scores differ between people who socialize in dyads (one friend at a time) and groups, but the scores may not necessarily covary with the size of larger friendship groups.

**S4. Study 1: Measurement Invariance for the Relational Mobility Scale**

The 12 items of the Relational Mobility Scale (RMS; Thomson et al., 2018) are presented in Table S2a. Items are designated with a code (e.g., RMS1, RMS2 etc.) that we will use for the rest of the supplementary materials. All 12 items were retained throughout the measurement invariance process.

**Table S2a**

*Items and Dimensions of the Relational Mobility Scale (Thomson et al., 2018).*

| Meeting |
| --- |
| RMS1 They (the people around you) have many chances to get to know other people. |
| RMS2 It is common for these people to have a conversation with someone they have never met before |
| RMS4 There are few opportunities for these people to form new friendships* |
| RMS5 It is uncommon for these people to have a conversation with people they have never met before* |
| RMS8 It is easy for them to meet new people |
| Choosing |
| RMS3 They are able to choose, according to their own preferences, the people whom they interact with in their daily life |
| RMS6 If they did not like their current groups, they could leave for better ones |
| RMS7 It is often the case that they cannot freely choose who they associate with* |
| RMS9 Even if these people were not completely satisfied with the group they belonged to, they would usually stay with it anyway* |
| RMS10 They are able to choose the groups and organizations they belong to |
| RMS11 Even if these people were not satisfied with their current relationships, they would often have no choice but to stay with them* |
| RMS12 Even though they might rather leave, these people often have no choice but to stay in groups they don’t like* |

** Reverse-scored items*

We used the two-level model of the relational mobility scale proposed by Thomson et al. (2018) with two first-order factors (choosing and meeting) loading onto a second-order factor of relational mobility. In addition, the model includes a common bias factor of response style, with reverse scored items factor loadings set to -1 and other items to 1. The factor was specified to not correlate with the latent factors of choosing and meeting. This model included two initial residual correlations (RMS2 with RMS5; RMS11 with RMS12) applied to all groups (UK and Japan), resulting in four residual correlations. The data had high kurtosis and skewness on a Mardia’s test, so we used a robust maximum likelihood estimator [MLR] and provide robust indices for χ², CFI, TLI and RMSEA. When looking at the chi-square separately for the two countries, it appears that model fit for Japan, χ² =181.79, was slightly less acceptable than the model fit for the UK, χ² = 93.44. Goodness-of-fit indices showed an excellent fit for both Japan and the UK (see Table S2b). We accepted this initial configural model (see Table S2c for factor loadings) and began the measurement invariance testing process (see Table S2d).

**Table S2b**

*Model Fit Indices for the Two-Factor Model of Relational Mobility (Meeting and Choosing) with a Common Bias Factor for both Japan and the UK, and the Sample Overall*

| Model | χ² | df | CFI | TLI | SRMR | RMSEA | RMSEA 90% CI |  |
| --- | --- | --- | --- | --- | --- | --- | --- | --- |
| Japan | 173.07^***^ | 50 | .936 | .916 | .051 | .080 | (.067, .093) |  |
| UK | 98.40^***^ | 50 | .970 | .961 | .039 | .048 | (.034, .062) |  |
| Overall | 275.23^***^ | 100 | .951 | .935 | .042 | .066 | (.057, .075) |  |
| *^***^p < .001.* | | | | | | | | |

**Table S2c**

*Study 1: Factor Loadings for the Two-Factor Model of Relational Mobility (Meeting and Choosing) with a Common Bias Factor at the Configural Level of Measurement Invariance*

| Factor | Item | UK | | Japan | |
| --- | --- | --- | --- | --- | --- |
|  |  | B (SE) | β | B (SE) | β |
| Meeting | RMS1 | 1.00 | .74 | 1.00 | .77 |
|  | RMS2 | .69^***^ (.08) | .50 | .69^***^ (.06) | .55 |
|  | RMS4 | 1.01^***^ (.07) | .69 | .95^***^ (.08) | .72 |
|  | RMS5 | .69^***^ (.08) | .48 | .73^***^ (.06) | .56 |
|  | RMS8 | 1.02^***^ (.05) | .79 | .99^***^ (.07) | .75 |
| Choosing | RMS3 | 1.00 | .62 | 1.00 | .64 |
|  | RMS6 | .86^***^ (.11) | .51 | .97^***^ (.09) | .62 |
|  | RMS7 | 1.19^***^ (.10) | .64 | 1.13^***^ (.09) | .68 |
|  | RMS9 | .90^***^ (.11) | .53 | 1.08^***^ (.12) | .73 |
|  | RMS10 | 1.01^***^ (.09) | .65 | 1.10^***^ (.07) | .68 |
|  | RMS11 | 1.16^***^ (.13) | .63 | 1.15^***^ (.13) | .73 |
|  | RMS12 | 1.23^***^ (.12) | .66 | 1.12^***^ (.13) | .71 |
| Covariances |  |  |  |  |  |
| RMS2 ~~ RMS5 |  | .51^***^ (.06) | .51 | .22^***^ (.04) | .35 |
| RMS11 ~~ RMS12 |  | .22^***^ (.06) | .30 | .15^***^ (.04) | .31 |

**Table S2d**

*The Measurement Invariance Testing Process for the Two-Factor Model of Relational Mobility with a Common Bias Factor. Table Shows Robust Model Fit Indices at Different Levels of Measurement Invariance*

| Model | | χ²(df) | Δχ²(df) | CFI | TLI | SRMR | RMSEA | | RMSEA 90% CI | Decision |  |
| --- | --- | --- | --- | --- | --- | --- | --- | --- | --- | --- | --- |
| Configural | | 275.23(100)^***^ |  | .951 | .935 | .042 | .066 | (.057, .075) | | Accept |  |
| Metric | | 284.47(110)^***^ | 7.93(10) ^***^ | .951 | .942 | .045 | .063 | (.054, .072) | | Accept |  |
| Scalar | | 398.76(118)^***^ | 127.01(8) ^***^ | .923 | .913 | .056 | .076 | (.068, .085) | | Reject |  |
| Partial Scalar (RMS9 intercept varied) | | 324.78(117)^***^ | 43.36(7) ^***^ | .943 | .935 | .048 | .066 | (.058, .075) | | Accept |  |
|  | *^***^p < .001.* | | | | | | | | | | |

As noted in Table S2d, the model was accepted at metric level of variance testing with no additional modifications needed (see Table S2e for factor loadings).

**Table S2e**

*Study 1: Factor Loadings for the Two-Factor Model of Relational Mobility with a Common Bias Factor at the Metric Level of Measurement Invariance*

| Factor | Item |  |  | |  |
| --- | --- | --- | --- | --- | --- |
|  |  | B (SE) | β UK | β Japan | |
| Meeting | RMS1 | 1.00 | .75 | .76 | |
|  | RMS2 | .69^***^ (.05) | .51 | .55 | |
|  | RMS4 | .98^***^ (.05) | .68 | .72 | |
|  | RMS5 | .72^***^ (.05) | .50 | .55 | |
|  | RMS8 | 1.01^***^ (.04) | .79 | .75 | |
| Choosing | RMS3 | 1.00 | .61 | .64 | |
|  | RMS6 | .93^***^ (.07) | .53 | .61 | |
|  | RMS7 | 1.16^***^ (.07) | .62 | .69 | |
|  | RMS9 | 1.02^***^ (.08) | .57 | .71 | |
|  | RMS10 | 1.07^***^ (.06) | .66 | .67 | |
|  | RMS11 | 1.15^***^ (.09) | .62 | .73 | |
|  | RMS12 | 1.16^***^ (.09) | .63 | .73 | |
| Covariances |  |  |  |  | |
| RMS2 ~~ RMS5 |  | UK: .50^***^ (.06) Japan: .22^***^ (.04) | .51 | .35 | |
| RMS11 ~~ RMS12 |  | UK: .24^***^ (.06) Japan: .14^***^ (.04) | .31 | .30 | |

Although we achieved both configural and metric measurement invariance with relative ease, the model failed Chen’s criteria of goodness-of-fit indices (see Table S2d) and did not achieve scalar invariance (see Table S2f for intercepts). In addition, similar to testing the measurement invariance of the FHQ, the added restrictions of making the intercepts equal across countries produced a warning in lavaan that the model’s covariance-variance matrix might not be positive definite because of eigen values close to zero, despite a model being identified (as evidenced by all R^2^ values being computed). We therefore investigated if releasing intercepts would improve model fit.

**Table S2f**

*Study 1: Intercepts for the Two-Factor Model of Relational Mobility with a Common Bias Factor at the Scalar Level of Measurement Invariance*

| Factor | Item | Intercept | Standardized Intercepts | |
| --- | --- | --- | --- | --- |
|  |  |  | UK | Japan |
| Meeting | RMS1 | 4.17^***^ (.05) | 3.67 | 4.10 |
|  | RMS2 | 4.12^***^ (.04) | 3.58 | 4.25 |
|  | RMS4 | 3.92^***^ (.05) | 3.23 | 3.80 |
|  | RMS5 | 4.09^***^ (.04) | 3.37 | 4.08 |
|  | RMS8 | 4.10^***^ (.05) | 3.83 | 4.02 |
| Choosing | RMS3 | 4.30^***^ (.04) | 4.23 | 4.18 |
|  | RMS6 | 4.10^***^ (.04) | 3.79 | 4.09 |
|  | RMS7 | 3.88^***^ (.05) | 3.32 | 3.48 |
|  | RMS9 | 3.47^***^ (.05) | 3.06 | 3.70 |
|  | RMS10 | 4.34^***^ (.04) | 4.29 | 4.12 |
|  | RMS11 | 3.82^***^ (.05) | 3.19 | 3.56 |
|  | RMS12 | 3.74^***^ (.05) | 3.18 | 3.46 |
| Relational Mobility | Meeting | -.04 (.04) |  | -.05 |
|  | Choosing | -.16^***^ (.03) |  | -.24 |
| Relational Mobility | Overall | -.20^***^ (.03) |  | -.41 |
| Common Factor Bias | Style | .01 (.02) |  | .05 |
| Covariances |  |  |  |  |
| RMS2 ~~ RMS5 |  | UK: .50^***^ (.06) Japan: .22^***^ (.04) | .51 | .35 |
| RMS11 ~~ RMS12 |  | UK: .27^***^ (.06) Japan: .16^***^ (.04) | .31 | .32 |

Meeting *p* = .236, Style *p* = .605.

Modification indices suggested that we should release one intercept. After sequentially releasing just one intercept (RMS9), we achieved partial-scalar invariance (see Table S2g for intercepts). However, the warning from laavan about a non-positive definite covariance-variance matrix remained. Nonetheless, as with FHQ, we accepted this partial-scalar model. It appears that for a given level of choosing, Japanese participants scored slightly higher on RMS9. The intercepts suggested Japan scored significantly lower on having freedom to choose friends (-.19 *p* < .001) than the UK. There was no difference in perceived opportunity to meet people between Japan and the UK (-.04, *p* = .320). Overall, relational mobility was smaller in Japan than the UK (-.23, *p* < .001) suggesting that respondents in the UK perceive more opportunities and freedom to meet and choose their friends. In addition, the analysis of the common bias factor of response style suggested that Japanese participants were more likely to demonstrate a response style bias than participants from the UK (.05, *p* = .033).

**Table S2g**

*Study 1: Intercepts for the Two-Factor of Relational Mobility with a Common Bias Factor at the Partial-scalar Level of Measurement Invariance*

| Factor | Item | Intercept | Standardized Intercepts | |
| --- | --- | --- | --- | --- |
|  |  |  | UK | Japan |
| Meeting | RMS1 | 4.16^***^ (.05) | 3.66 | 4.09 |
|  | RMS2 | 4.11^***^ (.04) | 3.57 | 4.24 |
|  | RMS4 | 3.95^***^ (.05) | 3.25 | 3.83 |
|  | RMS5 | 4.12^***^ (.04) | 3.40 | 4.11 |
|  | RMS8 | 4.09^***^ (.05) | 3.82 | 4.02 |
| Choosing | RMS3 | 4.31^***^ (.04) | 4.26 | 4.21 |
|  | RMS6 | 4.11^***^ (.04) | 3.80 | 4.12 |
|  | RMS7 | 3.93^***^ (.05) | 3.37 | 3.55 |
|  | RMS9 | UK: 3.19^***^ (.05) Japan: 3.75^***^ (.06) | 1.84 | 3.91 |
|  | RMS10 | 4.36^***^ (.04) | 4.32 | 4.15 |
|  | RMS11 | 3.90^***^ (.05) | 3.29 | 3.67 |
|  | RMS12 | 3.81^***^ (.05) | 3.27 | 3.56 |
| Relational Mobility | Meeting | -.04 (.04) |  | -.05 |
|  | Choosing | -.19^***^ (.03) |  | -.30 |
| Relational Mobility | Overall | -.23^***^ (.03) |  | -.46 |
| Common Factor Bias | Style | .05^*^ (.02) |  | .20 |
| Covariances |  |  |  |  |
| RMS2 ~~ RMS5 |  | UK: .50^***^ (.06) Japan: .22^***^ (.04) | .51 | .35 |
| RMS11 ~~ RMS12 |  | UK: .23^***^ (.06) Japan: .13^***^ (.04) | .30 | .29 |

Meeting *p* = .320, Style *p* = .033.

**S5. Study 1: Measurement Invariance for the Analysis-Holism Scale**

We tested the measurement invariance of the Analysis-Holism Scale (Choi et al., 2007), using the same method as for the Friendship Habits Questionnaire and Relational Mobility Scale. Specifically, we examined the fit of a two-level model with four first-order factors (causality, attitude, change and attention) loading onto a second-order factor of holistic, versus analytic, thinking. In addition, for consistency, we added the common bias factor to account for differences in response style. Our data had high kurtosis and skewness on a Mardia’s test, so we used a robust maximum likelihood estimator [MLR] and provide robust indices for χ², CFI, TLI and RMSEA. The model fit was poor (see Table S3). An inspection of the chi-square separately for the two countries revealed that the model had a less acceptable fit in Japan, χ² = 870.02, than in the UK, χ² = 658.22. In other words, the scale did not achieve the configural level of measurement invariance. Given this finding, as well as the fact that the Analysis-Holism Scale had lower reliability (overall: α = .71, *ω* = .74; Japan: α = .74, *ω* = .77; UK: α = .68, *ω* = .70) than the Friendship Habits Questionnaire (overall: α = .94 , *ω* = .94; Japan: α = .93, *ω* = .93; UK: α = .92, *ω* = .92) and the Relational Mobility Scale (overall: α = .86, *ω* = .86; Japan: α = .85, *ω* = .85; UK: α = .85, *ω* = .85), we did not test for more stringent levels of measurement invariance.

**Table S3**

*Model Fit Indices for the Four-Factor Analysis-Holism Model (Causality, Attitude, Change and Direction) for Both Japan and the UK, and the Entire Sample*

| Model | χ² | df | CFI | TLI | SRMR | RMSEA | RMSEA 90% CI |  |
| --- | --- | --- | --- | --- | --- | --- | --- | --- |
| Japan | 943.23^***^ | 247 | .720 | .687 | .096 | .080 | (.074, .085) |  |
| UK | 667.35^***^ | 247 | .810 | .788 | .076 | .062 | (.057, .068) |  |
| Overall | 1628.13^***^ | 494 | .762 | .734 | .083 | .072 | (.068, .076) |  |
| *^***^p < .001.* | | | | | | | | |

**S6: Study 1: Alternative Hypothesis Testing with the Combined Friendship Habits and Relational Mobility Models**

To test the study hypotheses, we further combined the measurement invariance models of the Friendship Habits Questionnaire and the Relational Mobility Scale. Specifically, the analysis included the second-order partial scalar model of the FHQ with four first-order dimensions (extraversion, intimacy, positive group identification and negative identification) which loaded onto a second-order factor, known as friendship styles, with the modified edits through measurement invariance (GP4 factor loading varied; E7, I5, GP1, GN1 & GN2 intercept varied). We also included the second-order partial scalar model of the RMS with two first-order dimensions (meeting and choosing) with four covariances, which loaded onto a second-order factor of relational mobility, with the modified edits through measurement invariance (RMS9 intercept varied). We kept the common biases factors separate for both FHQ and RMS. In addition to the measurement invariance models, we included the size of friendship groups, proportion of time in friendship groups, and participants’ age as indicator-level variables. This new model served to analyze correlations between measures and country differences in friendship styles as well as relational mobility.

The data had high kurtosis and skewness, as evidenced by a Mardia’s test. We therefore used a robust maximum likelihood estimator [MLR] and provide robust indices for χ², CFI, TLI and RMSEA. The fit of this new structural equation model was good for RMSEA and SRMR and borderline acceptable for CFI and TLI (see Table S4a). When looking at the chi-square separately for the two countries, it appears that the model fit was less acceptable in Japan, χ² = 1723.35, than in the UK, χ² = 1407.52. Model intercepts can be found in Table S4b. This model did not produce any lavaan warnings.

**Table S4a**

*Study 1: Model Fit Indices for the Hypothesis Testing Structural Equation Model for the Overall Sample*

| Model | χ² | df | CFI | TLI | SRMR | RMSEA | RMSEA 90% CI |  |
| --- | --- | --- | --- | --- | --- | --- | --- | --- |
| SEM | 3130.51^***^ | 1350 | .899 | .895 | .065 | .054 | (.052, .056) |  |
| *^***^p < .001.* | | | | | | | | |

**Table S4b**

*Study 1: Intercepts for the Hypothesis Testing Structural Equation Model*

| Factor | Item |  | Standardized Intercept | |
| --- | --- | --- | --- | --- |
|  |  | Intercept | UK | Japan |
| Extraversion | E1 | 4.24^***^ (.08) | 2.36 | 2.67 |
|  | E2 | 4.12^***^ (.08) | 2.31 | 2.64 |
|  | E3 | 3.60^***^ (.07) | 2.03 | 2.42 |
|  | E4 | 4.41^***^ (.07) | 2.65 | 3.00 |
|  | E5 | 3.72^***^ (.08) | 2.05 | 2.47 |
|  | E6 | 3.98^***^ (.06) | 2.39 | 2.58 |
|  | E7 | UK: 3.29^***^ (.07)  Japan: 3.91^***^ (.08) | 1.82 | 2.70 |
|  | E8 | 4.29^***^ (.07) | 2.53 | 2.81 |
| Intimacy | I1 | 5.22^***^ (.06) | 3.67 | 3.55 |
|  | I2 | 5.28^***^ (.06) | 3.68 | 3.57 |
|  | I3 | 5.50^***^ (.06) | 3.80 | 3.54 |
|  | I4 | 5.55^***^ (.06) | 4.08 | 3.76 |
|  | I5 | UK: 5.75^***^ (.05)  Japan: 6.25^***^ (.07) | 4.73 | 4.60 |
|  | I6 | 4.68^***^ (.06) | 2.97 | 3.19 |
| Positive Group Identification | GP1 | UK: 5.93^***^ (.05)  Japan: 5.14^***^ (.07) | 4.77 | 3.87 |
|  | GP2 | 5.11^***^ (.07) | 3.28 | 3.55 |
|  | GP3 | 5.02^***^ (.07) | 3.07 | 3.28 |
|  | GP4 | 5.33^***^ (.05) | 4.30 | 3.87 |
|  | GP5 | 4.68^***^ (.06) | 2.87 | 3.16 |
| Negative Group Identification | GN1 | UK: 4.97^***^ (.07)  Japan: 4.19^***^ (.09) | 3.13 | 2.78 |
|  | GN2 | UK: 5.62^***^ (.07)  Japan: 4.08^***^ (.09) | 3.60 | 2.41 |
|  | GN3 | 4.85^***^ (.07) | 3.17 | 3.47 |
|  | GN4 | 5.01^***^ (.06) | 3.41 | 3.33 |
| Friendship Styles | Extra | .17^*^ (.06) |  | .13 |
|  | Intimacy | -.72^***^ (.06) |  | -.75 |
|  | Positive Group I | -.12^**^ (.04) |  | -.17 |
|  | Negative Group I | .11 (.06) |  | .10 |
| Friendship Styles | FHQ | -.62^***^ (.05) |  | -.62 |
| Common Bias Factor FHQ | Style1 | -.25^***^ (.03) |  | -.78 |
| Meeting | RMS1 | 4.16^***^ (.05) | 3.69 | 4.12 |
|  | RMS2 | 4.11^***^ (.04) | 3.57 | 4.23 |
|  | RMS4 | 3.95^***^ (.05) | 3.26 | 3.83 |
|  | RMS5 | 4.11^***^ (.04) | 3.41 | 4.10 |
|  | RMS8 | 4.09^***^ (.05) | 3.84 | 4.04 |
| Choosing | RMS3 | 4.31^***^ (.04) | 4.24 | 4.17 |
|  | RMS6 | 4.11^***^ (.04) | 3.80 | 4.11 |
|  | RMS7 | 3.93^***^ (.05) | 3.37 | 3.54 |
|  | RMS9 | UK: 3.19^***^ (.05) Japan: 3.75^***^ (.06) | 2.84 | 3.90 |
|  | RMS10 | 4.36^***^ (.04) | 4.32 | 4.13 |
|  | RMS11 | 3.90^***^ (.05) | 3.28 | 3.65 |
|  | RMS12 | 3.81^***^ (.05) | 3.25 | 3.54 |
| Relational Mobility | Meeting | -.03 (.04) |  | -.04 |
|  | Choosing | -.20^***^ (.03) |  | -.30 |
| Relational Mobility | RMS | -.23^***^ (.03) |  | -.47 |
| Common Bias Factor RMS | Style2 | .05^*^ (.02) |  | .20 |
| Friendship Group Size | Size | 2.67^***^ (.08) | 1.53 | 2.21 |
| Friendship Group Size | Overall | -.98^***^ (.09) |  | -.82 |
| Proportion Time in Groups | Prop | 49.94^***^ (1.15) | 1.90 | 1.90 |
| Proportion Time in Groups | Overall | -17.01^***^ (1.63) |  | -.65 |
| Age | Age | 22.02^***^ (.18) | 5.49 | 5.05 |
| Age | Overall | 7.72^***^ (.26) |  | 1.77 |
| Covariances |  |  |  |  |
| RMS2 ~~ RMS5 |  | UK: .50^***^ (.06) Japan: .22^***^ (.04) | .51 | .36 |
| RMS11 ~~ RMS12 |  | UK: .24^***^ (.06) Japan: .14^***^ (.04) | .31 | .29 |
| Friendship Styles ~~ Proportion |  | UK: 12.93^***^ (1.52) Japan: 9.14^***^ (1.42) | .50 | .35 |
| ~~ Size |  | UK: .69^***^ (09)  Japan: .30^***^ (.07) | .40 | .25 |
| ~~ RMS |  | UK: .23^***^ (.04) Japan: .17^***^ (.04) | .40 | .34 |
| ~~ Age |  | UK: -.45^*^ (.21)  Japan: -.05 (.20) | -.11 | -.01 |
| Proportion ~~ Size |  | UK: 21.71^***^ (2.14) Japan: 13.28^***^ (1.62) | .48 | .42 |
| ~~ RMS |  | UK: 2.10^**^ (.80) Japan: 1.96^*^ (.78) | .14 | .15 |
| ~~ Age |  | UK: -17.44^***^ (4.88) Japan: -4.68 (5.03) | -.17 | -.04 |
| Size ~~ RMS |  | UK: .14^**^ (.05)  Japan: .14^***^ (.04) | .14 | .24 |
| ~~ Age |  | UK: -.92^**^ (.29)  Japan: -.15 (.21) | -.13 | -.03 |
| Relational Mobility ~~ Age |  | UK: -.02 (.12)  Japan: -.24^*^ (.12) | -.01 | -.11 |

**S7. Study 1: ANCOVA Interaction Graphs and Assumption Testing**

We conducted additional analyses to examine cross-country differences in FHQ scores, proportion of time in groups, and friendship group size, accounting for the covariates of age, gender, and socioeconomic status. We tested the assumption of homogeneity of regression slopes by examining separately the interactions between country and each of the covariates.. Age and SES were mean-centered to reduce Type I error (Schneider et al., 2015). For each of the dependent variables (FHQ scores, proportion of time in groups, and friendship group size), we ran three separate ANCOVAs. In each ANCOVA, predictors included country, age, gender, SES, and one two-way interaction between country and a demographic covariate (e.g., country and age). When an interaction term was significant, indicating a violation of the assumption of homogeneity of regression slopes, we present findings from the model including the significant interaction. When the interaction term was not significant, we report findings of an ANCOVA including the predictors of country, age, gender, and SES, with no interaction effects. Additional exploratory analyses examined relational mobility and quantity of friends as the dependent variables. For these analyses, covariates included not only age, gender, and SES, but also FHQ scores.

**FHQ Scores.** ANCOVAs revealed no significant interactions between country and gender, *F*(1, 1026) = 0.50, *p* = .479, η_p_² = .000, and between country and socioeconomic status, *F*(1, 1026) = 0.85, *p* = .358, η_p_² = .00. Interactions were therefore removed from the final analysis. Given age and socioeconomic status were significant predictors (see main article text for test statistics), we provide corresponding graphs in Figures S3a and S3b.

**Figure S3a**

*Interaction Effect of Country (Japan and UK) and Age on FHQ Scores for Study 1.*


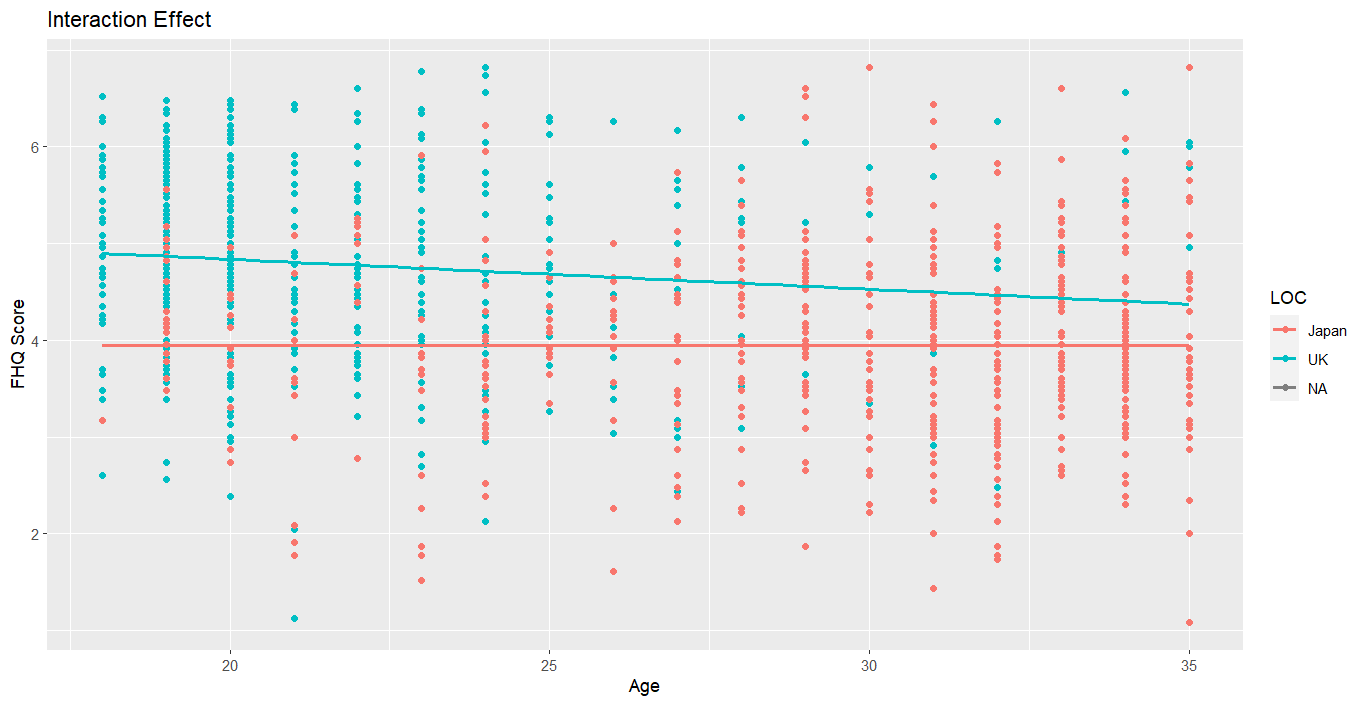


**Figure S3b**

*Relationship Between Socioeconomic Status and FHQ Scores for Study 1*


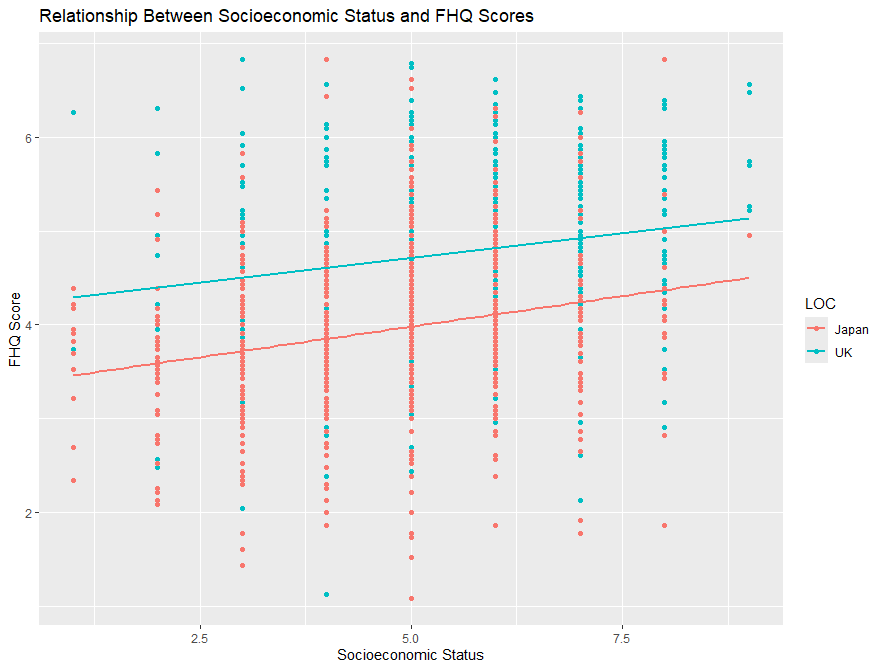


**Proportion of Time in Groups.** The ANCOVAs showed no statistically significant interactions between country and gender, *F*(1, 1008) = 0.87, *p* = .352, η_p_² = .001, and between country and socioeconomic status, *F*(1, 1008) = 0.01, *p* = .918, η_p_² = .000. Interactions were thus removed from the final analysis. Given age, socioeconomic status, and gender were significant predictors (see main article text for test statistics), we provide graphs of these in Figures S3c – S3e.

**Figure S3c**

*Interaction Effect of Country (Japan and UK) and Age on Proportion of Time in Groups for Study 1*


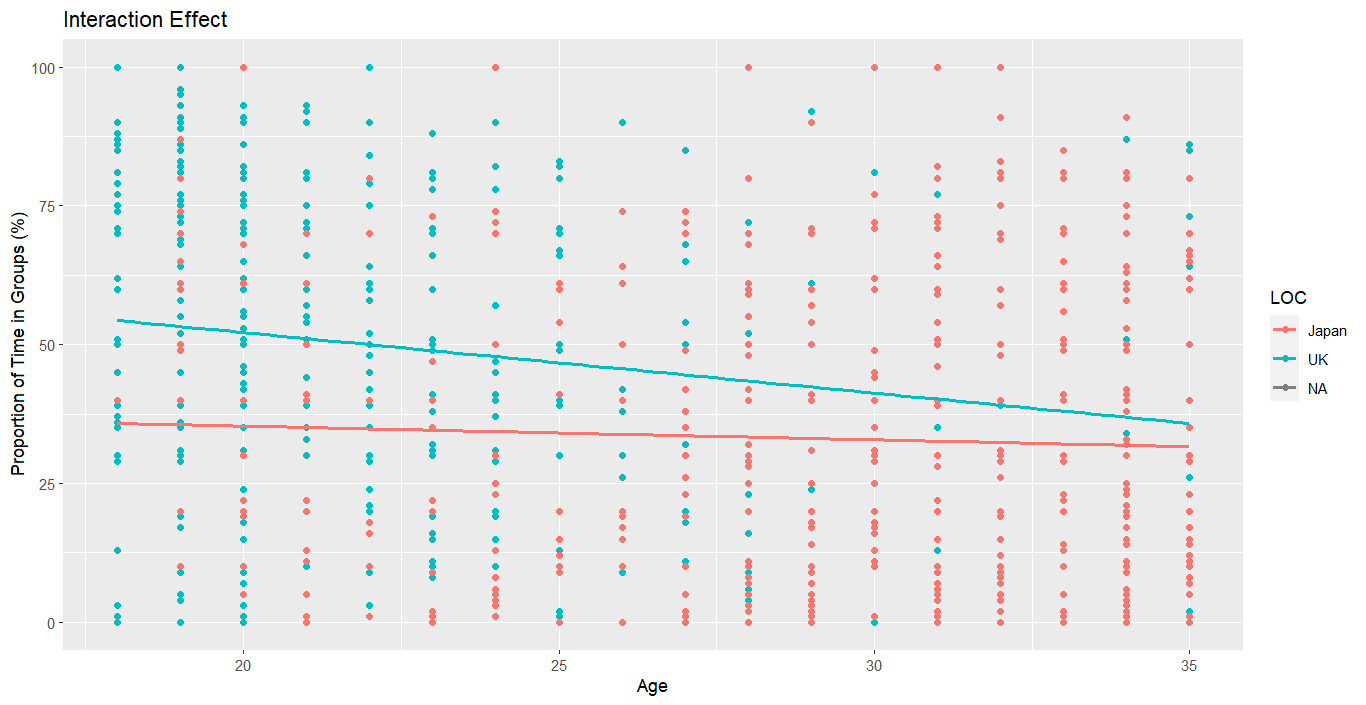


**Figure S3d**

*Relationship Between Socioeconomic Status and Proportion of Time in Groups for Study 1*


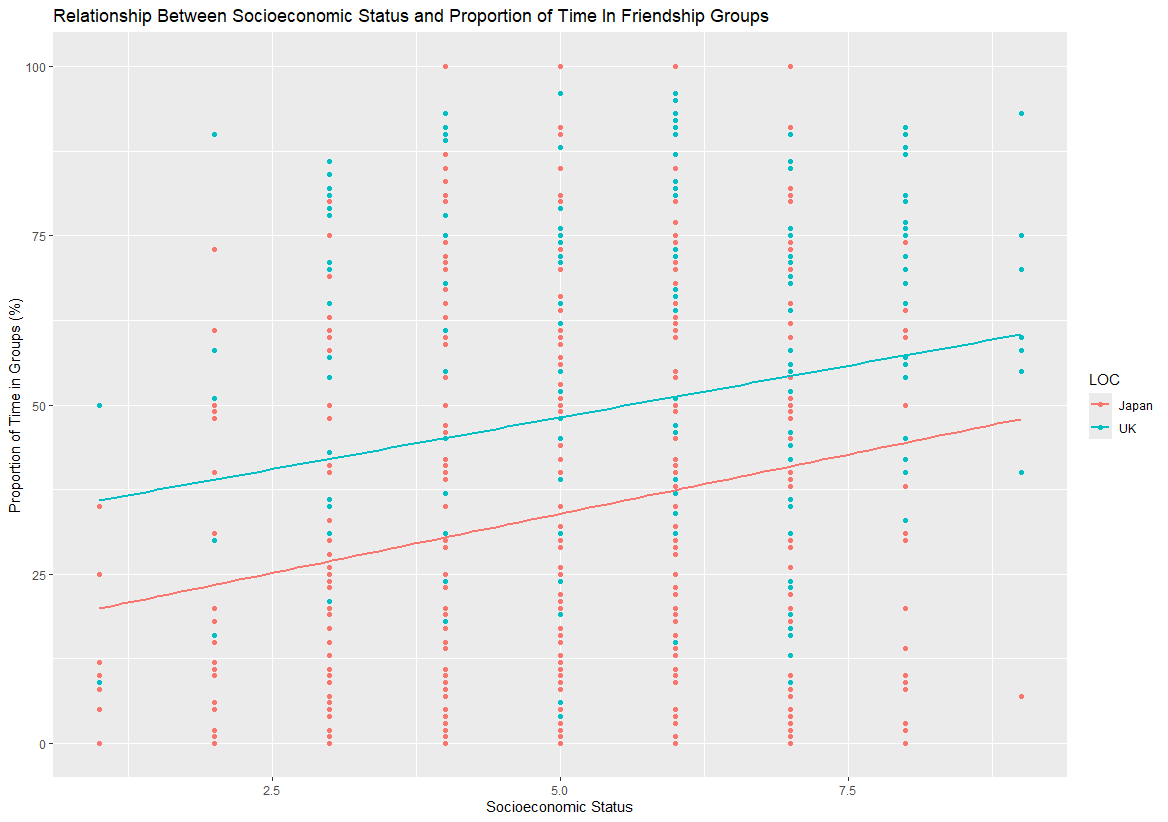


**Figure S3e**

*Proportion of Time in Groups as a Function of Gender for Study 1*


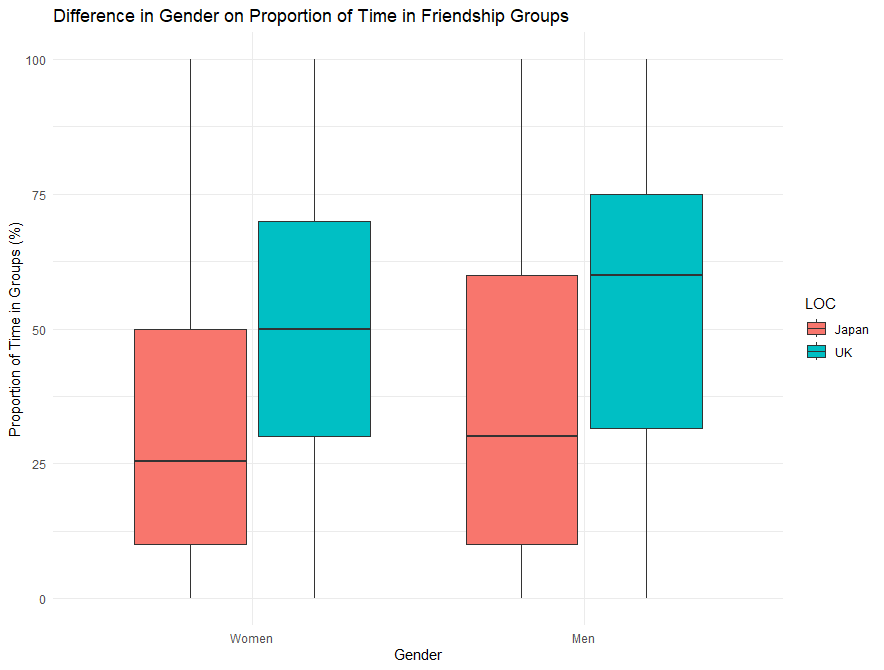


**Friendship Group Size.** ANCOVAs revealed no significant interactions between country and gender, *F*(1, 1007) = 0.46, *p* = .498, η_p_² = .000, and between country and socioeconomic status, *F*(1, 1007) = 0.04, *p* = .847, η_p_² = .000, and we removed these interactions from the final analysis. Age, socioeconomic status, and gender were significant predictors, see test statistics in the main article and graphs in Figures S3f – S3h.

**Figure S3f**

*Interaction Effect of Country (Japan and UK) and Age on Friendship Group Size for Study 1*


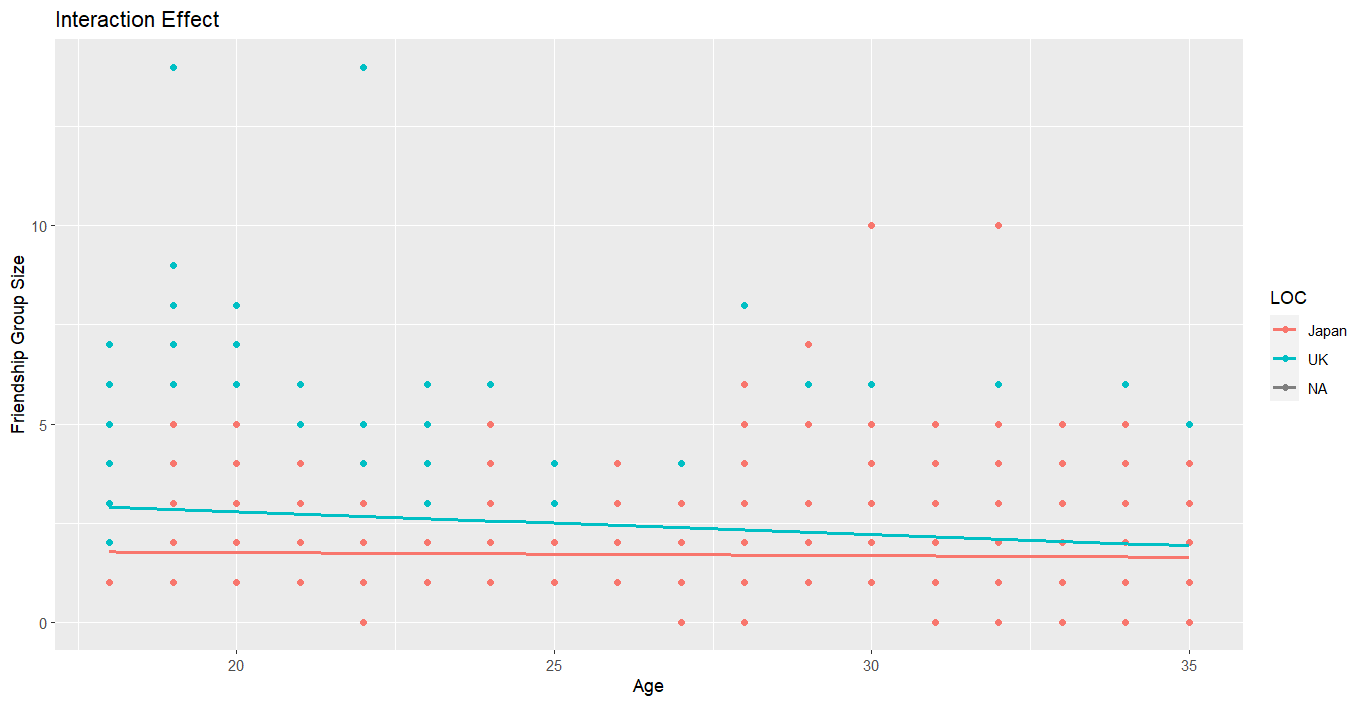


**Figure S3g**

*Relationship Between Socioeconomic Status and Friendship Group Size for Study 1*


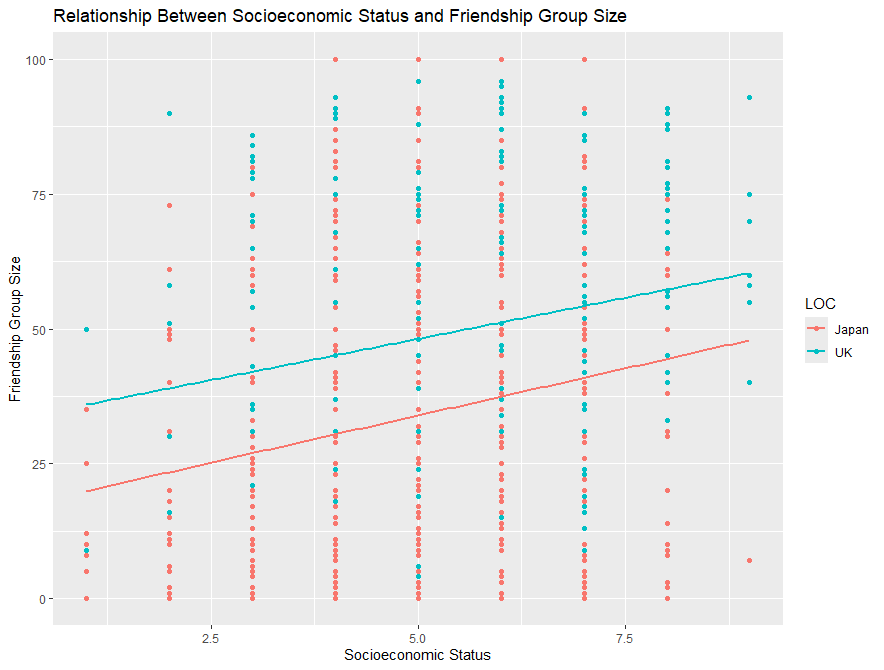


**Figure S3h**

*Friendship Group Size as a Function of Gender for Study 1*


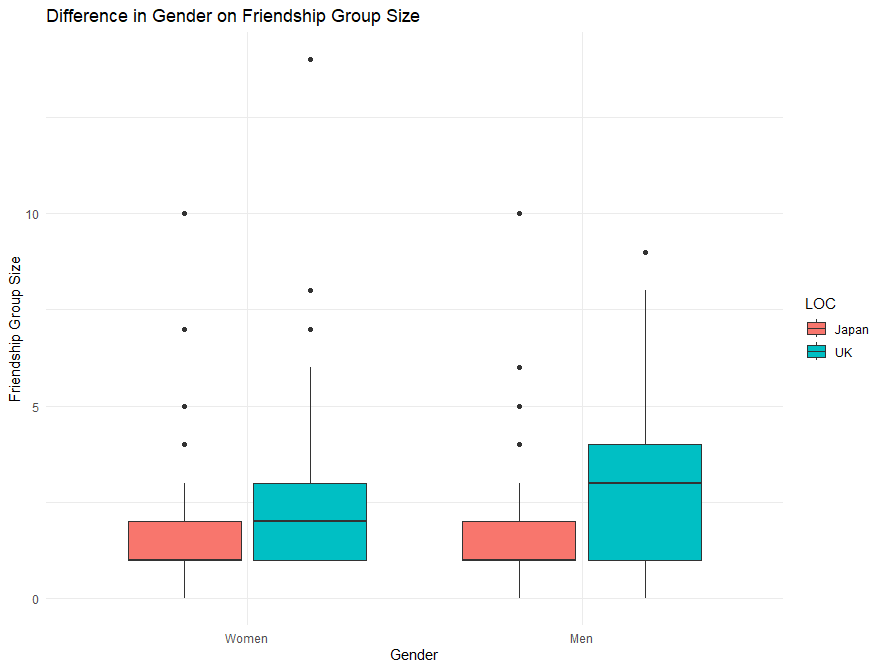


**Relational Mobility.** The following non-significant interactions in the initial ANCOVA were removed from the final analysis: Country * Age, *F*(1, 1011) = 3.29, *p* = .070, η_p_² = .003; Country * FHQ score, *F*(1, 1011) = 2.57, *p* = .109, η_p_² = .003; Country * Gender, *F*(1, 1011) = 0.02, *p* = .876, η_p_² = .000; Country * Socioeconomic status, *F*(1, 1011) = 0.00, *p* = .949, η_p_² = .000. Socioeconomic status and FHQ scores were significant predictors, and we provide graphs of these relationships in Figures S3i and S3j.

**Figure S3i**

*Relationship Between Socioeconomic Status and Relational Mobility for Study 1*


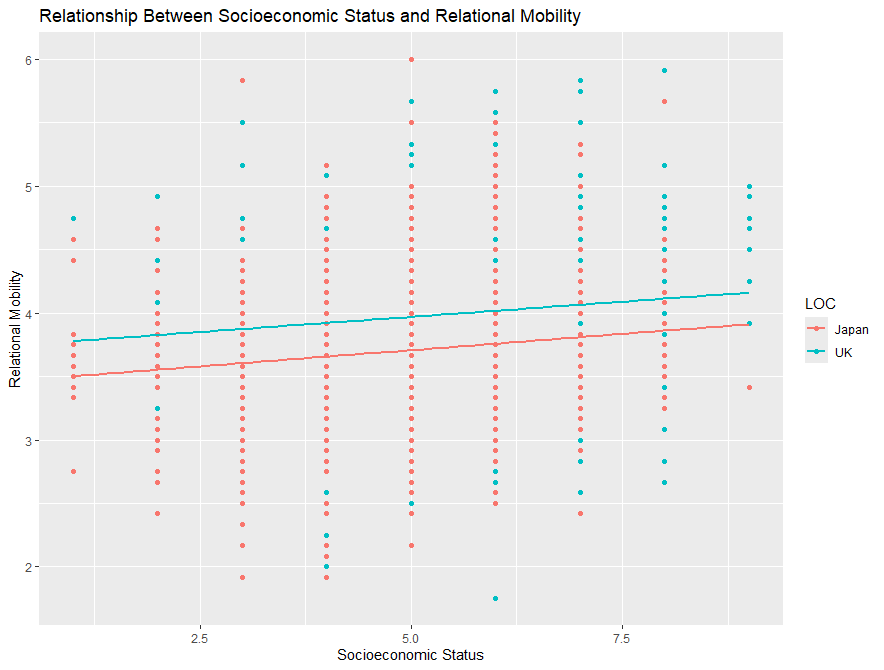


**Figure S3j**

*Relationship Between Socioeconomic Status and FHQ Scores for Study 1*


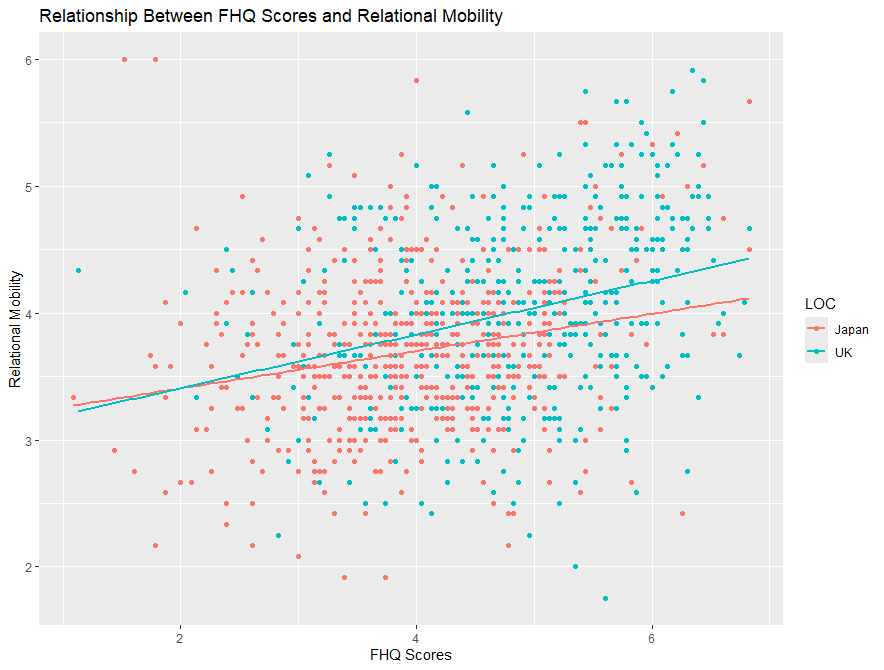


**Friendship Quantity.** The following non-significant interactions in the initial ANCOVA were removed from the final analysis: Country * Age, *F*(1, 1025) = 0.67, *p* = .413, η_p_² = .001; η_p_² = .003; Country * Gender, *F*(1, 1025) = 0.80, *p* = .372, η_p_² = .001; Country * Socioeconomic status, *F*(1, 1025) = 0.69, *p* = .406, η_p_² = .001. FHQ scores were a significant predictor of friendship quantity, see Figure S3k.

**Figure S3k**

*Interaction between FHQ Scores and Friendship Quantity for Study 1*


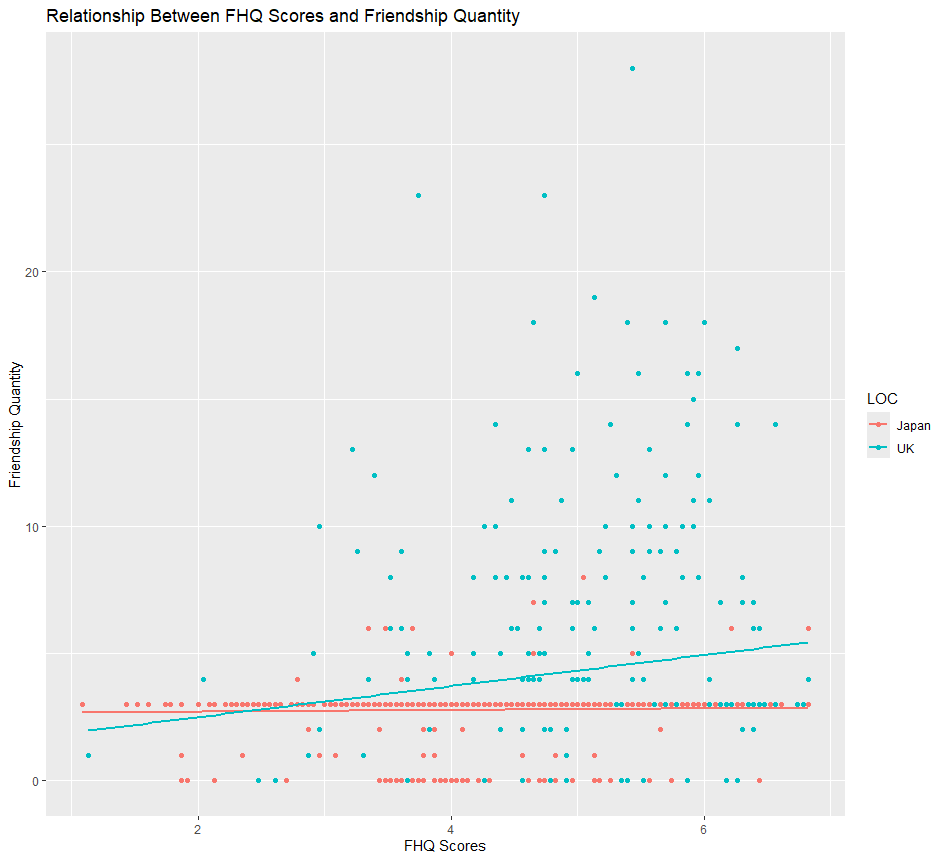


**S8. Study 2: Demographic Testing**

The female-male gender split was different across countries, χ² (1, *N* = 623) = 42.50, *p* <.001, but women and men did not significantly differ in their FHQ scores, *t*(621) = 1.43, *p* = .156, proportion of time in groups, *t*(616) = -1.47, *p* = .143, or friendship group size, *t*(616) = .12, *p* = .907. Japanese respondents rated themselves as lower on socioeconomic status (*M* = 5.06, *SD* = 1.58) than respondents from the UK (*M* = 5.87, *SD* = 1.61), *t* (627) = -6.33, *p* < .001. *d* = -.50. The age gap between Japan and the UK was smaller than in Study 1, but still statistically significant, Welch’s t-test *t*(394.47) = 13.77, *p* < .001, *d* = 1.11.

**S9. Study 2: Self-reported Friendship Group Sizes and FHQ Scores**

Similar to Study 1, we examined participants’ numeric responses of estimated friendship group size. Sizes of friendship groups were transformed them into a 4-level categorical variable (1 friend or dyadic interaction, 2 friends, 3 friends, 4 or more friends). We then used it as an independent variable and investigated how FHQ scores differed by group size. Figure S4 shows the distribution of the FHQ scores as a function of friendship group size. The interaction between country and friendship group size was not significant, *F*(3, 613) = 0.20, *p* = .894, η_p_² = .001 and was removed from the final analysis.

**Figure S4**

*Number of Friends in a Friendship Group as a Function of FHQ scores*


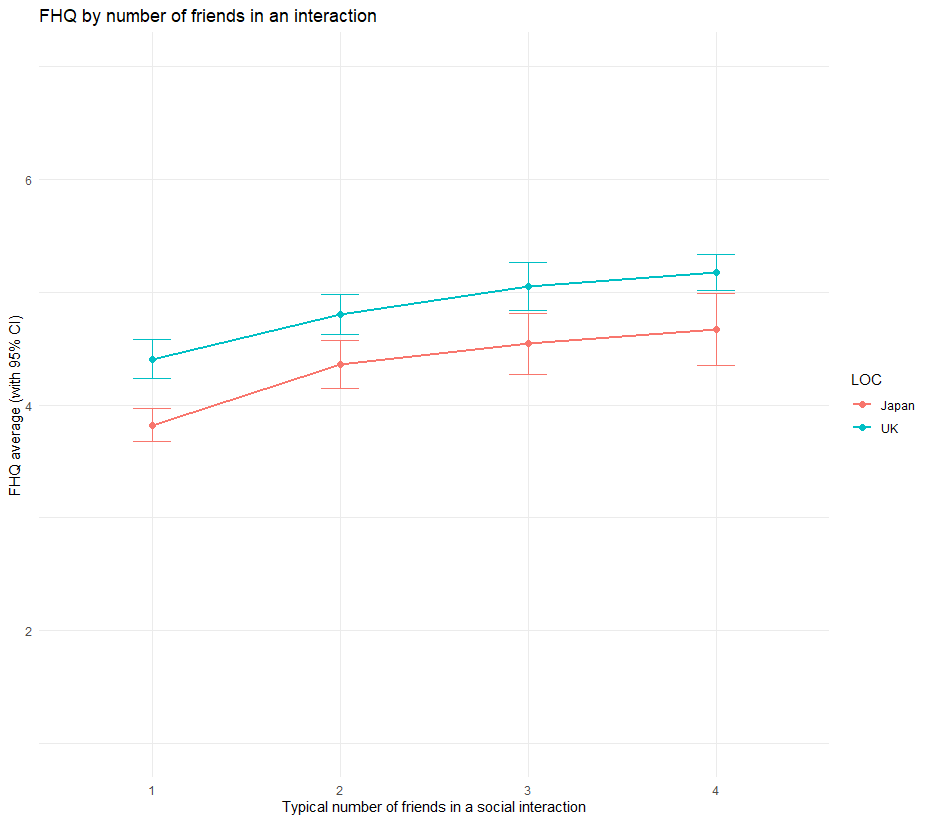


The ANOVA showed that friendship group size was a significant predictor of FHQ scores, *F*(3, 617) = 41.81, *p* < .001, η_p_² = .169. Post-hoc analyses with Bonferroni correction found that people who socialized in dyads were significantly lower on FHQ score than all other group sizes (all *p* < .001). People who socialized with two friends at a time also differed from those with 4 friends at a time (*p* < .001). People who socialized with two friends were not significantly different from people who socialized with three friends (*p* = .123) and people who socialized with three friends were not significantly different from people who socialized with four friends (*p* = .647). Overall, this suggests that FHQ scores can distinguish between people who socialize in dyads (one friend at a time) versus groups. However, group sizes differing on only one person may be relatively similar.

**S10. Study 2: ANCOVA Interaction Graphs and Assumption Testing**

We examined cross-country differences in FHQ scores, proportion of time in friendship groups, and friendship group sizes accounting for the covariates of age, gender, and socioeconomic status. We first tested the assumption of homogeneity of regression slopes examining the interactions between country and each of the three covariates separately. Similar exploratory analyses examined the effects of country and covariates on relational mobility and attachment styles. For these analyses, covariates included not only age, gender, and SES, but also FHQ scores.

**FHQ Scores.** The following non-significant interactions in the initial ANCOVAs were removed from the final analysis: Country * Age, *F*(1, 617) = 0.56, *p* = .455, η_p_² = .001; Country * Gender, *F*(1, 617) = 0.51, *p* = .474, η_p_² = .001; and Country * Socioeconomic status, *F*(1, 617) = 0.01, *p* = .928, η_p_² = .000. The relationship of significant covariates and FHQ scores can be found in Figures S5a-S5b and test statistics are reported in the main article.

**Figure S5a**

*Relationship Between Age and FHQ Scores for Study 2*


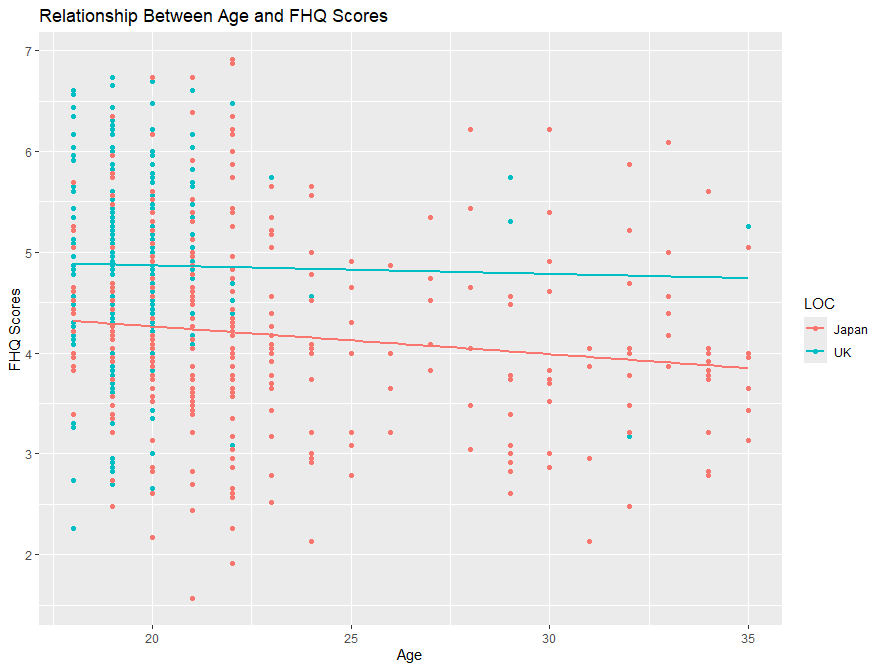


**Figure S5b**

*Relationship Between Socioeconomic Status and FHQ Scores for Study 2*


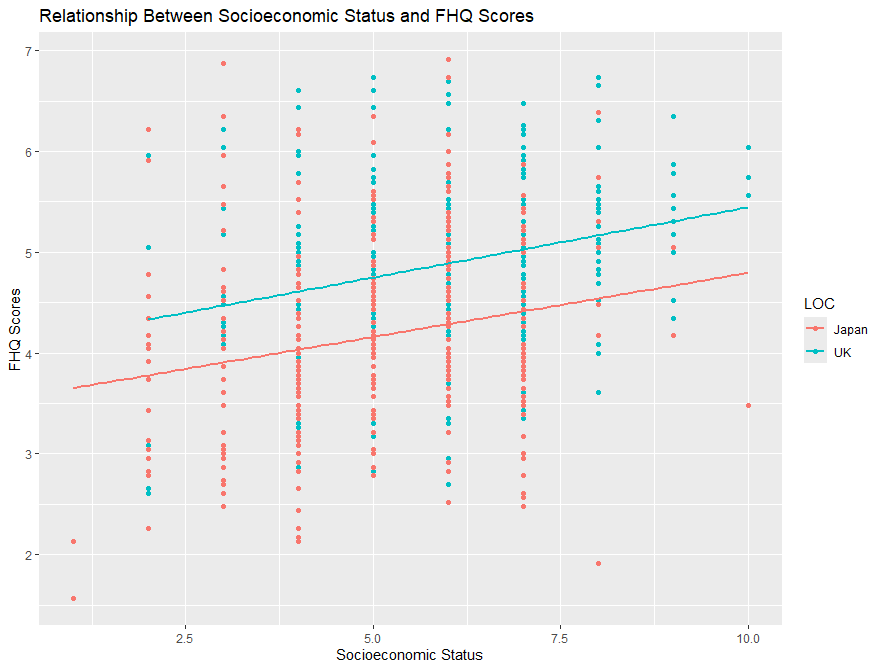


**Proportion of Time in Groups.** The following non-significant interactions in the initial ANCOVAs were removed from the final analysis: Country * Age, *F*(1, 612) = 0.46, *p* = .496, η_p_² = .001; Country * Gender, *F*(1, 612) = 2.37, *p* = .124, η_p_² = .004; and Country * Socioeconomic status, *F*(1, 612) = 0.02, *p* = .889, η_p_² = .000. The relationship of significant covariates and proportion of time in groups can be found in Figures S5c-S5d and test statistics are reported in the main article.

**Figure S5c**

*Graph Showing the Relationship Between Socioeconomic Status and Proportion of Time in Friendship Groups for Study 2.*


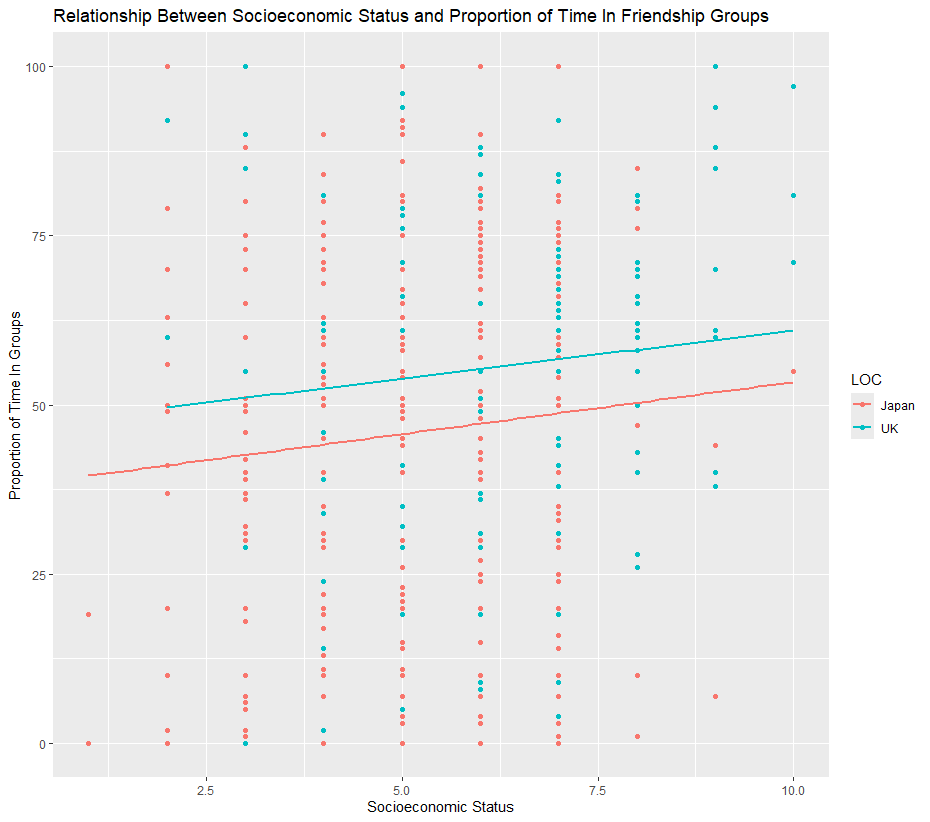


**Figure S5d**

*Proportion of Time in Friendship Groups as a Function of Gender for Study 2*


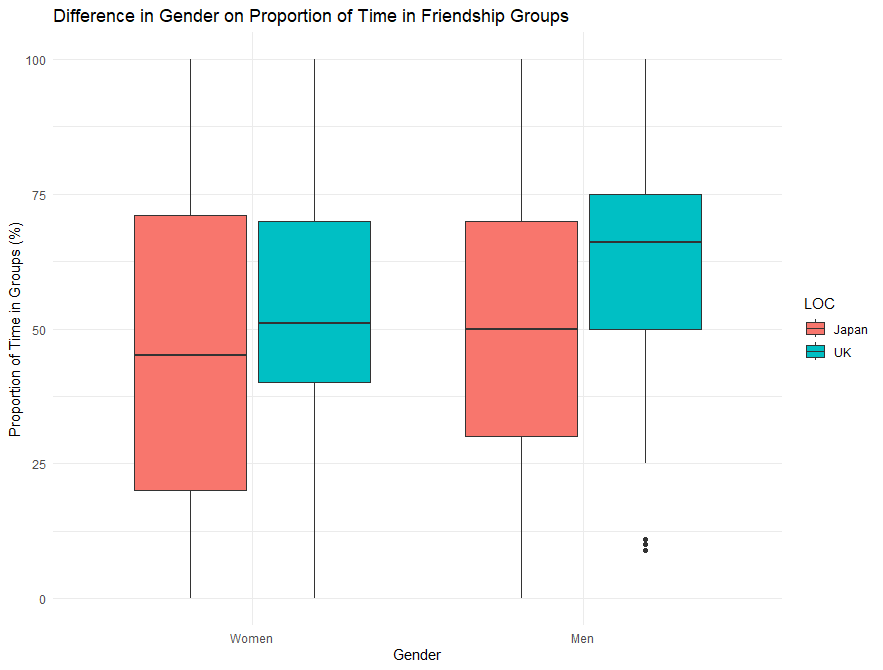


**Friendship Group Size.** The following non-significant interactions in the initial ANCOVAs were removed from the final analysis: Country * Age, *F*(1, 612) = 1.93, *p* = .165, η_p_² = .003; Country * Gender, *F*(1, 612) = 1.57, *p* = .210, η_p_² = .003; and Country * Socioeconomic status, *F*(1, 612) = 0.62, *p* = .430, η_p_² = .001. The relationship of significant covariates and friendship group size can be found in Figures S5f-S5e (see main text for test statistics).

**Figure S5e**

*Graph Showing the Relationship Between Socioeconomic Status and Friendship Group Size for Study 2*


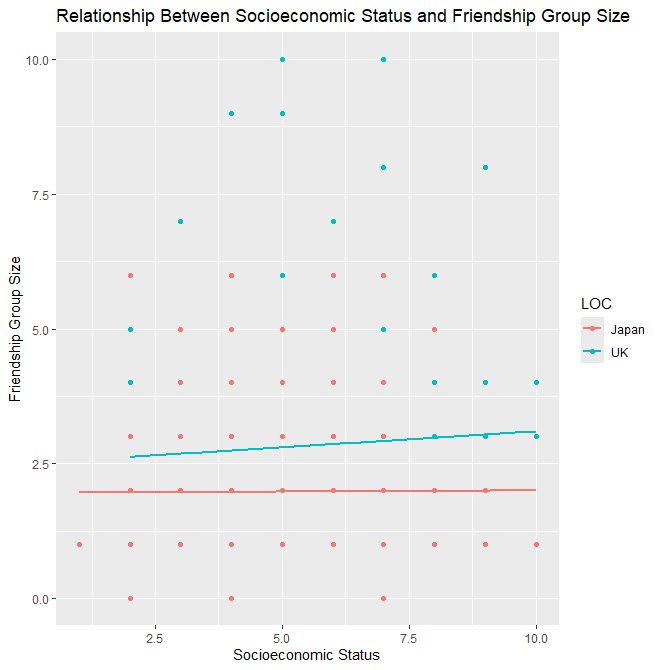


**Figure S5f**

*Friendship Group Size as a Function of Gender for Study 2*


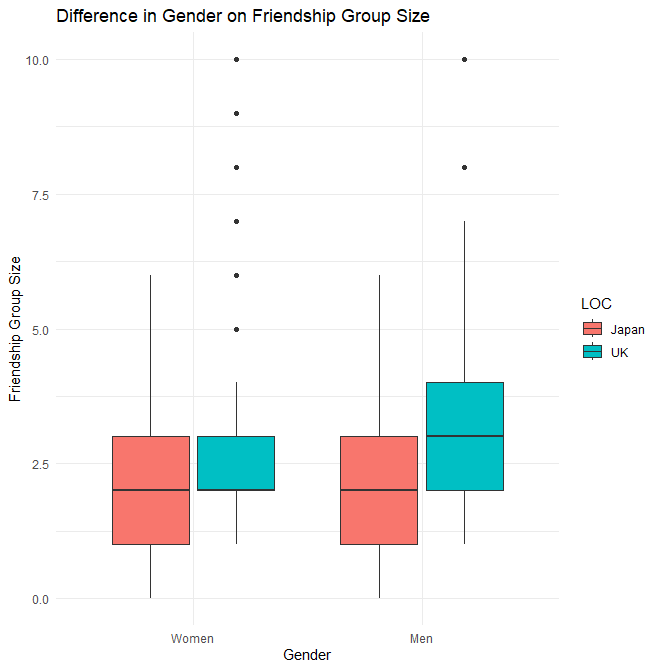


**Relational Mobility.** The following non-significant interactions in the initial ANCOVAs were removed from the final analysis: Country * Age, *F*(1, 610) = 0.05, *p* = .819, η_p_² = .000; Country * FHQ score, *F*(1, 610) = 0.39, *p* = .531, η_p_² = .001; Country * Gender, *F*(1, 610) = 0.09, *p* = .762, η_p_² = .000; and Country * Socioeconomic status, *F*(1, 610) = 1.59, *p* = .208, η_p_² = .003. Figures S5g-S5j display the relationships between significant covariates and relational mobility. Main effects of country are reported in the main text, General Discussion.

**Figure S5g**

*Relationship Between Socioeconomic Status and Relational Mobility for Study 2*


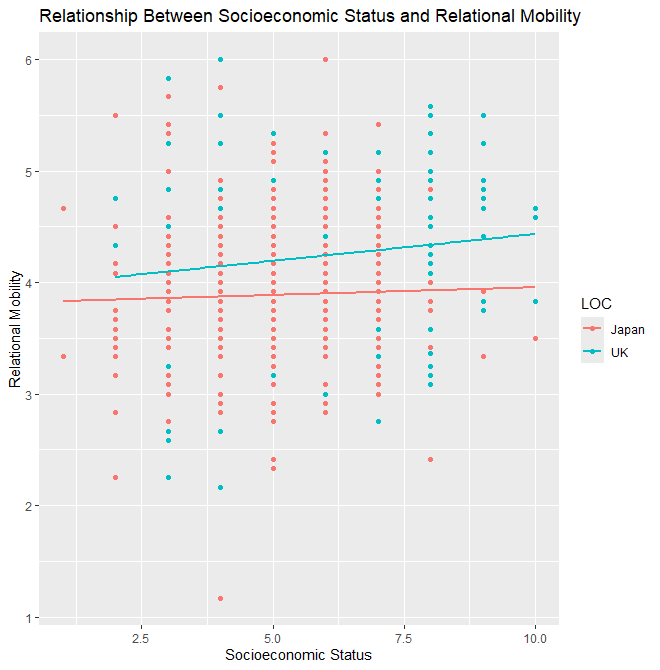


**Figure S5h**

*Relationship Between Relational Mobility and FHQ Scores for Study 2*


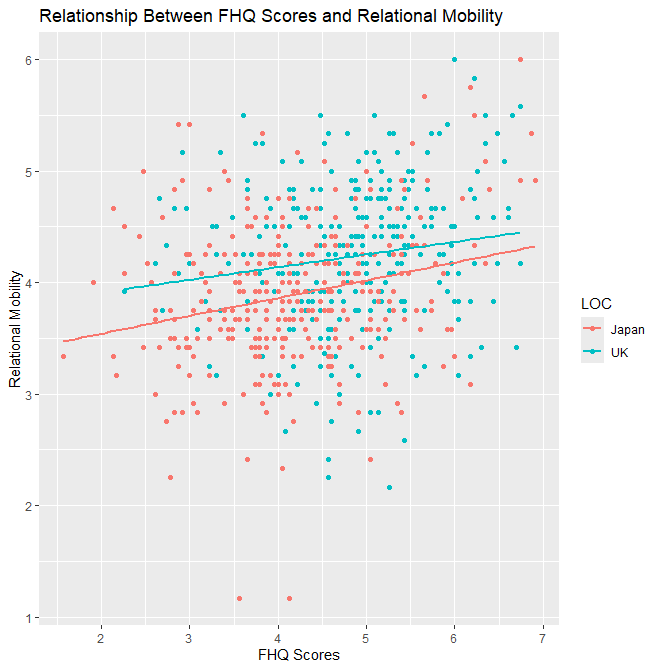


**Figure S5i**

*Relationship Between Age and Relational Mobility for Study 2*


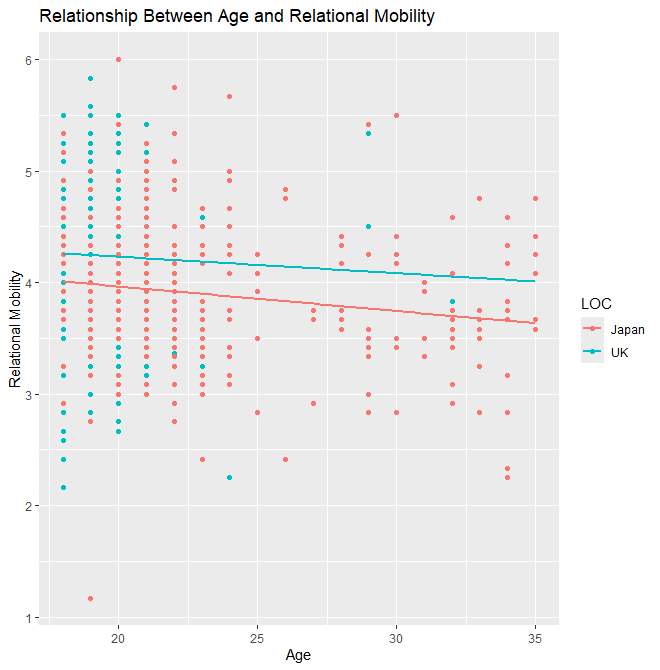


**Figure S5j**

*Relationship Between Gender and Relational Mobility for Study 2*


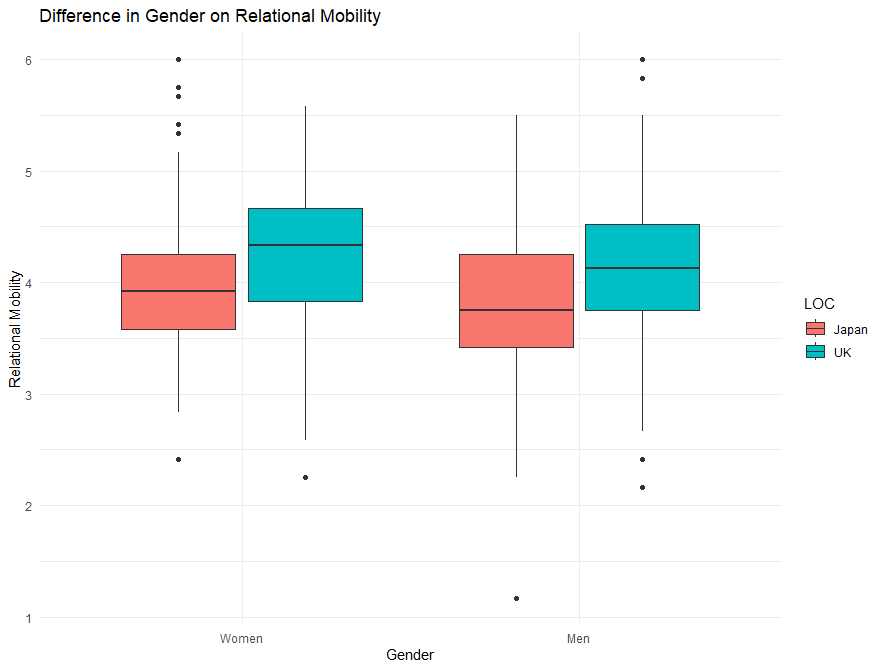


**Avoidant Attachment.** The following non-significant interactions in the initial ANCOVAs were removed from the final analysis: Country * Age, *F*(1, 609) = 0.56, *p* = .455, η_p_² = .001; Country * FHQ score, *F*(1, 609) = 1.34, *p* = .247, η_p_² = .002; Country * Gender, *F*(1, 609) = 1.17, *p* = .279, η_p_² = .002; and Country * Socioeconomic status, *F*(1, 609) = 0.11, *p* = .738, η_p_² = .000. In an ANCOVA controlling for covariates of age, gender, SES, and FHQ, the difference in avoidant attachment between countries became significant, *F*(1, 610) = 6.61, *p* =.010, η_p_² = .011. FHQ scores, *F*(1, 610) = 72.10, *p* < .001, η_p_² = .106, and socioeconomic status were significant covariates, *F*(1, 610) = 3.89, *p* = .049, η_p_² = .006. Age, *F*(1, 610) = 0.16, *p* = .686, η_p_² = .000, and gender, *F*(1, 610) = 0.06, *p* = .804, η_p_² = .000, were not significant covariates. Figures S5k-S5l display the relationships between avoidant attachment and FHQ as well as SES.

**Figure S5k**

*Relationship Between Avoidant Attachment and FHQ Scores for Study 2*


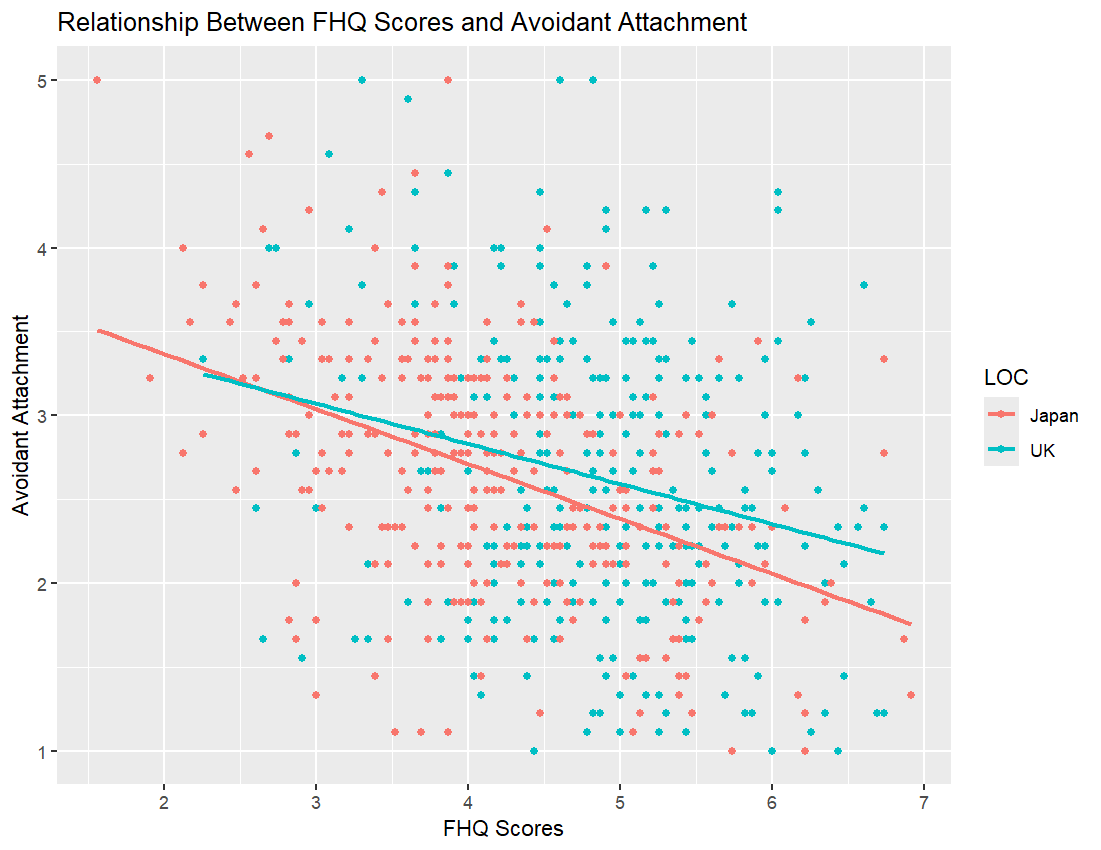


**Figure S5l**

*Relationship Between Avoidant Attachment and Socioeconomic Status for Study 2*


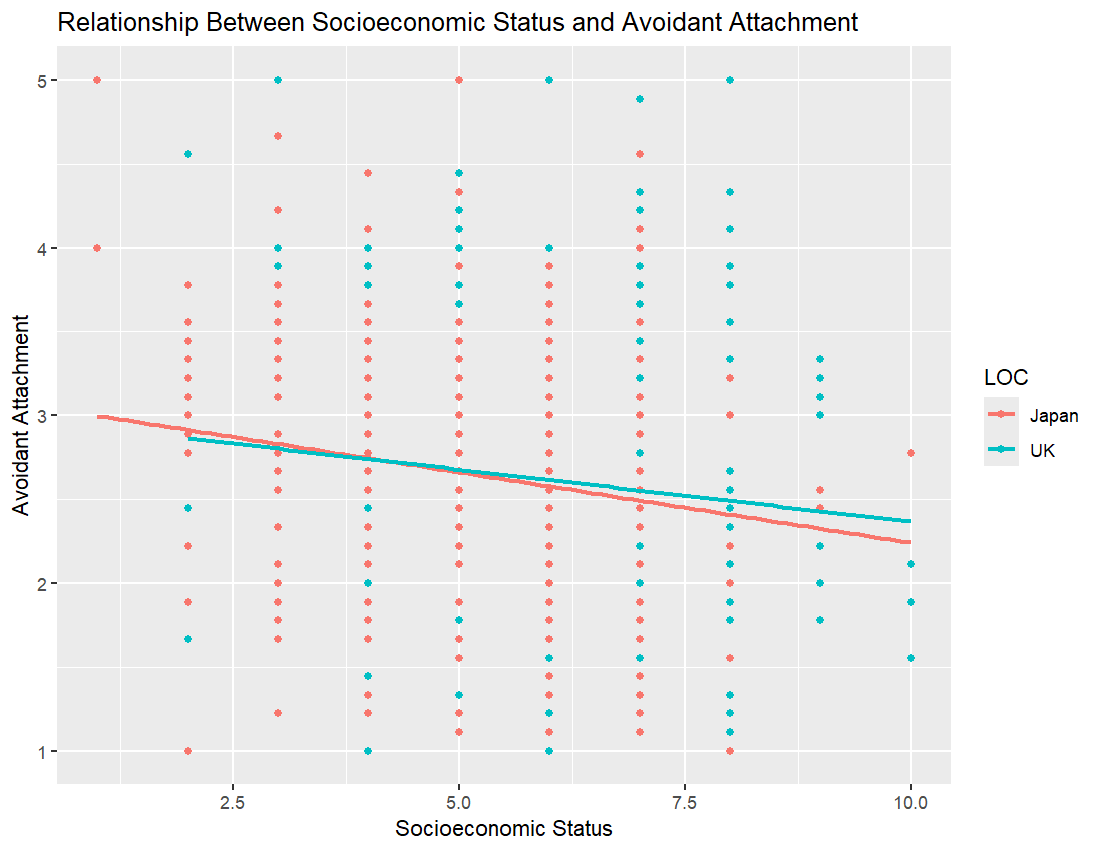


**Anxious Attachment.** The following non-significant interactions in the initial ANCOVAs were removed from the final analysis: Country * Age, *F*(1, 609) = 0.14, *p* = .709, η_p_² = .000; Country * FHQ score, *F*(1, 609) = 0.19, *p* = .663, η_p_² = .000; Country * Gender, *F*(1, 609) = 2.19, *p* = .139, η_p_² = .004; and Country * Socioeconomic status, *F*(1, 609) = 3.77, *p* = .053, η_p_² = .006. In an ANCOVA, the difference in anxious attachment between countries remained non-significant, *F*(1, 610) = 0.66, *p* =.419, η_p_² = .001, when controlling for covariates. Socioeconomic status was a significant covariate, *F*(1, 610) = 15.44, *p* < .001, η_p_² = .025. FHQ scores, *F*(1, 610) = 2.15, *p* = .143, η_p_² = .004, age, *F*(1, 610) = 2.48, *p* = .116, η_p_² = .004, and gender, *F*(1, 610) = 0.86, *p* = .354, η_p_² = .001, were not significant covariates. The relationship between socioeconomic status and anxious attachment is shown in Figure S5m.

**Figure S5m**

*Relationship Between Anxious Attachment and Socioeconomic Status for Study 2*


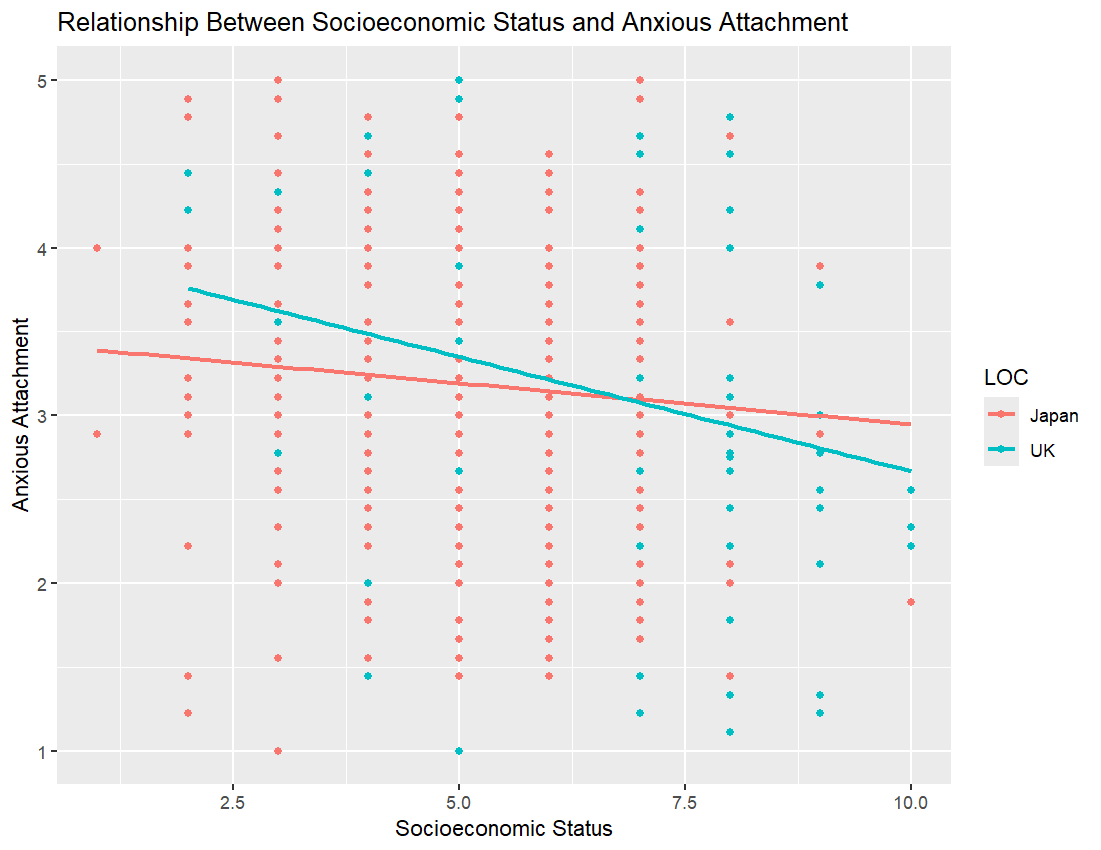


**S11. Study 2: Attachment Styles: Supplementary Analyses**

To further investigate the relationships between attachment and friendship styles, we conducted a series of supplementary analyses. First, a regression analysis with FHQ scores as the dependent variable examined the effects of avoidant and anxious attachment styles, as well as their interaction. Increases in avoidant attachment were associated with decreases in FHQ scores, *B* = -0.40,

$\beta$ = -0.31, *SE* = 0.05, *p* < .001. There was no significant effect of anxious attachment, *B* <.01, $\beta$ < .01, *SE* = 0.05, *p* = 0.957 and the interaction was also not significant, *B* = 0.03, $\beta$ < 0.03, *SE* - 0.05, *p* = 0.474.

A second regression analysis examined the joint effects of country, avoidant and anxious attachment styles, as well as the interaction between attachment styles. Increases in avoidant attachment were associated with decreases in FHQ scores, *B* = -0.38, $\beta$ = -0.31, *SE* = 0.05, *p* < .001. Participants from the UK had higher FHQ scores than Japan, *B* = 0.69, *SE* = 0.07, *p* < .001. There was no significant effect of anxious attachment, *B* = -0.02, $\beta$ = -0.01, *SE* = 0.04, *p* = 0.711, and the interaction effect was also not significant, *B* = 0.02, $\beta$ = 0.02, SE - 0.05, *p* = 0.623.

We then conducted an ANCOVA with FHQ scores as the outcome variable. Country was included as a between participants variable, and avoidant and anxious attachment styles, as well as their interaction, as covariates. There was a significant effect of country on friendship styles, *F*(1, 617) = 99.82, *p* < .1001, η_p_² = .139, suggesting the UK scored higher on group-oriented friendship style than Japan. In addition, avoidant attachment predicted FHQ scores, *F*(1, 617) = 270.16, *p* < .001, η_p_² = .102, suggesting that increases in avoidant attachment were associated with decreases in group-oriented friendship. There was no significant effect of anxious attachment, *F*(1, 617) = 0.14, *p* = .711, η_p_² = .000. The interaction between avoidant and anxious attachment was also not significant, *F*(1, 617) = 0.24, *p* = .623, η_p_² = .000.

Finally, we conducted a mixed ANOVA with avoidant and anxious attachment styles as within-participants variable and country as between-participants variable. Levels of avoidant attachment were significantly lower than levels of anxious attachment, *F*(1, 620) = 199.29, *p* < .001, η_p_² = .243. There was no significant main effect of country, *F*(1, 620) = .00, *p* = 972, η_p_² = .000, and the interaction between attachment styles and country was also not significant, *F*(1, 620) = 0.80, *p* = .371, η_p_² = .001.

**S12. Study 2: Alternative Hypothesis Testing with the Combined Friendship Habits and Relational Mobility Models in Study 2**

To test hypotheses in Study 2, we employed the measurement invariance models derived from Study 1. We therefore included the second-order partial scalar model of the FHQ with four first-order dimensions (extraversion, intimacy, positive group identification and negative identification) which loaded onto a second-order factor, known as friendship styles, with the modified edits through measurement invariance (GP4 factor loading varied; E7, I5, GP1, GN1 & GN2 intercept varied). We also included the second-order partial scalar model of the RMS with two first-order dimensions (meeting and choosing) with four covariances which loaded onto a second-order factor, called relational mobility, with the modified edits through measurement invariance (RMS9 intercept varied). We kept the common bias factors controlling for response style separate for both FHQ and RMS. In addition to the measurement invariance models, we included the size of friendship groups, proportion of time in groups, and age as indicator-level variables. We then used this new model to analyze correlations between constructs and country-level differences in friendship styles and relational mobility.

The data had high kurtosis and skewness as evidenced by a Mardia’s test. We thus used a robust maximum likelihood estimator [MLR] and provide robust indices for χ², CFI, TLI and RMSEA. The fit of this new structural equation model was good for RMSEA and SRMR and borderline acceptable for CFI and TLI (see Table S5a). Although model fit was less good than for the equivalent model tested Study 1, it is worth remembering that Study 2 had a lower sample size (with approximately 300 participants per country, compared to approximately 500 participants per country in Study 1). An inspection of the chi-square separately for the two countries suggest that the model had a less good fit in Japan, χ² = 1298.19, than in the UK, χ² = 1172.31. Model intercepts can be found in Table S5b. Unlike in Study 1, this model did produce a lavaan warning that the model’s covariance-variance matrix might not be positive definite.

**Table S5a**

*Study 2: Model Fit Indices for the Hypothesis Testing Structural Equation Model for the Overall Sample*

| Model | χ² | df | CFI | TLI | SRMR | RMSEA | RMSEA 90% CI |  |
| --- | --- | --- | --- | --- | --- | --- | --- | --- |
| SEM | 2470.50^***^ | 1350 | .880 | .875 | .073 | .055 | (.052, .059) |  |
| *^***^p < .001.* | | | | | | | | |

**Table S5b**

*Study 2: Intercepts for the Hypothesis Testing Structural Equation Model*

| Factor | Item |  | Standardized Intercept | |
| --- | --- | --- | --- | --- |
|  |  | Intercept | UK | Japan |
| Extraversion | E1 | 4.44^***^ (.10) | 2.50 | 2.58 |
|  | E2 | 4.34^***^ (.10) | 2.48 | 2.58 |
|  | E3 | 3.78^***^ (.10) | 2.21 | 2.33 |
|  | E4 | 4.47^***^ (.09) | 2.82 | 2.82 |
|  | E5 | 3.76^***^ (.10) | 2.13 | 2.24 |
|  | E6 | 4.11^***^ (.07) | 2.45 | 2.52 |
|  | E7 | UK: 3.30^***^ (.09)  Japan: 3.88^***^ (.11) | 1.89 | 2.50 |
|  | E8 | 4.35^***^ (.09) | 2.66 | 2.69 |
| Intimacy | I1 | 5.33^***^ (.07) | 3.98 | 3.62 |
|  | I2 | 5.34^***^ (.07) | 4.05 | 3.28 |
|  | I3 | 5.53^***^ (.07) | 3.98 | 3.53 |
|  | I4 | 5.51^***^ (.08) | 4.14 | 3.34 |
|  | I5 | UK: 5.74^***^ (.07)  Japan: 6.19^***^ (.12) | 4.83 | 4.44 |
|  | I6 | 4.95^***^ (.07) | 3.35 | 3.08 |
| Positive Group Identification | GP1 | UK: 6.01^***^ (.06)  Japan: 5.45^***^ (.09) | 5.49 | 4.05 |
|  | GP2 | 5.33^***^ (.09) | 3.71 | 3.55 |
|  | GP3 | 5.23^***^ (.09) | 3.64 | 3.32 |
|  | GP4 | 5.45^***^ (.06) | 4.54 | 3.99 |
|  | GP5 | 4.84^***^ (.09) | 3.10 | 3.09 |
| Negative Group Identification | GN1 | UK: 4.93^***^ (.09)  Japan: 4.10^***^ (.13) | 3.43 | 2.51 |
|  | GN2 | UK: 5.48^***^ (.09)  Japan: 4.26^***^ (.12) | 3.61 | 2.43 |
|  | GN3 | 4.77^***^ (.09) | 3.21 | 3.02 |
|  | GN4 | 4.85^***^ (.08) | 3.26 | 3.25 |
| Friendship Styles | Extra | .17^**^ (.08) |  | .12 |
|  | Intimacy | -.72^***^ (.08) |  | -.82 |
|  | Positive Group I | -.13 (.06) |  | -.17 |
|  | Negative Group I | .28^***^ (.09) |  | .26 |
| Friendship Styles | FHQ | -.54^***^ (.07) |  | -.51 |
| Common Bias Factor FHQ | Style1 | -.19^***^ (.04) |  | -.53 |
| Meeting | RMS1 | 4.68^***^ (.06) | 4.61 | 3.91 |
|  | RMS2 | 4.44^***^ (.06) | 3.95 | 3.91 |
|  | RMS4 | 4.43^***^  (.07) | 3.39 | 3.49 |
|  | RMS5 | 4.35^***^  (.06) | 3.78 | 3.54 |
|  | RMS8 | 4.37^***^ (.06) | 4.06 | 3.60 |
| Choosing | RMS3 | 4.68^***^ (.05) | 4.69 | 4.44 |
|  | RMS6 | 4.01^***^  (.05) | 3.61 | 3.32 |
|  | RMS7 | 4.23^***^  (.06) | 3.75 | 3.49 |
|  | RMS9 | UK: 3.10^***^ (.06) Japan: 3.72^***^ (.08) | 2.76 | 3.59 |
|  | RMS10 | 4.54^***^ (.05) | 4.54 | 4.44 |
|  | RMS11 | 4.06^***^ (.06) | 3.57 | 3.58 |
|  | RMS12 | 4.08^***^ (.07) | 3.57 | 3.50 |
| Relational Mobility | Meeting | -.17^**^ (.05) |  | -.20 |
|  | Choosing | -.12^**^ (.04) |  | -.20 |
| Relational Mobility | RMS | -.28^***^ (.04) |  | -.56 |
| Common Bias Factor RMS | Style2 | -.02 (.03) |  | -.07 |
| Friendship Group Size | Size | 2.83^***^ (.10) | 1.65 | 2.21 |
| Friendship Group Size | Overall | -.85^***^ (.12) |  | -.69 |
| Proportion Time in Groups | Prop | 54.75^***^ (1.34) | 2.37 | 1.90 |
| Proportion Time in Groups | Overall | -8.99^***^ (2.05) |  | -.33 |
| Age | Age | 19.60^***^ (.10) | 11.47 | 5.05 |
| Age | Overall | 3.87^***^ (.28) |  | .84 |
| Covariances |  |  |  |  |
| RMS2 ~~ RMS5 |  | UK: .48^***^ (.08) Japan: .06 (.07) | .60 | .08 |
| RMS11 ~~ RMS12 |  | UK: .42^***^ (.07) Japan: .37^***^ (.09) | .55 | .53 |
| Friendship Habits ~~ Proportion |  | UK: 9.25^***^ (1.61) Japan: 15.54^***^ (2.04) | .42 | .54 |
| ~~ Size |  | UK: .55^***^ (.12)  Japan: .44^***^ (.08) | .34 | .34 |
| ~~ RMS |  | UK: .12^**^ (.04)  Japan: .20^***^ (.05) | .24 | .37 |
| ~~ Age |  | UK: -.05 (.12)  Japan: -.72^**^ (.27) | -.03 | -.15 |
| Proportion ~~ Size |  | UK: 18.07^***^ (2.41) Japan: 17.65^***^ (1.79) | .46 | .53 |
| ~~ RMS |  | UK: 2.06^*^ (.95)  Japan: 1.65 (1.00) | .17 | .12 |
| ~~ Age |  | UK: .48 (2.37)  Japan: -5.30 (6.88) | .01 | -.04 |
| Size ~~ RMS |  | UK: .07 (.06)  Japan: .10 (.05) | .08 | .17 |
| ~~ Age |  | UK: -.32^*^ (.14)  Japan: -.64^*^ (.32) | -.11 | -.11 |
| Relational Mobility ~~ Age |  | UK: -.04 (.07)  Japan: -.39^*^ (.18) | -.04 | -.17 |

**S13. Study 2: Measurement Invariance for Anxious and Avoidant Attachment**

The 18 items of the Experiences in Close Relationships (ECR-S; Fraley et al., 2000) are presented in Table S6a. Items are designated with a code (e.g., ECR1, ECR2 etc.) that we will use for the rest of the supplementary materials. All 18 items for anxious and avoidant attachment were retained throughout the measurement invariance process.

**Table S6a**

*Items and Dimensions of the Experiences in Close Relationships Scale (ECR-S; Fraley et al., 2000)*

| Anxious |
| --- |
| ECR1 I’m afraid I will lose my partner’s love. |
| ECR3 I often worry about my partner will not want to stay with me |
| ECR5 I often worry that my partner does not love me. |
| ECR7 I worry that romantic partners won’t care about me as I care about them |
| ECR9 I often wish that my partner’s feelings for me were as strong as mine for him or her |
| ECR11 I do not worry about my relationships* |
| ECR13 When my partner is out of sight, I worry that he or she might become interested in someone else |
| ECR15 When I show my feelings for romantic partners, I’m afraid that they will not feel the same about me |
| ECR17 I rarely worry about my partner leaving me* |
| Avoidant |
| ECR2 I prefer not show a partner how I feel deep down |
| ECR4 I feel comfortable sharing my private thought and feelings with my partner* |
| ECR6 It’s not difficult for me to get close to my partner* |
| ECR8 I am very comfortable being close to romantic partners* |
| ECR10 I don’t feel comfortable opening up to romantic partners |
| ECR12 I prefer not to be too close to romantic partners |
| ECR14 I am nervous when partners get too close to me |
| ECR16 I find it relatively easy to get close to my partner* |
| ECR18 I find it difficult to allow myself to depend on romantic partners |

** Reverse-scored items*

We used a one-level factor model with two latent factors anxious and avoidant attachment as the two factors. In addition, the model includes a common bias factor of response style, with reverse scored items factor loadings set to -1 and other items to 1. The factor was specified to not correlate with the latent factors of anxious and avoidant attachment. This model included one residual correlations (ECR6 ~~ ECR16) which represented closeness and applied this to all groups (UK and Japan), resulting in two residual correlations. The data had high kurtosis and skewness on a Mardia’s test, so we used a robust maximum likelihood estimator [MLR] and provide robust indices for χ², CFI, TLI and RMSEA. When looking at the chi-square separately for the two countries, it appears that model fit for Japan, χ² =406.22, was slightly less acceptable than the model fit for the UK, χ² = 257.37. Goodness-of-fit indices showed an excellent fit for the UK but a poor fit for Japan (see Table S6b). Nonetheless, we accepted this initial configural model (see Table S6c for factor loadings) and began the measurement invariance testing process (see Table S6d).

**Table S6b**

*Model Fit Indices for the Two-Factor Model of Attachment (Anxious and Avoidant) with a Common Bias Factor for both Japan and the UK, and the Entire Sample*

| Model | χ² | df | CFI | TLI | SRMR | RMSEA | RMSEA 90% CI |  |
| --- | --- | --- | --- | --- | --- | --- | --- | --- |
| Japan | 431.00^***^ | 133 | .844 | .820 | .093 | .093 | (.083, .103) |  |
| UK | 271.17^***^ | 133 | .943 | .934 | .054 | .061 | (.050, .071) |  |
| Overall | 653.59^***^ | 264 | .910 | .895 | .068 | .074 | (.067, .081) |  |
| *^***^p < .001.* | | | | | | | | |

**Table S6c**

*Study 1: Factor Loadings for the Two-Factor Model of Attachment (Anxious and Avoidant) with a Common Bias Factor at the Configural Level of Measurement Invariance*

| Factor | Item | UK | | Japan | |
| --- | --- | --- | --- | --- | --- |
|  |  | B (SE) | β | B (SE) | β |
| Anxious | ECR1 | 1.00 | .78 | 1.00 | .76 |
|  | ECR3 | 1.03^***^ (.06) | .81 | .93^***^ (.08) | .72 |
|  | ECR5 | 1.13^***^ (.06) | .81 | 1.02^***^ (.06) | .79 |
|  | ECR7 | .95^***^ (.08) | .73 | 1.04^***^ (.08) | .79 |
|  | ECR9 | .84^***^ (.08) | .62 | .46^***^ (.08) | .37 |
|  | ECR11 | .68^***^ (.08) | .55 | .70^***^ (.09) | .50 |
|  | ECR13 | .93^***^ (.07) | .68 | .85^***^ (.07) | .62 |
|  | ECR15 | .86^***^ (.07) | .69 | 1.02^***^ (.07) | .77 |
|  | ECR17 | .77^***^ (.08) | .62 | .86^***^ (.08) | .68 |
| Avoidant | ECR2 | 1.00 | .68 | 1.00 | .64 |
|  | ECR4 | .92^***^ (.07) | .75 | .95^***^ (.11) | .59 |
|  | ECR6 | .79^***^ (.10) | .56 | .87^***^ (.10) | .56 |
|  | ECR8 | 1.11^***^ (.09) | .82 | .76^***^ (.10) | .60 |
|  | ECR10 | 1.07^***^ (.07) | .76 | 1.22^***^ (.10) | .72 |
|  | ECR12 | 1.00^***^ (.08) | .72 | .99^***^ (.12) | .66 |
|  | ECR14 | 1.04^***^ (.09) | .70 | .78^***^ (.12) | .51 |
|  | ECR16 | .99^***^ (.08) | .73 | .86^***^ (.10) | .52 |
|  | ECR18 | .72^***^ (.10) | .48 | .60^***^ (.10) | ..37 |
| Covariances |  |  |  |  |  |
| ECR6~~ ECR16 |  | .17^***^ (.05) | .24 | .40^***^ (.07) | .43 |
| Anxious ~~ Avoidant |  | .20^***^ (.06) | .27 | .18^***^ (.06) | .28 |

**Table S6d**

*The Measurement Invariance Testing Process for the Two-Factor (Anxious and Avoidant) Model of Attachment with a Common Bias Factor. Table Shows Robust Model Fit Indices at Different Levels of Measurement Invariance*

| Model | | χ²(df) | Δχ²(df) | CFI | TLI | SRMR | RMSEA | | RMSEA 90% CI | Decision |  |
| --- | --- | --- | --- | --- | --- | --- | --- | --- | --- | --- | --- |
| Configural | | 653.59(264)^***^ |  | .910 | .895 | .068 | .074 | (.067, .081) | | Accept |  |
| Metric | | 705.50(280)^***^ | 53.37(16) ^***^ | .902 | .893 | .079 | .075 | (.068, .082) | | Accept |  |
| Scalar | | 958.97(295)^***^ | 287.31(815 ^***^ | .848 | .842 | .087 | .091 | (.084, .097) | | Reject |  |
| Partial Scalar (ECR1,ECR3, ECR4, ECR7, ECR14, ECR15, & ECR17 intercepts varied) | | 749.40(288)^***^ | 47.45(8) ^***^ | .894 | .887 | .080 | .077 | (.70, .083) | | Accept |  |
|  | *^***^p < .001.* | | | | | | | | | | |

As noted in Table S6d, the model was accepted at metric level of invariance testing with no additional modifications needed (see Table S6e for factor loadings).

**Table S6e**

*Study 1: Intercepts for the Two-Factor (Anxious and Avoidant) Model of Attachment with a Common Bias Factor at the Metric of Measurement Invariance*

| Factor | Item | B (SE) |  | |
| --- | --- | --- | --- | --- |
|  |  |  | UK β | Japan β |
| Anxious | ECR1 | 3.32^***^ (.07) | 2.85 | 2.82 |
|  | ECR3 | 3.26^***^ (.07) | 2.90 | 2.56 |
|  | ECR5 | 2.95^***^ (.07) | 2.40 | 2.59 |
|  | ECR7 | 3.52^***^ (.07) | 2.93 | 2.73 |
|  | ECR9 | 3.19^***^ (.07) | 2.78 | 3.08 |
|  | ECR11 | 3.49^***^ (.06) | 3.11 | 2.73 |
|  | ECR13 | 2.80^***^ (.07) | 2.30 | 2.50 |
|  | ECR15 | 3.38^***^ (.06) | 2.85 | 2.87 |
|  | ECR17 | 3.17^***^ (.06) | 2.76 | 3.06 |
| Avoidant | ECR2 | 2.73^***^ (.07) | 2.23 | 2.61 |
|  | ECR4 | 2.33^***^ (.06) | 2.29 | 2.47 |
|  | ECR6 | 2.72^***^ (.07) | 2.27 | 2.40 |
|  | ECR8 | 2.45^***^ (.07) | 2.29 | 2.17 |
|  | ECR10 | 2.65^***^ (.07) | 2.22 | 2.31 |
|  | ECR12 | 2.34^***^ (.07) | 2.01 | 2.13 |
|  | ECR14 | 2.74^***^ (.07) | 2.28 | 2.05 |
|  | ECR16 | 2.55^***^ (.06) | 2.30 | 2.33 |
|  | ECR18 | 3.12^***^ (.07) | 2.57 | 2.45 |
| Covariances |  |  |  |  |
| ECR6 ~~ ECR16 |  | UK: .17^**^ (.05) Japan: .40^***^ (.07) | .24 | .43 |
| Anxious ~~ Avoidant |  | UK: .21^***^ (.06) Japan: .15^**^ (.05) | .27 | .25 |

Although we achieved both configural and metric measurement invariance with relative ease, the model failed Chen’s criteria of goodness-of-fit indices (see Table S6d) and did not achieve scalar invariance (see Table S6f for intercepts). We therefore investigated if releasing intercepts would improve model fit.

**Table S6f**

*Study 1: Intercepts for the Two-Factor (Anxious and Avoidant) Model of Attachment with a Common Bias Factor at the Scalar Level of Measurement Invariance*

| Factor | Item | Intercept | Standardized Intercepts | |
| --- | --- | --- | --- | --- |
|  |  |  | UK | Japan |
| Anxious | ECR1 | 3.32^***^ (.06) | 2.85 | 2.90 |
|  | ECR3 | 3.18^***^ (.06) | 2.82 | 2.70 |
|  | ECR5 | 3.03^***^ (.07) | 2.47 | 2.62 |
|  | ECR7 | 3.33^***^ (.07) | 2.72 | 2.90 |
|  | ECR9 | 3.40^***^ (.06) | 2.82 | 2.93 |
|  | ECR11 | 3.44^***^ (.06)\| | 3.06 | 2.83 |
|  | ECR13 | 2.95^***^ (.06) | 2.42 | 2.42 |
|  | ECR15 | 3.32^***^ (.06) | 2.79 | 2.98 |
|  | ECR17 | 3.32^***^ (.06) | 2.88 | 3.00 |
| Avoidant | ECR2 | 2.85^***^ (.06) | 2.31 | 2.48 |
|  | ECR4 | 2.48^***^ (.06) | 2.42 | 2.02 |
|  | ECR6 | 2.67^***^ (.06) | 2.23 | 2.39 |
|  | ECR8 | 2.29^***^ (.06) | 2.13 | 2.29 |
|  | ECR10 | 2.68^***^ (.06) | 2.25 | 2.24 |
|  | ECR12 | 2.30^***^ (.06) | 1.97 | 2.15 |
|  | ECR14 | 2.57^***^ (.07) | 2.12 | 2.17 |
|  | ECR16 | 2.65^***^ (.06) | 2.39 | 2.13 |
|  | ECR18 | 3.03^***^ (.06) | 2.49 | 2.51 |
| Attachment | Anxious | -.09 (.08) |  | -.11 |
|  | Avoidant | .02 (.08) |  | .03 |
| Common Factor Bias | Style | -.00 (.03) |  | -.00 |
| Covariances |  |  |  |  |
| ECR6 ~~ ECR16 |  | UK: .17^***^ (.06) Japan: .41^***^ (.07) | .23 | .42 |
| Anxious ~~ Avoidant |  | UK: .21^***^ (.06), Japan: .16^**^ (.05) | .27 | .26 |

Anxious *p* = .251, Avoidant *p*= .801, Style *p* = .994.

Modification indices suggested that we should release seven intercepts. It appears that for a given level of anxious attachment, British participants scored slightly higher on ECR1, ER3, ER7, and ER17. Whereas, for a given level of avoidant attachment, Japanese participants scored higher on ECR4 and ECR14. In addition, Japanese participants also scored higher on ECR17 on a given level of avoidant attachment. It becomes clear from the number of intercepts freed from anxious attachment, that the avoidant attachment items are much more culturally similar than the anxious attachment items. After sequentially releasing these intercepts, we achieved partial-scalar invariance (see Table S6g for intercepts). The intercepts suggested Japan and the UK neither not differ on anxious attachment (-.02 *p* = .804) nor avoidant attachment (-.08, *p* = .3241). In addition, the analysis of the common bias factor of response style suggested that Japanese participants were more likely to demonstrate response style bias than participants from the UK (.17, *p* < .001).

**Table S6g**

*Study 1: Intercepts for the Two-Factor (Anxious and Avoidant) Model of Attachment with a Common Bias Factor at the Partial-scalar Level of Measurement Invariance*

| Factor | Item | Intercept | Standardized Intercepts | |
| --- | --- | --- | --- | --- |
|  |  |  | UK | Japan |
| Anxious | ECR1 | UK: 3.32^***^ (.07) Japan: 3.08^***^ (.08) | 2.85 | 2.69 |
|  | ECR3 | UK: 3.26^***^ (.07) Japan: 2.84^***^ (.08) | 2.90 | 2.43 |
|  | ECR5 | 2.89^***^ (.07) | 2.35 | 2.49 |
|  | ECR7 | UK: 3.52^***^ (.07) Japan: 2.94^***^ (.08) | 2.93 | 2.60 |
|  | ECR9 | 3.29^***^ (.06) | 2.86 | 2.85 |
|  | ECR11 | 3.49^***^ (.06)\| | 3.11 | 2.88 |
|  | ECR13 | 2.83^***^ (.06) | 2.33 | 2.34 |
|  | ECR15 | UK: 3.38^***^ (.06) Japan: 3.03^***^ (.08) | 2.85 | 2.73 |
|  | ECR17 | UK: 3.17^***^ (.06) Japan: 3.56^***^ (.08) | 2.76 | 3.23 |
| Avoidant | ECR2 | 2.81^***^ (.06) | 2.29 | 2.46 |
|  | ECR4 | UK: 2.33^***^ (.05) Japan: 3.13^***^ (.08) | 2.29 | 2.69 |
|  | ECR6 | 2.82^***^ (.06) | 2.35 | 2.50 |
|  | ECR8 | 2.42^***^ (.06) | 2.26 | 2.46 |
|  | ECR10 | 2.66^***^ (.06) | 2.23 | 2.23 |
|  | ECR12 | 2.26^***^ (.06) | 1.93 | 2.11 |
|  | ECR14 | UK: 2.74^***^ (.07) Japan: 2.93^***^ (.08) | 2.28 | 1.97 |
|  | ECR16 | 2.62 (.06) | 2.37 | 2.12 |
|  | ECR18 | 2.98 (.06) | 2.43 | 2.45 |
| Attachment | Anxious | -.02 (.08) |  | -.02 |
|  | Avoidant | -.08 (.07) |  | -.12 |
| Common Factor Bias | Style | .17^***^ (.03) |  | .76 |
| Covariances |  |  |  |  |
| ECR6 ~~ ECR16 |  | UK: .18^**^ (.05) Japan: .43^***^ (.07) | .18 | .43 |
| Anxious ~~ Avoidant |  | UK: .21^***^ (.06), Japan: .15^**^ (.05) | .27 | .25 |

Anxious *p* = .804, Avoidant *p*= .241, Style *p* < .001.

**References**

Billiet, J. B., & McClendon, M. J. (2000). Modeling acquiescence in measurement models for two balanced sets of items. *Structural Equation Modeling*, *7*(4), 608-628.

Chen, F. F. (2007). Sensitivity of goodness of fit indexes to lack of measurement invariance. *Structural Equation Modeling: a Multidisciplinary Journal*, *14*(3), 464-504.

Chen, F., Bollen, K. A., Paxton, P., Curran, P. J., & Kirby, J. B. (2001). Improper solutions in structural equation models: Causes, consequences, and strategies. *Sociological Methods & Research*, *29*(4), 468-508.

Cheung, G. W., & Rensvold, R. B. (2000). Assessing extreme and acquiescence response sets in cross-cultural research using structural equations modeling. *Journal of Cross-Cultural Psychology*, *31*(2), 187-212.

Choi, I., Koo, M., & Choi, J. A. (2007). Individual differences in analytic versus holistic thinking. *Personality and Social Psychology Bulletin*, *33*(5), 691-705.

Fischer, R., Fontaine, J., van de Vijver, F. J. R., & van Hemert, D. (2009). What is Style and What is Bias in Cross-Cultural Comparisons? An Examination of Response Styles in Cross-Cultural Research. In A. Gari & K. Mylonas (eds), Quod Erat Demonstrandum: From Herodotus’ Ethnographic Journeys to Cross-Cultural Research (pp.137-148). Athens: Pedio

French, B. F., & Finch, W. H. (2008). Multigroup confirmatory factor analysis: Locating the invariant referent sets. *Structural Equation Modeling: A Multidisciplinary Journal*, *15*(1), 96-113.

Howlett, P., Baysu, G., Atkinson, A. P., Jungert, T., & Rychlowska, M. (2023). Friendship habits questionnaire: A measure of group-versus dyadic-oriented socializing styles. *Plos one*, *18*(6), e0285767.

Hu, L. T., & Bentler, P. M. (1999). Cutoff criteria for fit indexes in covariance structure analysis: Conventional criteria versus new alternatives. *Structural Equation Modeling: A Multidisciplinary Journal*, *6*(1), 1-55.

Johnson, T. P., Shavitt, S., & Holbrook, A. L. (2011). Survey response styles across cultures. In D. Matsumoto & F. J. R. van de Vijver (Eds.), *Cross-cultural research methods in psychology* (pp. 130–178). Cambridge, MA: Cambridge University Press.

Putnick, D. L., & Bornstein, M. H. (2016). Measurement invariance conventions and reporting: The state of the art and future directions for psychological research. *Developmental Review*, *41*, 71-90.

Schneider, B. A., Avivi-Reich, M., & Mozuraitis, M. (2015). A cautionary note on the use of the Analysis of Covariance (ANCOVA) in classification designs with and without within-subject factors. *Frontiers in Psychology*, 6, 474.

Tarling, R. (2008). *Statistical modelling for social researchers: Principles and practice*. Routledge.

Thomson, R., Yuki, M., Talhelm, T., Schug, J., Kito, M., Ayanian, A. H., ... & Ferreira, C. M. (2018). Relational mobility predicts social behaviors in 39 countries and is tied to historical farming and threat. *Proceedings of the National Academy of Sciences*, *115*(29), 7521-7526.
